# Identifying subgroups of patients using latent class analysis: should we use a single-stage or a two-stage approach? A methodological study using a cohort of patients with low back pain

**DOI:** 10.1186/s12891-017-1411-x

**Published:** 2017-02-01

**Authors:** Anne Molgaard Nielsen, Peter Kent, Lise Hestbaek, Werner Vach, Alice Kongsted

**Affiliations:** 1 Department of Sports Science and Clinical Biomechanics, University of Southern Denmark, 5230 Odense M, Denmark; 2 School of Physiotherapy and Exercise Science, Curtin University, Perth, Australia; 3 Nordic Institute of Chiropractic and Clinical Biomechanics, University of Southern Denmark, 5230 Odense M, Denmark; 4 Institute for Medical Biometry and Statistics, Faculty of Medicine and Medical Center - University of Freiburg, 79104 Freiburg, Germany

**Keywords:** Low back pain, Classification, Data mining, Latent class analysis, Subgrouping

## Abstract

**Background:**

Heterogeneity in patients with low back pain (LBP) is well recognised and different approaches to subgrouping have been proposed. Latent Class Analysis (LCA) is a statistical technique that is increasingly being used to identify subgroups based on patient characteristics. However, as LBP is a complex multi-domain condition, the optimal approach when using LCA is unknown. Therefore, this paper describes the exploration of two approaches to LCA that may help improve the identification of clinically relevant and interpretable LBP subgroups.

**Methods:**

From 928 LBP patients consulting a chiropractor, baseline data were used as input to the statistical subgrouping. In a *single-stage LCA*, all variables were modelled simultaneously to identify patient subgroups. In a *two-stage LCA*, we used the latent class membership from our previously published LCA within each of six domains of health (activity, contextual factors, pain, participation, physical impairment and psychology) (first stage) as the variables entered into the second stage of the two-stage LCA to identify patient subgroups. The description of the results of the *single-stage* and *two-stage LCA* was based on a combination of statistical performance measures, qualitative evaluation of clinical interpretability (face validity) and a subgroup membership comparison.

**Results:**

For the *single-stage LCA*, a model solution with seven patient subgroups was preferred, and for the *two-stage LCA*, a nine patient subgroup model. Both approaches identified similar, but not identical, patient subgroups characterised by (i) mild intermittent LBP, (ii) recent severe LBP and activity limitations, (iii) very recent severe LBP with both activity and participation limitations, (iv) work-related LBP, (v) LBP and several negative consequences and (vi) LBP with nerve root involvement.

**Conclusions:**

Both approaches identified clinically interpretable patient subgroups. The potential importance of these subgroups needs to be investigated by exploring whether they can be identified in other cohorts and by examining their possible association with patient outcomes. This may inform the selection of a preferred LCA approach.

**Electronic supplementary material:**

The online version of this article (doi:10.1186/s12891-017-1411-x) contains supplementary material, which is available to authorized users.

## Background

Non-specific low back pain (LBP) [1] is a challenge for patients, clinicians and researchers. Many patients consider a non-specific diagnosis to be insufficient [2, 3]. Clinicians face the dilemma of having to treat patients in the absence of diagnostic and prognostic certainty [4], and there is a lack of strong evidence to guide clinicians in effectively targeting treatment and management [5, 6]. Effects of back pain interventions are typically modest [7–9] and identified prognostic factors only explain a small amount of the variance in a range of outcomes [10, 11].

One possible explanation for the uninspiring treatment effects and difficulties with predicting outcomes is that a ‘one-size-fits-all’ approach may be inappropriate, as non-specific LBP is not a homogenous condition but instead, is comprised of a number of underlying conditions [12].

For more than 25 years, the biopsychosocial model [13] has been generally accepted as a key conceptual framework for explaining the complex interplay between the biological, psychological and social domains of the LBP experience. Despite this, much research conducted during this period has not applied the biopsychosocial model, as studies often focus on only one aspect of the model [14]. One reason for this could be uncertainties about how to handle the complexity and volume of data that can arise as a consequence of this expanded focus. However, such work is possible. For example, one of the very few multi-domain tools that has been developed for targeting LBP treatment is the STarT Back Tool. It is a stratification tool which traverses the pain, activity limitation and psychology domains using a simple 9-item questionnaire to guide management of the heterogeneity in LBP [6]. Although this has some promise for improving treatment effects and there are also models for estimating LBP prognoses [15], much of the heterogeneity in LBP patients is still poorly understood [16].

With the availability of high-speed computers, increasingly advanced software is available to handle and analyse complex data. One such method is Latent Class Analysis (LCA) [17], which can be used to search for relationships between cross-sectional variables without knowing anything about the outcome (unsupervised analysis). It has the potential to identify similar patterns of responses to questionnaire items (as in our case) and thereby identify subgroups of patients who are homogenous in their baseline clinical presentation. Ideally, the data selected for input for this subgrouping would tap into all the health domains relevant to the understanding of LBP. As the number of subgroups is unknown a priori and there are no hypotheses about subgroup characteristics, LCA is used as an explorative tool to identify the subgroup model that best reflects the multidimensional data structure inherent in the patient sample.

A central element of performing LCA is to choose a preferred model from all the estimated models, such as models that differ in their number of subgroups. This model evaluation is commonly performed using a combination of statistical fit indices and conceptual considerations. Statistical fit indices provide information about a statistically optimal number of subgroups, usually based on information criteria such as the Bayesian Information Criterion (BIC) that is a measure balancing model fit and model parsimony. Conceptual considerations (the model’s apparent usefulness) are based on the research question, classification accuracy and clinical interpretability [18–20]. Generally, the subgroup solutions with scoring patterns that show qualitative differences between subgroups are an indication of more clinically distinct characteristics than those that show differences only on condition severity (quantitative differences) [21].

Within the LBP research area, LCA has not previously been conducted on very complex data that represent multiple dimensions [22–25] and as a consequence, it is unknown how this will affect the model solutions and their resulting subgroups. Therefore, some unanswered questions remain about the optimal methodological approach. One consideration is whether the customary approach of entering all data simultaneously in a single step is ideal, or whether it is more feasible to run the LCA first within health domains and subsequently across domains in a second step. This is collectively referred to as a two-stage LCA approach and has previously been illustrated using chest pain data [26].

It appears that this two-stage approach potentially reduces model complexity and improves model parsimony due to fewer variables entering the second-stage LCA and with all variables being categorical in that stage. Additionally, it potentially increases clinical interpretability as the interpretation of the identified subgroups is based on the descriptive labels that the identified domain-specific patient categories were given after the first stage of the two-stage LCA, rather than interpreting all variables at once after a single-stage LCA.

Therefore, the objective of this study was to explore the application of LCA when using baseline characteristics from LBP patients with two different approaches: (1) *single-stage LCA*, where all baseline characteristics were entered simultaneously to identify *patient subgroups* and (2) *two-stage LCA*, where *domain-specific patient categorisations* resulting from an LCA within each of six health domains (first stage) were used as variables in a subsequent LCA to identify *patient subgroups* (second stage). The results obtained from the two approaches were compared using a range of statistical and clinical criteria.

## Methods

### Brief method summary

This study used cross-sectional (baseline) data from a longitudinal observational study of adult patients who were consulting chiropractors in Denmark due to their LBP. Two approaches for LCA subgrouping were compared: one strategy using ‘*single-stage LCA*’ and another using ‘*two-stage LCA*’ [26]. Patient self-reported and clinician-reported questionnaire data were used as inputs to this statistical subgrouping. In the *single-stage LCA*, all variables were entered into the analysis simultaneously. In the *two-stage LCA*, identical baseline variables had been used in a previous first stage LCA [27] to identify domain-specific patient categorisations within six domains of health (activity, contextual factors, pain, participation, physical impairment and psychology), and these categorical variables comprised the input to the second stage LCA. The descriptive comparison of the resulting patient subgroups from the *single-stage* and *two-stage LCA* was based on a combination of statistical performance measures, qualitative evaluation of clinical interpretability (face validity) and a subgroup membership comparison.

### Setting and participants

As part of the research network of the Nordic Institute for Chiropractic and Clinical Biomechanics [28], 17 chiropractic practices collected the data from September 2010 to January 2012. Further information about the cohort study has been reported previously [29–32].

The inclusion criteria were: LBP with or without leg pain as the main complaint, age between 18 and 65 years, access to a mobile phone and ability to send a text message (for reasons unrelated to this paper), and ability to adequately read and write Danish. The exclusion criteria were: pregnancy, pathology of the back that required referral for acute surgical assessment or other serious pathology, or more than one consultation for LBP during the previous 3 months. For our specific analyses, we also excluded patients if no data were available for either the patient-reported or clinician-reported baseline questionnaire.

In total, 970 patients agreed to participate, of which 947 fulfilled the inclusion criteria and provided informed written consent, 19 were excluded due to completely missing data on either the patient-reported or clinician-reported questionnaire. As a result, 928 patients were included in the study, which, based on an extrapolation of the sample size calculations of Wurpts et al. [33], is likely to be sufficient for LCA models with up to 18 subgroups.

The Danish Data Protection Agency approved this study (ref. no. 2012-41-0762) and this study did not need ethics approval under Danish law [34], as treatment was not affected by participation.

### Patient self-reported questionnaire

While attending the clinic, the participants filled in a baseline questionnaire that included pain history, screening questions, personal factors and questionnaires covering activity limitation, depression, fear-avoidance and other known prognostic factors. The variables used in the LCA are reported below:

*Personal factor* variables: sex (male, female), age (years), height (cm), body mass index (BMI) (kg/m^2^), highest educational level (no qualification, vocational training, higher education <3 years, higher education 3–4 years, higher education >4 years), employment status (8 categories), private/work-related health insurance (yes/no), physical workload (sitting, sitting and walking, light physical load, heavy physical load), smoking status (non-smoker, ex-smoker, smoker) and sick leave taken during the previous month (no sick-leave, 1–5 days, 5–31 days).

*Pain history variables*: previous LBP episodes (0, 1–3, >3), duration of current episode (0–2 weeks, 2–4 weeks, 1–3 months, >3 months), days with LBP the preceding year (≤30, >30), typical back pain intensity during the preceding week (0–10 Numeric Pain Rating Scale) and leg pain intensity (0 = no pain, 1 = mild pain, 2 = moderate to severe pain).

*Screening questions*: recovery belief (0 = likely to recover, 1 = unsure about recovery to not at all likely to recover), ability to decrease pain (0–10: 0 = cannot decrease it at all, 10 = can decrease it completely), belief that treatment is essential to decrease pain (0–10: 0 = completely agree; 10 = completely disagree), general health measured by the Visual Analogue Scale of the EQ-5D (0–100: 0 = worst imaginable health state; 100 = best imaginable health state) and social isolation (0 = not at all isolated, 1 = little to quite isolated).

Individual items were used from the following pre-existing *questionnaires*: the STarT Back Tool (SBT) [35, 36], the Danish 23-item version of the Roland Morris Disability Questionnaire (RMDQ-23) [37, 38], the Major Depression Inventory (MDI) [39] and the Fear Avoidance Beliefs Questionnaire (FABQ) [40, 41].

### Clinician-reported questionnaire

The clinicians recorded the additional information from the patient history and carried out a standardised clinical examination [29, 42] as described below:

*Patient history*: back pain is dominating (yes, no), pain distribution (0 = back pain only, 1 = back pain and pain in one leg, 2 = back pain and pain in both legs, 3 = leg pain only), paraspinal pain onset (yes, no), best activity is to walk (yes, no), best posture is to sit (yes, no), any chronic comorbid disease (yes, no), presence of heart/coronary disease (yes, no), presence of asthma/allergy disease (yes, no), presence of depression or other mental disorder (yes, no), presence of musculoskeletal disorder (apart from the low back) (yes, no), or presence of another chronic disease (reporting of any other infrequent disease to be present) (yes, no).

*Posture*: acute lateral shift (yes/no) and acute flexion deformity (yes/no).

*Pain on movement:* pain response on flexion, extension, side glide left and right and rotation left and right (0 = no pain, 1 = back pain, 2 = leg pain with or without back pain) and pain response on combined extension/rotation of the low back (yes, no).

*Repeated end-range movements*: four diagnostic categories based on Mechanical Diagnosis and Therapy as described by McKenzie [43]: reducible disc syndrome (yes, no), partly reducible disc syndrome (yes, no), irreducible disc syndrome (yes, no) and dysfunction syndrome (yes, no).

*Sacroiliac joint (SI joint) tests:* five pain provocation tests (separation test, thigh thrust, Gaenslens test, compression and sacral thrust) (0 = negative test bilaterally and 1 = positive test unilaterally or bilaterally).

*Muscle palpation*: replication of pain on muscle palpation (yes, no), painful muscle group (1 = back muscles inclusive of the Iliopsoas muscle, 2 = buttock and leg muscles, 3 = both back and leg muscles) and replication of pain by trigger points (yes, no).

*Neurological status of the lower extremities*: signs of nerve root involvement right side (yes, no) and left side (yes, no), affected muscular strength (yes, no), sensibility (yes, no) and deep tendon reflexes (yes, no).

Additional details of each variable from the patient-reported and clinician-reported questionnaires, including the sources, are referenced in Additional file 1.

### Categorical variables for the two-stage LCA

The input to the second stage of the two-stage LCA was one domain-specific patient categorisation for each of the six health domains: pain, activity, psychology, participation, physical impairment and contextual factors [44] (Table 1).

**Table 1:** Characteristics of the categorical variables used in the second stage of the two-stage LCA

Latent Class Analysis derived variable (domain-specific patient categories from each health domain)	Prevalence, %	Posterior probability, median (interquartile range)
Activity		0.97
Very high degree of disability (on all features)	27	(0.85-0.99)
Very high degree of disability, but no walking distance limitation	24	
Very low degree of disability	15	
Low degree of disability, but dressing problems	11	
Moderate degree of disability, no walking distance limitations	9	
Low degree of disability, but difficulties in household duties	9	
Moderate degree of disability, high degree of walking limitations (speed and distance)	5	
Contextual factors		1.00
Healthy males working full‐time or self‐employed	33	(0.98-1.00)
Healthy females working full‐time or part‐time	21	
Females with comorbidity, working full‐time or part-time	16	
Males with comorbidity, working full‐time or self-employed	16	
Healthy patients in their 30s, higher BMI, working full-time, student or unemployed	6	
Healthy students (approx. 25 years of age), lower BMI, fewer with health insurance	4	
Retired patients (or working part‐time) with comorbidity, fewer with health insurance	4	
Pain		0.97
Recent LBP with high degree of back pain severity	27	(0.83-0.99)
Recent LBP with high degree of back pain severity and moderate degree of leg pain severity	22	
Recent LBP with low degree of back pain severity	17	
Persistent LBP, high degree of back and leg pain severity	11	
Recent LBP with moderate degree of back pain severity, moderate degree of leg pain severity	10	
Persistent LBP, moderate degree of back pain severity and low degree of leg pain severity	9	
Recent LBP, moderate degree of LBP severity, moderate-high degree of leg pain severity, non-dominating LBP	5	
Participation		0.88
Very low work and SPL, low degree of physical workload	38	(0.66-0.94)
Very low SPL, unsure if work aggravates/makes worse, whichever degree of physical workload	17	
Very low SPL, work is too heavy, aggravates/makes worse, pain caused by or at work, high degree of physical workload	14	
High degree of SPL, but low degree of work limitations whichever degree of physical workload	10	
Moderate degree of SPL, work aggravated/makes pain worse, very low degree of physical workload	8	
Low degree of SPL, pain caused by work, none with very low degree of physical workload	8	
High degree of SPL, work is too heavy, aggravates/makes worse, pain caused by work, high degree of physical workload	6	
Physical impairment		0.98
LBP on flexion and extension, no leg pain	28	(0.87-1.00)
LBP on flexion, extension, and side glide, no leg pain, *SI joint pain*, TrP and painful buttock/leg muscles	19	
LBP on flexion, extension and side glide, no leg pain, *diagnosis: reducible disc*	16	
LBP on flexion, extension and side glide, no leg pain, *diagnosis: partly reducible disc*	15	
LBP on active range of motion in all directions	14	
Leg pain on flexion, extension and side glide, neurological signs, TrP and painful buttock/leg muscles	8	
Psychology		0.97
Treatment believers with low degree of depressive mood	21	(0.88-1.00)
Pain-related concerns, moderate degree of depressive mood	17	
Uncomplicated psychological profile	14	
Sleep well, low degree of depressive mood	13	
Treatment believers with sleep issues and moderate degree of depressive mood	13	
Sleep issues, low degree of pain-related concerns	11	
The complicated psychological profile	6	
Pain-related concerns, low degree of depressive mood	5	

*LCA* Latent Class Analysis, *BMI* Body Mass Index, *LBP* low back pain, *SPL* social participation limitations, *SI* sacroiliac, *TrP* trigger points

**Fig. 1:**
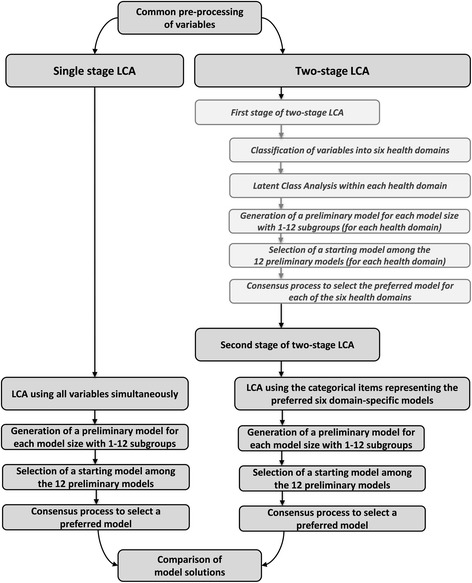
Method flowchart. LCA = Latent Class Analysis. *Italics*: Reported in a previous paper [27]

These categorisations had been derived by LCA (first stage of the two-stage LCA) using all baseline variables described above, as mutually exclusive input to the six health domains. LCA was performed within each of the health domains resulting in the six domain-specific patient categorisations. Based on the category for which their posterior probability was highest, patients were assigned to one of the categories within each health domain [27]. Subsequently, each category was given a descriptive label based on the main distribution of characteristics that distinguished each category from the others in the same health domain, using terms such as ‘more unemployed’, ‘higher BMI’ or by describing the span of the most frequently observed ages. However, this labelling should not be interpreted as absolute criteria for belonging to each category, as people who were outside of these broad descriptive criteria could still be included in the subgroup, for example people outside of the label’s age range could also be in this category. Therefore the labels described the broad distribution of that characteristic within the subgroup and were to be used for the interpretation of the final patient subgroups. LCA was performed in that study using a method pathway that was identical to that used for the single-stage and the second stage of the two-stage LCA in this paper (Fig. 1).

## Data analyses

### Brief summary of data analysis (the remaining paragraphs in the data analysis section may be unread without loss of continuity, only some loss of detail)

**Fig. 2:**
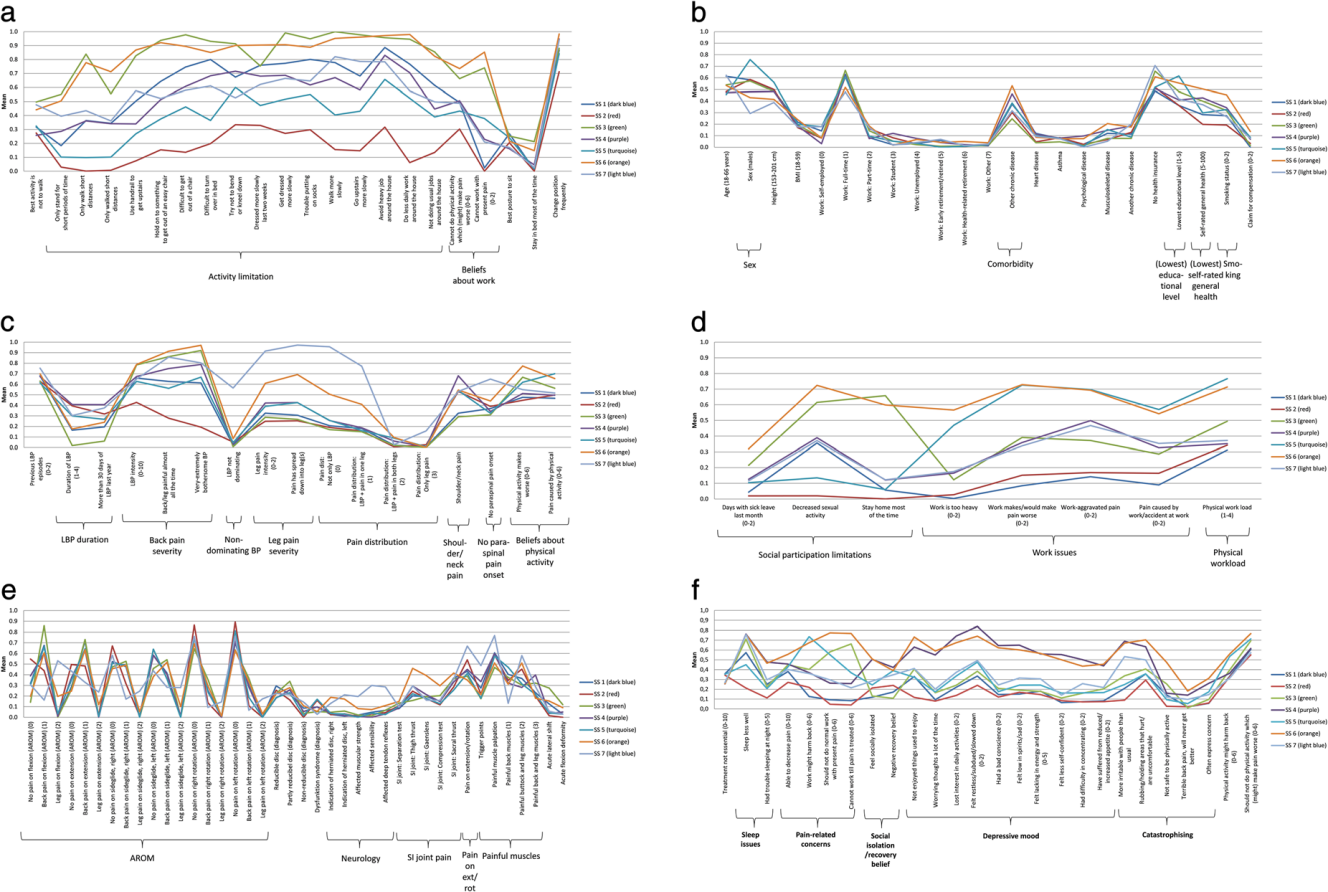
**a** Single-stage patient subgroups based on variables from the activity domain. *Abbreviation:* SS = single-stage patient subgroup.  = *Features*: Two identified representing two to 16 variables with a distinct scoring pattern. *Brief conceptual description* of the single-stage patient subgroups based on variables from the activity domain: SS 1: High degree of disability, low degree of walking distance limitations, can work. SS 2: Very low degree of disability, can work. SS 3: High degree of disability. SS 4: Moderate degree of disability, household challenges. SS 5: Moderate degree of disability, low degree of walking distance limitations. SS 6: High degree of disability. SS 7: Moderate degree of disability, walking (speed) limitations. **b** Single-stage patient subgroups based on variables from the contextual factors domain. *Abbreviations:* BMI = Body Mass Index. SS = single-stage patient subgroup.  = *Features*: Five identified representing one variable each with a slightly distinct scoring pattern. *Brief conceptual description* of the single-stage patient subgroups based on variables from the contextual factors domain was not considered appropriate as contextual factors did not contribute to the interpretation. **c** Single-stage patient subgroups based on variables from the pain domain. *Abbreviations:* LBP = low back pain. SS = single-stage patient subgroup.  = *Features*: Eight identified representing one to three variables with a distinct scoring pattern. *Brief conceptual description* of the single-stage patient subgroups based on variables from the pain domain. SS 1: Recent LBP with high degree of back pain severity. SS 2: Intermittent LBP with moderate degree of back pain severity. SS 3: Recent LBP with high degree of back pain severity, worsened by physical activity. SS 4: Persistent LBP with high degree of back pain severity and shoulder/neck pain. SS 5: Recent LBP with high degree of back pain severity and low degree of leg pain severity. SS 6: Recent LBP with very high back pain severity and moderate degree of leg pain severity, worsened by physical activity. SS 7: Dominating leg pain, high degree of leg pain severity, no paraspinal pain onset. **d** Single-stage patient subgroups based on variables from the participation domain. *Abbreviations:* SS = single-stage patient subgroup.  = *Features*: Three identified representing one to four variables with a distinct scoring pattern. *Brief conceptual description* of the single-stage patient subgroups based on variables from the participation domain: SS 1: Moderate degree of social participation limitations. SS 2: Low degree of participation limitations. SS 3: High degree of social participation limitations, moderate degree of work issues. SS 4: Moderate degree of participation limitations. SS 5: High degree of work issues and physical workload. SS 6: High degree of participation limitations and physical workload. SS 7: Moderate degree of participation limitations. **e** Single-stage patient subgroups based on variables from the physical impairment domain. *Abbreviations:* AROM = active range of motion. LBP = low back pain. SI = sacroiliac. SS = single-stage patient subgroup.  *= Features*: None, but five conceptually related items were identified. *Brief conceptual description* of the single-stage patient subgroups based on variables from the physical impairment domain: SS 1: LBP on flexion, extension and side glide, painful back muscles. SS 2: Low degree of pain on AROM, painful buttock and leg muscles. SS 3: LBP on AROM. SS 4: LBP on flexion, extension and side glide, painful back, buttock and leg muscles. SS 5: LBP on flexion, extension and side glide, painful back muscles. SS 6: LBP and leg pain on AROM, SI joint pain, painful buttock and leg muscles. SS 7: Leg pain on AROM, neurological signs, pain on extension/rotation, trigger points. **f** Single-stage patient subgroups based on variables from the psychology domain. *Abbreviation:* SS = single-stage patient subgroup.  = *Features*: Five identified representing two to 10 variables with a distinct scoring pattern. *Brief conceptual description* of the single-stage patient subgroups based on variables from the psychology domain. SS 1: Sleep issues. SS 2: The uncomplicated psychological profile. SS 3: Pain-related concerns and sleep issues. SS 4: Psychologically affected without pain-related concerns. SS 5: Pain-related concerns and negative recovery beliefs. SS 6: The complicated psychological profile. SS 7: Sleep issues and catastrophizing

The result of an LCA is called a model and it contains a pre-specified number of subgroups. After running an analysis, statistical measures are given for each model and its resulting subgroups. For the two LCA approaches, we performed a series of LCAs that resulted in a number of subgroups from one to 12. From these ‘preliminary models’ we chose a ‘starting model’ as the starting point for the model selection, based on statistical model fit (BIC [45]). Subsequently, a preferred model for each LCA approach was selected by comparing the starting model with the larger preliminary models (those containing more subgroups) using a consensus-based approach that included (i) inspection of the statistical measures, (ii) a graphical presentation of the models showing the characteristics for each subgroup’s profile plots (for an example, see Fig. 2a, Additional file 2) and (iii) a brief clinical description of the subgroups. Lastly, the preferred models from the *single-stage LCA* and one for the *two-stage LCA* were compared by describing and inspecting the subgroups more thoroughly and by comparing the participants’ assignment to the specific patient subgroups. As shown in Fig. 1, four identical steps were used for each stage reported here (the single-stage LCA and the two stages of the two-stage LCA). Further information about the first stage of the two-stage LCA is available on request from the first author.

### Pre-processing of variables

The MDI and FABQ data contained some items with six or seven response options that we decided to exclude if more than 85% of all people scored in only one category. If the data distributions were highly skewed on either ordinal or continuous scales, they were re-scored into categorical variables. Within the clinician-reported questionnaire, dichotomous items were pooled if it made sense from a clinical perspective. For example, the SI joint variables were pooled into one variable for each test, thereby replacing side-specific variables. As the likelihood approach used in LCA can manage the inclusion of patients with missing values, no data were imputed [46]. Any reverse scoring, any recoding into categories and all rates of missing data are described in Additional file 1.

### Latent class analysis

For both LCA approaches, three common steps were used before the preferred model solutions were compared: (1) generation of preliminary models, (2) selection of a starting model and, (3) selection of the preferred model.

### Generation of a preliminary model for a given number of subgroups and deciding the starting model

The LCA procedure was run to generate a preliminary model for each model size from one to 12 subgroups. For each model size, 10 repetitions were made with random seeds (numerical starting points) and the model with the most consistent BIC was used. If none were distinctively consistent or several models with a maximal frequency appeared, the model with the lowest BIC was selected. Among these 12 preliminary models, the model with the lowest BIC was used as the starting model, provided the BIC decreased by at least 1% when adding a subgroup. The other preliminary models were still retained and made available for use in the subsequent consensus process.

### Consensus process to select the preferred models

A preferred model among the models with one to 12 subgroups was selected for each of the LCA analyses using a consensus-based approach that included a number of steps. First, graphical presentations of the subgroups using profile plots (see Fig. 2a for an example, Additional file 2) were compared between the starting model and the remaining preliminary models to explore how the composition of the subgroups altered when adding subgroups, and to identify distinct characteristics [46].

In the exploration of which clinical characteristics were distinctive between subgroups, we were particularly interested in *qualitative* differences, rather than just *quantitative* differences [21]. *Qualitative* differences can be seen by lines crossing on profile plots and these indicate distinctive differences in scoring between the subgroups. For example, two subgroups displaying opposite patterns of scoring on the same variables (i.e. subgroup A scoring high on pain intensity and low on activity limitation, subgroup B scoring low on pain intensity and high on activity limitation). *Quantitative* differences are where the overall pattern of scoring is the same between subgroups (no lines crossing on the profile plots) but the subgroups simply vary in their absolute scores. Typically this would reflect differences in the severity of the condition (for example subgroup A scoring high on pain intensity and activity limitation, subgroup B scoring low on pain intensity and activity limitation).

In addition, we inspected the most appealing candidate models on their: (1) *subgroup size,* as we favoured LCA models in which the smallest subgroup size was at least 5% of the whole cohort (however, as some clinical characteristics are known to be under-represented in this chiropractic cohort compared with the general LBP patient population [long duration of LBP, high intensity leg pain, smoking and self-perceived general health], we further explored subgroup sizes from 3% to 5% if the distinguishing characteristics of the added subgroups included these variables [30]); (2) *conditional probabilities* for categorical and ordinal items (the probability of specific responses given subgroup membership); (3) *conditional means* of ordinal and continuous items; and (4) *loadings* (the correlation between each variable and the identified subgroups) [46].

Lastly, based on the profile plots and the conditional probabilities and conditional means, we wrote a short narrative description of the preferred models, outlining the main characteristics of each subgroup. In the case of the second stage of the two-stage LCA, the previously generated labels, descriptive statistics and profile plots for the domain-specific patient categories were additionally used for this interpretation of the patient subgroups (Additional file 3: Descriptive results of the two-stage LCA)

### Descriptive comparison of patient subgroups derived from the single-stage and two-stage LCA approaches

The preferred subgroup models were descriptively compared to assess the differences resulting from the choice of approach, as both approaches used identical baseline variables. Firstly, posterior probabilities were assessed as a measure of certainty of subgroup membership. The median posterior probability (which theoretically would be 1.00 if there were no uncertainty about a patient’s subgroup membership [47]), the number of participants with posterior probability less than 0.70 *for any subgroup*, and the number of participants with a posterior probability above 0.33 *for more than one subgroup*.

**Fig. 3:**
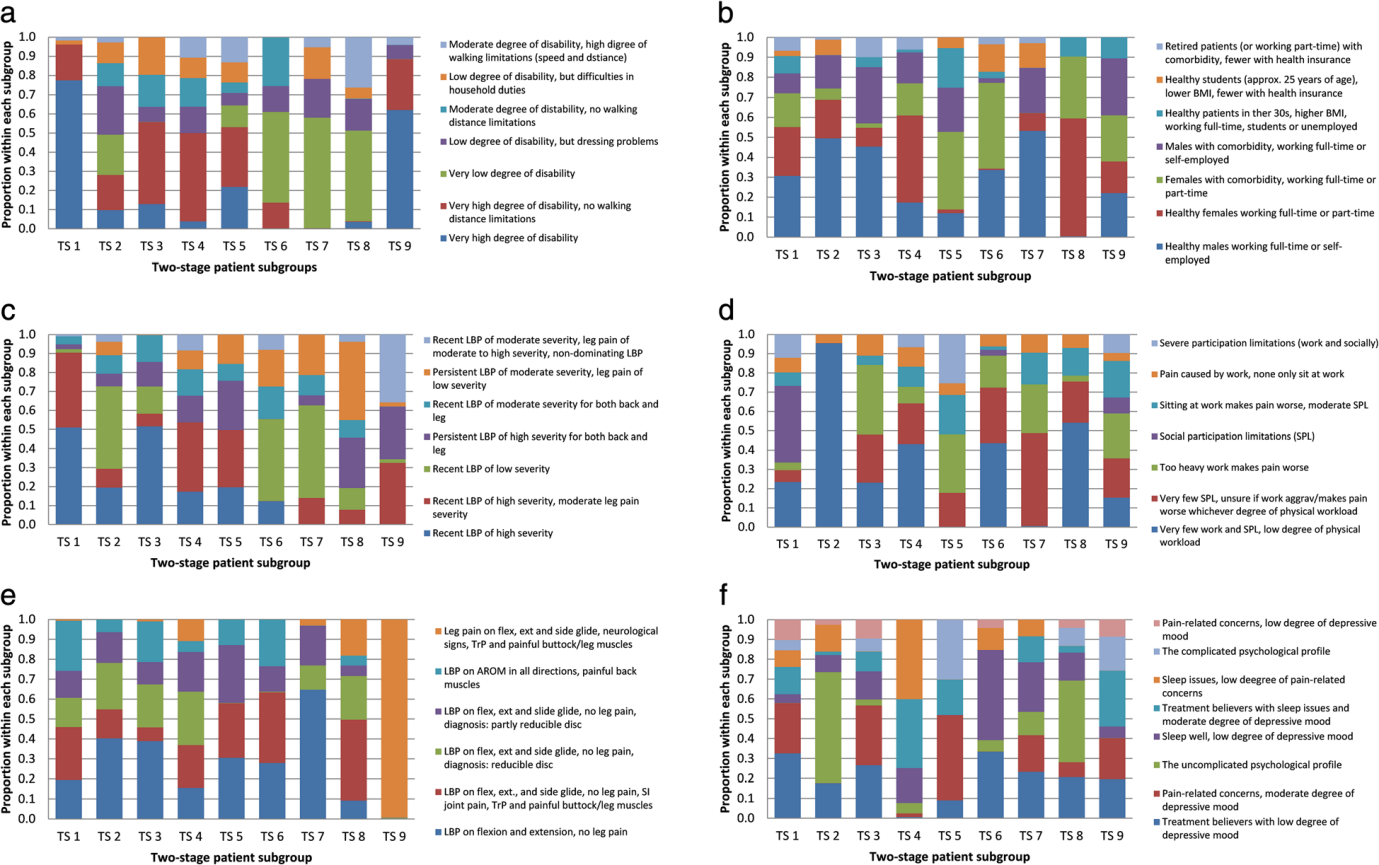
**a** Two-stage patient subgroups based on domain-specific patient categories identified in the activity domain. Stacked bar chart for each patient subgroup based on the conditional probabilities of each domain-specific patient category (the identified latent variables from the first stage Latent Class Analysis in the activity domain). **b** Two-stage patient subgroups based on domain-specific patient categories identified in the contextual factors domain. Stacked bar chart for each patient subgroup based on the conditional probabilities of each domain-specific patient category (the identified latent variables from the first stage Latent Class Analysis in the contextual factors domain). BMI = Body Mass Index. **c** Two-stage patient subgroups based on domain-specific patient categories identified in the pain domain. Stacked bar chart for each patient subgroup based on the conditional probabilities of each domain-specific patient category (the identified latent variables from the first stage Latent Class Analysis in the pain domain). LBP = low back pain. **d** Two-stage patient subgroups based on domain-specific patient categories identified in the participation domain. Stacked bar chart for each patient subgroup based on the conditional probabilities of each domain-specific patient category (the identified latent variables from the first stage Latent Class Analysis in the participation domain). **e** Two-stage patient subgroups based on domain-specific patient categories identified in the physical impairment domain. Stacked bar chart for each patient subgroup based on the conditional probabilities of each domain-specific patient category (the identified latent variables from the first stage Latent Class Analysis in the physical impairment domain). Flex = flexion. Ext = extension. TrP = trigger points. LBP = low back pain. AROM = active range of motion. SI = sacroiliac. **f** Two-stage patient subgroups based on domain-specific patient categories identified in the psychology domain. Stacked bar chart for each patient subgroup based on the conditional probabilities of each domain-specific patient category (the identified latent variables from the first stage Latent Class Analysis in the psychology domain)

Secondly, for the single-stage LCA, the profile plots were used for the identification of subgroup features, which were defined as a group of variables with (i) only quantitative differences (or very minor profile plot crossings) *and* (ii) at least 30% difference between the highest and lowest score of the subgroups. To enhance clinical interpretability, we reverse scored variables where appropriate so that higher scores indicated a more severe condition (detailed in Additional file 1). Because only categorical input was used for the second stage of the two-stage LCA, an alternative representation of the patient subgroups was added that used bar charts (Fig. 3, Additional file 3).

Thirdly, we described the conditional probabilities and means within subgroups, and made a brief clinical description of the characteristics distinguishing each subgroup from the others within each approach. For this description and the subsequent steps, each patient was assigned to the subgroup for which they had the largest posterior probability.

Lastly, a conceptual clinical comparison of patient subgroups was performed across approaches and subgroup membership was subsequently cross-tabulated to quantify the overlap of participants between subgroups from each of the two LCA approaches.

### Statistical software

Latent GOLD 5.0 (Statistical Innovations Inc. Belmont, MA, USA) [46, 48] was used to perform the LCA. Excel 2010 (Microsoft Corporation, Redmond, WA, USA) was used to colour and format the profile plots and bar charts. STATA/SE 13.1 (StataCorp LP, College Station, TX, USA) was used for all other analyses.

## Results

Data were available from 928 participants and 95% of these had more than 86% complete data. Selected baseline characteristics are presented in Table 2 [29, 31]. A total of 112 baseline variables were used in the *single-stage LCA* approach (the same variables as had been used to generate the domain-specific patient categories entering the *two-stage LCA*).

**Table 2:** Baseline characteristics

	Low back pain patients *N* = 928
Age, median years (interquartile range)	43 (34–53)
Females, *N* (%)	418 (45)
Back pain intensity (0–10 Numeric Rating Scale), median (interquartile range) Missing, *N* (%)	7 (5–8) 25 (3)
Any leg pain (mild to severe intensity), *N* (%) Missing, *N* (%)	513 (55) 43 (5)
Duration of current episode of back pain exceeds 3 months, *N* (%) Missing, *N* (%)	121 (13) 18 (2)

### The starting model solutions of the single-stage LCA and the second stage of the two-stage LCA

Both starting model solutions consisted of two subgroups, based only on the statistical criteria defined for the LCA (Additional file 5).

### Results of the consensus process selecting the preferred model for each LCA approach

For the *single-stage LCA*, a model solution with seven patient subgroups and for the *two-stage LCA* a model solution with nine patient subgroups was preferred, thus each consensus process resulted in larger model solutions than the starting models. That is because the smaller models did not include subgroups with the distinct characteristics that were observed in larger models and which appeared to have potential clinical relevance. More specifically, reasons for favouring these preferred models over even larger ones were that (i) larger models simply added subgroups that were minor modifications of existing ones, (ii) larger models included subgroups that could not be clinically interpreted due to such features as contradictory characteristics, and (iii) larger models often included very small subgroups. The consistency of BIC diminished for the larger models but a better model fit (lower BIC) was seen for the preferred model solution in the *single-stage LCA* relative to the starting model (Additional file 5). All of the considered LCA solutions are shown in Additional file 6 (*single-stage LCA*) and Additional file 7 *(second stage of the two-stage LCA*).

### Descriptive comparison of the preferred model solution for both LCA approaches

Participants were reasonably well distributed across the patient subgroups in both the *single-stage* and *two-stage* approach, with none of the patient subgroups containing a majority of participants. Overall, the *single-stage LCA* tended to have higher certainty of subgroup membership compared with the *two-stage LCA,* with a higher proportion of participants clearly assigned to one subgroup as shown by the higher median posterior probability (1.00 for *single-stage LCA* versus 0.91 for *two-stage LCA*) and with fewer participants having a posterior probability below 0.70 (3% versus 20%) (Table 3).

**Table 3:** Statistical measures for the single-stage and two-stage (second stage) Latent Class Analysis approaches

	Single-stage Latent Class Analysis	Two-stage Latent Class Analysis (second stage)
Variables, N	112	6
Identified patient subgroups, N	7	9
Subgroup size range, N	75-192 (8%-21%)	45-219 (5%-24%)
Posterior probability, median (interquartile range)	1.00 (0.99-1.00)	0.91 (0.75-0.99)
Subjects with posterior probability less than 0.70 on average per subgroup, N	4 (3%) Range: 1–7 (1%-5%)	20 (19%) Range: 3–41 (6%-32%)
Subjects with posterior probability above 0.33 for more than one subgroup, N	18 (2%)	81 (9%)

### Single-stage LCA

From a general inspection of the profile plots, it appears that many differences between these subgroups were quantitative (Fig. 2a-f, Additional file 2). However, the two subgroups (SS 6, orange + SS 3, green) that were severely affected on many features did differ qualitatively. For example, patients in the green (SS 1) subgroup were not likely to have leg pain, did not have work issues, and were only psychologically affected on sleep issues and pain-related concerns. Also three of four subgroups with moderately severe back pain and moderate disability had specific characteristics that did not only indicate a continuum of severity. One subgroup was characterised by *leg pain, neurological findings and sleep issues* (SS 7, light blue); one by *persistent LBP, neck/shoulder pain, and a severe psychological profile* except for pain-related concerns (SS 4, purple); and the third by *work issues and pain-related concerns* (SS 5, turquoise). The fourth moderately affected subgroup (SS 1, dark blue) was similar to the green subgroup (SS 3) but only at *a less severe level*, which indicates only quantitative differences. Finally, the red subgroup (SS 2) had *persistent LBP and was generally mildly affected*. The labelling and prevalence of each patient subgroup is presented in Table 4. Identified features are presented within the profile plots (Fig. 2a-f, Additional file 2) and descriptive statistics in Additional file 8.

**Table 4:** Prevalence and summary of the identified patient subgroups

Single-stage LCA		Two-stage LCA	
Prevalence	Description	Prevalence	Description
21%	Recent severe LBP, activity limitations (SS 1, dark blue)	12%	Recent severe LBP, activity limitations, sleep issues (TS 4)
15%	Severely affected: very recent onset severe LBP, social participation and activity limitations (SS 3, green)	24%	Severely affected: very recent onset severe LBP, social participation and activity limitations (TS 1)
14%	Pain- and work-related concerns, high physical workload (SS 5, turquoise)	14%	Work-related severe LBP (TS3)
12%	Severely affected: recent LBP with several consequences (SS 6, orange)	8%	Severely affected: LBP with several consequences (TS 5)
8%	LBP with nerve root involvement (SS 7, light blue)	5%	LBP with nerve root involvement (TS 9)
14%	Persistent LBP, psychological issues, activity limitations and comorbidity (SS 4, purple)		
		5%	Mildly affected: persistent LBP with sacroiliac joint pain (TS 8)
17%	Mildly affected: mild intermittent LBP (SS 2, red)	17%	Mildly affected: mild intermittent LBP, moderate activity limitations, no participation limitations (low degree of physical workload) (TS 2)
		7%	Mildly affected: mild intermittent LBP, sleeps well, moderate activity limitations and sacroiliac joint pain, more females (TS 6)
		8%	Mildly affected: mild intermittent LBP with work-issues, no activity limitations, males (TS 7)

*LCA* Latent Class Analysis, *LBP* low back pain, *SS* single-stage patient subgroup, *TS* two-stage patient subgroup

### Two-stage LCA

The nine patient subgroups in the *two-stage LCA* were distinguished by similar characteristics to those from the *single-stage* but also by results from the SI joint tests and sleep issues (Table 4). We observed that each patient subgroup typically covered several categories from each of the health domains identified in the first step of the *two-stage* approach. In only a few instances did the majority of patients in a subgroup belong to one category: the majority of patients in TS 1 and TS 9 showed a *‘very high degree of disability’* (Fig. 3a, Additional file 4), the majority of patients in TS 2 showed *‘very few work and social participation limitations, low degree of physical workload’* (Fig. 3d, Additional file 4), and the majority of patients in TS 9 showed *‘leg pain on flexion, extension and side glide, neurological signs, trigger points and painful buttock/leg muscles’* (Fig. 3e, Additional file 4).

These nine subgroups represented various levels of severity and also had distinct characteristics. For example, two subgroups were severely affected on many features but differed qualitatively, with one subgroup having few work and psychological issues (TS 1) and another being likely to have comorbidity (TS 5). Two subgroups were severely affected on back pain severity and moderately affected on disability, but differed qualitatively, with one having work-related LBP (TS 3) and the other having leg pain and sleep issues (TS 4). An additional subgroup that was severely affected with back pain differed by having persistent LBP and being severely affected with leg pain but were only mildly affected on other health domains (TS 8). Another subgroup characterised by severe leg pain, differed qualitatively by having nerve root involvement of the lower extremities and severe disability (TS 9). The remaining three subgroups were interpreted as mildly affected on most features, but differed qualitatively in the sense that one had a high degree of disability (TS 2), one was likely to have comorbidity (TS 6) and one had work issues and few physical impairment findings (TS 7). Descriptive statistics of the *two-stage LCA* are presented in Additional file 4.

### Comparison of patient subgroups identified by single-stage and two-stage LCA

As evident from the clinical descriptions presented in Table 4, there are some matches between the subgroups identified in the two approaches at the conceptual level. Table 5 presents a suggestion for a conceptual matching of subgroups, with one subgroup of the *single-stage* solution (SS 2, red) matched to three subgroups of the *two-stage* solution (TS 2, TS 6 and TS 7), and one subgroup from each solution remaining unmatched (SS 4, purple and TS 8). As indicated by the prevalence of the subgroups (Table 4), conceptually similar subgroups did not necessarily identify the same patients. This is corroborated in Table 6, which presents a membership comparison across approaches. On the one hand, we can observe that the largest absolute numbers for overlap typically appear for conceptually similar subgroups, but on the other hand, it generally holds true, that any subgroup from one solution is spread over several - but not all - subgroups of the other solution, with SS 3 (green) being nearly a subset of TS 1 as the only exception. We have tried to retranslate this into the main differences between the matched subgroups, as indicated in Table 5.

**Table 5:** Descriptive differences between patient subgroups which are regarded as quite similar clinically

Single-stage LCA	Two-stage LCA
Recent severe LBP, activity limitations (SS 1, dark blue)	Recent severe LBP, activity limitations, sleep issues (TS 4) • More with sleep issues, moderate degree of depression • More with leg pain • More cannot work • More females
Severely affected: very recent onset severe LBP, social participation and activity limitations (SS 3, green) • More believe they cannot work	Severely affected: very recent onset severe LBP, social participation and activity limitations (TS 1) • More with a higher degree of depression, feel socially isolated • More with a higher degree of leg pain severity (tendency)
Pain- and work-related concerns, high physical work-load (SS 5, turquoise) • More with a higher degree of leg pain severity • More with a longer duration of LBP	Work-related severe LBP (TS 3) • More with a higher degree of depression • More with a higher degree of disability
Severely affected: recent LBP with several consequences (SS 6) • More with a higher degree of pain-related concerns • More with a higher degree of disability • More with social participation limitations • More with leg pain on AROM	Severely affected: LBP with several consequences (TS 5)
LBP with nerve root involvement (SS 7, light blue)	LBP with nerve root involvement (TS 9) • More with a higher degree of depression • More with a higher degree of disability • More with a higher degree of work participation limitations • More with affected neurology • More males • More with comorbidity
Mildly affected: mild intermittent LBP (SS 2, red) • More with any duration of LBP • More with low degree of disability • More with comorbidity	Mildly affected: mild intermittent LBP, moderate activity limitations, no participation limitations (low degree of physical workload) (TS 2) • More with LBP duration of 0–4 weeks • More with only LBP • More males
Mildly affected: mild intermittent LBP (SS 2, red) • More with dressing problems	Mildly affected: mild intermittent LBP, sleeps well, moderate activity limitations and SI joint pain, more females (TS 6) • More with pain-related concern • More with SI joint pain, with trigger points and painful muscles • More with comorbidity
Mildly affected: mild intermittent LBP (SS 2, red) • More with a higher degree of leg pain severity • More with reducible disc (diagnosis) • More with SI joint pain	Mildly affected: mild intermittent LBP with work-issues, no activity limitations, males (TS 7) • More with a higher degree of depression and pain-related concerns • More with decreased sexual activity • More with higher degree of work participation limitations • More males

*LCA* Latent Class Analysis, *SS* single-stage patient subgroup, *TS* two-stage patient subgroup *LBP*, low back pain, *AROM* active range of motion, *SI* sacroiliac

**Table 6:** Patient subgroup membership comparison

	Two-stage patient subgroups										
Single-stage patient subgroups		TS 1	TS 2	TS 3	TS 4	TS 5	TS 6	TS 7	TS 8	TS 9	*Total*
	SS 1 (dark blue)	26^c,d^	79^a^	27^c,d^	36^b,e^	0	14^d^	3	6^d^	1	*192*
	SS 2 (red)	0	69^a,e^	4	2	0	31^b,d,e^	29^b,e^	19^b^	0	*154*
	SS 3 (green)	113^a,e^	3	13^d^	4	2	0	0	1	0	*136*
	SS 4 (purple)	17^c^	3	30^c,d^	28^c,d^	33^a^	1	12^d^	8^d^	0	*132*
	SS 5 (turquoise)	4	4	44^a,e^	14^c,d^	12^d^	20^c,d^	29^b^	2	1	*130*
	SS 6 (orange)	56^a^	0	6	5	22^c,d,e^	0	0	0	20^c^	*109*
	SS 7 (light blue)	3	3	3	24^c,d^	2	3	1	9^c^	27^a,e^	*75*
	*Total*	*219*	*161*	*127*	*113*	*71*	*69*	*74*	*45*	*49*	*928*

*SS* single-stage patient subgroup, *TS* two-stage patient subgroup

^a^The largest subgroup in each row. ^b^The largest subgroup in each column (if different from the largest subgroup in each row). ^c^The number of patients covers more than 10% of the row and/or column subgroup (but is not the largest subgroup). ^d^The number of patients covers more than 10% of the column subgroup (but is not the largest subgroup). ^e^Patient subgroups with similar clinical descriptions

## Discussion

To our best knowledge, this is the first study addressing how multi-domain data that describe LBP can be explored by the application of LCA using both a *single-stage LCA* and a *two-stage LCA* approach*.* In the *two-stage* approach, health domains were used as an intermediate step and it was expected that this approach would reduce model complexity, increase model parsimony and increase clinical interpretability.

Both approaches resulted in subgroups that appeared to represent distinct subtypes of LBP rather than just a continuum of severity or complexity. These subgroups displayed recognisable characteristics, but represented patient profiles that were challenging to describe with short labels using a consistent method. The expectation that the *two-stage* approach might result in patient subgroups that were simpler to describe clinically than the *single-stage* approach was not supported by the results. The complexity arose as only few conditional probabilities within each domain-specific patient categorisation were markedly above or below 0.50 and thereby, the interpretations were often based on two or more descriptive labels (domain-specific patient categories) from each health domains. However, this might reflect the multidimensional nature of LBP and our expectation of reduced complexity might have been both too optimistic and too focused on creating simple labels for each subgroup with the aim of making subgroups that would be easily recognisable in clinical settings. The identified profiles were still considered potentially clinical relevant and the results fit the general recognition that LBP is highly complex.

Summarised briefly, the identified patient subgroups from both LCA approaches could be described as two subgroups being severely affected, and one (*single-stage*) or four (*two-stage*) subgroups being mildly affected, with both mildly and severely affected subgroups showing diversity on other characteristics. Among the remaining subgroups, one was characterised by signs of nerve root involvement and another by work-related issues. In addition, the *single-stage* approach identified a subgroup with distinct characteristics on LBP duration, psychological issues, disability and comorbidity. Overall, there were similarities in the results of the two approaches, which indicate that the two LCA approaches detected a similar, but not identical, latent subgroup structure. One reassuring aspect was that a subgroup with signs of nerve root involvement was identified by both approaches since this is considered to be a specific LBP subgroup [49–51].

A very high certainty of subgroup allocation was observed in both approaches, with the *single-stage LCA* displaying slightly higher posterior probabilities. This could be a natural consequence of the types of included variables in the *single-stage LCA*, as it included categorical, continuous and ordinal variables, whereas the *two-stage LCA* only included categorical variables that provide less information for the analysis.

A higher number of subgroups were preferred than would have been the result of a selection procedure guided solely by statistical measures. We chose these more complex models because they revealed some distinct subgroup differences that potentially could be of clinical importance. Based purely on our statistical criteria, a solution with two patient subgroups would be chosen from both the *single-stage* and the *two-stage LCA* that roughly describes only two levels of LBP severity. Previous LCA studies of musculoskeletal pain populations including people with LBP [22–25] have identified smaller numbers of subgroups than in our preferred solutions. This is likely to be partly due to these studies basing their subgrouping on less information, for example, only on pain location [23, 24] or psychological factors [22], and partly due to their model selection being determined only by measures of statistical fit.

In contrast to a previous study comparing the *single-stage* and the *two-stage* approach in people with chest pain [26], our study did not show increased clinical interpretability for the *two-stage LCA*. This could be due to increased model complexity in our study, as some health domains had more variables (more dimensions) in this LBP sample and therefore the *first stage* of the *two-stage LCA* resulted in conceptually more complex domain-specific patient categorisations. Entering complex domain-specific patient categorisations into the second stage LCA made the interpretation of the final patient subgroups more challenging, especially because each patient subgroup typically consisted of a mixture of participants from more than one domain-specific patient category. Another study on less complex data involved a *two-stage LCA, which* described financial poverty, and resulted in interpretable and meaningful subgroup solutions in the second stage of LCA [52]. However, their results were not compared with that of a *single-stage* approach.

Ideally, a *single-stage LCA* would be performed to avoid missing inter-domain relationships and it would include a limited number of variables that most strongly inform subgroup formation. However, as there is an absence of a coherent theoretical model for LBP and no a priori knowledge to inform the choice of optimal variables representing all aspects of potential importance for LBP subgroup formation, this would be premature in LBP. Therefore, it is currently necessary to include a high number of variables to represent LBP and potentially this increases the risk of some dimensions or domains being allocated too much weight in the modelling process. The *two-stage* approach was one way of addressing this issue by exploring the existence of potentially important aspects within each of the health domains to be used in a subsequent analysis. Our results indicate that either the features identified within health domains were not distinct characteristics of patient subgroups or that information about these features was lost from the first to the second stage LCA due to limitations of the method. Our approach of using the subgroup membership from the first stage in the second stage LCA meant that information was lost concerning (i) the certainty of subgroup membership and (ii) subgroup scores on original (baseline) variables. It would be useful to further investigate the possibility of better approaches for utilising the information of the first stage in the second stage LCA.

Both the single-stage and the two-stage LCA had methodological advantages and disadvantages and some have been described above. Further to this, when using the two-stage LCA there is a risk of classifying variables with a strong inter-relationship into different domains, whereas this limitation is avoided using the single-stage approach. However, by using the two-stage approach we attempt to guide LCA by using a potentially more correct grouping of variables which represent similar dimensions or domains of LBP. In this way, the two-stage approach is less objective than the single-stage approach, but allows incorporating prior knowledge of health domains by having all dimensions equally represented, which may or may not better represent the potential contribution of domain-specific information.

Another methodological issue is that the information based on the distribution or scores of the single variables is lost when using the two-stage approach which potentially could result in spurious subgroupings, as this information could be of importance, especially when using a qualitative approach. However, the consequence of this methodological aspect is unknown and could be further investigated.

Based on the above considerations and the results of the current explorative study, we believe that it is premature to reach a definite conclusion about which LCA approach to recommend. However, the intention of simplifying the LCA subgrouping method by using a two-stage approach did not succeed when using our data.

### Strengths and limitations

Our study included a relatively large sample with a wide range of LBP conditions. This diversity resulted from the recruitment of patients reporting LBP with and without leg pain and only excluded those who were pregnant or where a serious pathology of the back was suspected. Also, the analyses were based on a comprehensive data collection that, in addition to patient-reported data, included the findings from a standardised clinical examination [29].

We regard the qualitative model selection procedure to be a further strength, as the aim was to explore ways to investigate associations between the biopsychosocial aspects of LBP patients that potentially could improve the understanding of the LBP condition. Our aim was not to identify a subgroup solution to be implemented in clinical practice, which would have required more emphasis on the likely reproducibility of the models and therefore on using model fit measures for model selection. Our statistical criterion, the 1% decrease of BIC, would have then suggested a two-subgroup solution in both approaches, whereas if the statistical criterion were ‘lowest BIC’, then a five-subgroup solution would have been selected for the *single-stage LCA* and a two-subgroup solution selected for the *two-stage LCA.* The preferred larger models have more distinct and interesting characteristics that we believe warrant further investigation to determine their potential clinical importance. Especially, as one of the advantages of LCA is that it identifies relationships among baseline variables which might be strong for some subgroups but weak or absent in others, and these specific combinations of characteristics might not relate to outcomes in a similar way for all subgroups. However, we do acknowledge that from the clinical perspective, some subgroups appear rather similar and in the long run it might be relevant to collapse these, however, we do believe that their association with outcomes should be explored first.

Regarding generalisability, a limitation of the study was that the many theoretical and methodological decisions that needed to be made during the process might have influenced the results. Such decisions include: the choices during the pre-processing of the available data, the health domains chosen to be included, the variables used within each health domain, the way in which different criteria were managed within the model selection process, the interpretation of the identified subgroups, and the evaluation of each LCA approach.

As we included many variables, two aspects should be addressed. Firstly, some of the included variables may not have added information to the subgroup solutions and, therefore, were only adding noise to the analysis. It is not clear from simulation studies if inclusion of noise variables in LCA negatively affects the subgroup identification [33, 53]. However, in one study, the addition of noise variables did not seem to change the identified subgroups, albeit that the latent data structures modelled were quite simple [17]. Therefore, any consequences for our study are unknown. Secondly, many conceptually related items were included and some variables remained correlated within the resulting subgroups, which is not concordant with an underlying assumption of LCA. As pointed out by Suppes and Zanotti [54] and Swanson [53], violating the local independence assumption most often leads to an increased number of subgroups when using statistical criteria. However, as we preferred subgroup solutions larger than what would have been selected by statistical criteria, this consideration is likely to have been of minor concern.

### Where to from here?

This study identified two LCA models for subgrouping patients with LBP. However, the extent to which these subgroups would be identified in other patient cohorts was not investigated, nor if the subgroups have any potential clinical importance (prognostic or treatment selection value). Aspects relating to the identified subgroups that warrant further investigation include the subgroups’ association with longitudinal outcome measures such as pain intensity, disability or pain trajectories. In addition, future studies of the validity of the subgroups may make it possible to judge whether one subgroup solution is preferable to another. Also, to determine if the more complex solutions obtained by adding more information usefully add to the understanding of LBP, the preferred models could be compared to simpler models identified within this data set or compared to other subgrouping methods, such as the SBT, using longitudinal patient outcomes. Furthermore, of interest would be a study comparing the value of the information available after the first stage of the *two-stage* approach with the value of the information available after the second stage.

From a statistical perspective, this study suggests that it is not impossible to include 112 variables in an LCA model. However, the single-stage approach did not identify as many distinct features of the subgroups as found within the first stage of the two-stage LCA (domain-specific patient categorisations). Reasons for this could be that only a few distinct characteristics existed or that the large numbers of variables being modelled increased the complexity to the point at which the distinctiveness of these scoring patterns was muted. Therefore, we do believe that reducing the number of variables in advance would be an advantage in future studies.

## Conclusion

Using LCA to identify LBP subgroups, both a *single-stage LCA* and a *two-stage LCA* approach identified clinically interpretable patient subgroups with quite substantial overlap between the models from both approaches. Contrary to our expectations, the two-stage LCA approach did not increase the interpretability of the patient subgroups when compared to the single-stage LCA. Further analysis of the identified patient subgroups’ potential association with patient outcomes may help to inform the selection of a preferred LCA approach. Furthermore, testing of the *single-stage* and *two-stage LCA* approaches in other datasets would also provide useful insights into the extent to which the usefulness of these approaches is dataset-specific.

## Additional files

Additional file 1: Overview of variables used in this study, classified by health domains. Documentation of data management. The variables were used in the single stage LCA (all at once) and in the first stage of the two-stage LCA (by health domain). (PDF 267 kb)
Additional file 2: Single-stage patient subgroups based on variables from the activity domain (a), the contextual factors domain (b), the pain domain (c), the participation domain (d), the physical impairment domain (e), and the psychology domain. (PDF 1.50 mb)
Additional file 3: Descriptive presentation and profile plots of the preferred model solution for the second stage of the two-stage approach and for each of the domain-specific patient categorisations (result of the first stage of the two-stage LCA). Presentation of the standardised wording used in the descriptive process and tables for each of the preferred LCA models with descriptive presentation of each subgroup/category and associated profile plot. (PDF 10.4 mb)
Additional file 4: Stacked bar chart for each two-stage patient subgroup based on the conditional probabilities of each domain-specific patient category (the identified latent variables from the first stage Latent Class Analysis) from the activity domain (a), the contextual factors domain (b), the pain domain (c), the participation domain (d), the physical impairment domain (e), and the psychology domain, respectively. (PDF 0.98 mb)
Additional file 5: Statistical characteristics for identified patient subgroup models. One table with statistical characteristics for each LCA approach. (PDF 115 kb)
Additional file 6: Components included in the consensus process to select a preferred model for the single-stage LCA approach. Model summary output from the Latent Class Analysis for all the derived models and for each of these: presentation of included variables, subgroup size, conditional probabilities and means for each subgroup, loadings and profile plots. In addition, a presentation of all profile plots at once, including a brief clinical description of the subgroups and furthermore, a more thorough preliminary description of the preferred model with seven subgroups. (XLSX 828 kb)
Additional file 7: Components included in the consensus process to select a preferred model for the two-stage LCA approach. Model summary output from the Latent Class Analysis for all the derived models and for each of these: presentation of included variables, subgroup size, conditional probabilities and means for each subgroup and loadings. In addition, a presentation of the profile plots including a brief clinical description of the subgroups. (XLSX 195 kb)
Additional file 8: Descriptive presentation of the patient subgroups identified in the single-stage LCA. Presentation of the standardised wording used in the descriptive process and detailed statistical characteristics of each of the patient subgroups. In addition, descriptive presentation of each of the patient subgroups divided into health domain to ease the interpretation. (PDF 442 kb)

## Abbreviations

AROM: Active range of motion
BIC: Bayesian information criterion
BMI: Body mass index
Ext: Extension
FABQ: Fear-avoidance beliefs questionnaire
Flex: Flexion
ICF: International classification of functioning, disability and health
LBP: Low back pain
LCA: Latent class analysis
MDI: Major depression inventory
RMDQ: Roland-morris disability questionnaire
SBT: STarT back tool
SI: Sacroiliac
SPL: Social participation limitations
SS: Single-stage patient subgroup
TrP: Trigger points
TS: Two-stage patient subgroup
WHO: World health organization
