# Supplementary material for: Identifying subgroups of patients using latent class analysis: should we use a single-stage or a two-stage approach? A methodological study using a cohort of patients with low back pain
Source: BMC Musculoskelet Disord. 2017 Feb 1;18:57. doi: 10.1186/s12891-017-1411-x (PMC5286735; doi:10.1186/s12891-017-1411-x)
Supplement: Additional file 1: — Overview of variables used in this study, classified by health domains. Documentation of data management. The variables were used in the single stage LCA (all at once) and in the first stage of the two-stage LCA (by health domain). (PDF 267 kb) [file 12891_2017_1411_MOESM1_ESM.pdf]

Additional file 1

Overview of variables used in the Latent Class Analysis, classified by health domains

The variables were used in the single stage LCA (all at once) and in the first stage of the two-stage LCA (by health domain)

Table 1. Activity domain

Abbreviated version of variable	Variable	Response options (range)	Categorisation or reversing of variables (if performed)	Data type	References	Missing observations, N (%)
Only walked short distances	I have only walked short distances because of my back pain (SBT-3)	0=disagree<br>1=agree		Dichotomous	Validity [1-4]	29 (3.1)
Dressed more slowly last two weeks	In the last 2 weeks, I have dressed more slowly than usual because of back pain (SBT-4)	0=disagree<br>1=agree		Dichotomous		16 (1.7)
Change position frequently	I change position frequently to try to get my back or leg comfortable (RMDQ-2)	0=no<br>1=yes		Dichotomous	Reliability and validity [5, 6]	16 (1.7)
Walk more slowly	I walk more slowly than usual because of my back problem or leg pain (sciatica) (RMDQ-3)	0=no<br>1=yes		Dichotomous		20 (2.2)
Not doing usual jobs around the house	Because of my back problem, I am not doing any of the jobs that I usually do around the house (RMDQ-4)	0=no<br>1=yes		Dichotomous		18 (1.9)
Use handrail to get upstairs	Because of my back problem, I use a handrail to get upstairs (RMDQ-5)	0=no<br>1=yes		Dichotomous		18 (1.9)
Hold on to something to get out of an easy chair	Because of my back problem, I have to hold on to something to get out of an easy chair (RMDQ-6)	0=no<br>1=yes		Dichotomous		16 (1.7)

Get dressed more slowly	I get dressed more slowly than usual because of my back problem or leg pain (sciatica) (RMDQ-7)	0=no<br>1=yes		Dichotomous		15 (1.6)
Only stand for short periods of time	I only stand for short periods of time because of my back problem or leg pain (sciatica) (RMDQ-8)	0=no<br>1=yes		Dichotomous		15 (1.6)
Try not to bend or kneel down	Because of my back problem, I try not to bend or kneel down (RMDQ-9)	0=no<br>1=yes		Dichotomous		17 (1.8)
Difficult to get out of a chair	I find it difficult to get out of a chair because of my back problem or leg pain (sciatica) (RMDQ-10)	0=no<br>1=yes		Dichotomous		14 (1.5)
Difficult to turn over in bed	I find it difficult to turn over in bed because of my back problem or leg pain (sciatica) (RMDQ-12)	0=no<br>1=yes		Dichotomous		18 (1.9)
Trouble putting on socks	I have trouble putting on my socks (or stockings) because of the pain in my back or leg (RMDQ-13)	0=no<br>1=yes		Dichotomous		16 (1.7)
Only walk short distances	I only walk short distances because of my back problem or leg pain (sciatica) (RMDQ-14)	0=no<br>1=yes		Dichotomous		16 (1.7)
Avoid heavy jobs around the house	I avoid heavy jobs around the house because of my back problem (RMDQ-16)	0=no<br>1=yes		Dichotomous		14 (1.5)
Go upstairs more slowly	Because of my back problem, I go upstairs more slowly than usual (RMDQ-18)	0=no<br>1=yes		Dichotomous		15 (1.6)
Stay in bed most of the time	I stay in bed most of the time because of my back or leg pain (sciatica) (RMDQ-19)	0=no<br>1=yes		Dichotomous		14 (1.5)
Do less daily work around the house	Because of my back problem, I am doing less of the daily work around the house than I would usually	0=no<br>1=yes		Dichotomous		15 (1.6)

	do (RMDQ-22)					
Cannot do physical activities which (might) make pain worse	I cannot do physical activities which (might) make my pain worse (FABQ-5)	0=completely disagree<br>3=unsure<br>6=completely agree		Ordinal	Validity and reliability [7-9]	34 (3.7)
Cannot work with present pain	I cannot do my normal work with my present pain (FABQ-13)	0=disagree<br>1=unsure<br>2=agree	*Original scale:*<br>0=completely disagree<br>3=unsure<br>6=completely agree<br>*Comment:*<br>Trichotomised before the LCA due to highly skewed data distribution (few observations answering other than 'disagree')	Ordinal		41*(4.8)
Best posture to sit	Best posture is to sit#	0=no<br>1=yes	*Original scale:*<br>As shown. The variable was part of a test battery for ‘facet joint syndrome’. Only to be asked if patients had dominating back pain.	Dichotomous	Element of diagnostic classification [10, 11]	105** (11.5)
Best activity is not to walk	Best activity is *not* to walk#	0=no<br>1=yes	*Original scale:*<br>Best activity to walk, 1=yes<br>Part of a test battery for ‘facet joint syndrome’. Only to be asked if patients had dominating back pain.<br>For the LCA, variable was used in its original form<br>*Comment:*<br>For the descriptive analysis and profile plots, the variable was reverse scored so a high score indicated a higher risk of a poor prognosis cf. the analysis (profile plots) showed, that patients seem to be worse off if best activity was *not* to walk	Dichotomous		119**<br>(13.0)

LCA = Latent Class Analysis
FABQ = Fear-Avoidance Beliefs Questionnaire
RMDQ = Roland-Morris Disability Questionnaire (23-item)
SBT = STarT Back Tool
*The questions were only to be answered if the patient was working. Calculation of missing data on those variables excludes: students, unemployed, early retirement/retirement and health-related retirement
** The questions were only to be answered if there was dominating back pain and the amount of missing data reported relative to that
#Variables from the clinician-reported questionnaire. All other variables are from the patient self-reported baseline questionnaire

Table 2. Contextual factors domain

Abbreviated version of variable	Variable	Response options (range)	Categorisation or reversing of variables (if performed)	Type	References	Missing observations, N (%)
Health insurance	Do you have a private or work-related health insurance (0=no, 1=yes)	0=no<br>1=yes	*Comment:*<br>Reverse scored in the profile plots to make a high score indicate a higher risk of a poor prognosis	Dichotomous	None<br>(own production)	176 (19.0)
Smoking status	Smoking status (1=smoker, 2=ex-smoker,3=non-smoker)	1=smoker<br>2=ex-smoker<br>3=non-smoker	*Comment:*<br>Reverse scored in the profile plots to make a high score indicate a higher risk of a poor prognosis:<br>1=non-smoker<br>2=ex-smoker<br>3=smoker	Ordinal	Validity<br>[12]	21 (2.3)
Claim for compensation	I have a claim for compensation for my pain (0=disagree,1= don't know, 2=agree) (FABQ-8)	0=disagree<br>1= unsure<br>2=agree	*Original scale:*<br>0=completely disagree<br>3=unsure<br>6=completely agree<br>*Comment:*<br>Trichotomised before the LCA due to the highly skewed data distribution (few participants answering other than 'disagree')	Ordinal	Validity and reliability [7-9]	26 (2.8)
Age	Age	18-66	*Original scale:*<br>Birthdate and year<br>*Comment:*<br>Any age between 18 and 65 years was accepted (inclusion criterion).<br>All ages were calculated on a specific date and therefore some observations appear to be younger than 18 years of age and some older than 65 years of age.	Continuous	None<br>(own production)	0 (0.0)
Sex	Sex (1=male)	0=female<br>1=male		Dichotomous	None<br>(own production)	0 (0.0)
Educational level	Highest educational level	1=no qualification<br>2=vocational training<br>3=higher education <3 years<br>4=higher education 3-4 years<br>5=higher education >4 years	*Comment:*<br>Reverse scored in the profile plots to make a high score indicate a higher risk of a poor prognosis:<br>1=higher education >4 years<br>2=higher education 3-4 years<br>3=higher education <3 years<br>4=vocational training<br>5=no qualification	Ordinal	None<br>(own production)	22 (2.4)

Work situation	Current work situation	1=self-employed<br>2=full-time (37 hours per week)<br>3=part-time (<37 hours per week)<br>4=student<br>5=unemployed<br>6=early retirement / retired<br>7=health-related retirement<br>8=other	*Original scale:*<br>1=self-employed<br>2=co-working partner<br>3=full-time (37 hours per week)<br>4=part-time (<37 hours per week)<br>5=student<br>6=stay-at-home<br>7=unemployed<br>8=early retirement / retired<br>9=health-related retirement<br>10=other<br>*Comment:*<br>'Co-working' and 'stay-at-home' were pooled with 'other' before the LCA due to highly skewed data distribution	Multistate nominal	None (own production)	20 (2.2)
Height	Height	153-201	*Original scale:*<br>Patient self-reported in cm (no predefined range)	Continuous	Validity [13]	20 (2.2)
BMI	Body Mass Index (BMI)	18-59	*Original scale:*<br>Patient self-reported height (cm) and weight (kg) and BMI was calculated based on the responses as BMI = weight (kg) / height (m)^2<br>No predefined range.	Continuous	[14]	25 (2.7)
Self-reported general health	Self-reported general health (EQ-5D)	0=worst imaginable health state<br>100=best imaginable health state	*Comment:*<br>Reverse scored in the profile plots to make a high score indicate a higher risk of a poor prognosis:<br>0=best imaginable health state<br>100=worst imaginable health state	Continuous	Validity [15]	33 (3.6)
No chronic disease	Presence of a chronic disease: No chronic disease[#]	0=no<br>1=yes	*Original scale:*<br>The chiropractors asked the patients about comorbidity and could tick off:<br>No chronic diseases<br>OR<br>any of the following categories:<br>• Heart/circulation disease<br>• Lung disease<br>• Asthma/allergy<br>• Gastro-intestinal disease<br>• Gynaecological/obstetrical disease<br>• Neurological disease<br>• Depression or other psychological disease	Dichotomous	None (own production)	0 (0.0)
Heart disease	Presence of a chronic disease: Heart disease[#]	0=no<br>1=yes		Dichotomous		0 (0.0)

Asthma/allergy	Presence of a chronic disease: Asthma/allergy#	0=no<br>1=yes	• Musculoskeletal disease (other than the low back)<br>• Chronic pain condition (fibromyalgia, migraine or the like)<br>• Diabetes<br>• Other chronic disease<br>• Unsure<br>In addition:<br>Current or previous cancer disease (yes/no)<br>*Comment:*<br>Due to highly skewed data distribution (<3% responses) the following variables were pooled with the variable 'other chronic diseases' before the LCA:<br>• Lung disease<br>• Gastro-intestinal disease<br>• Gynaecological/obstetrical disease<br>• Neurological disease<br>• Chronic pain condition (fibromyalgia, migraine or the like)<br>• Diabetes<br>• Cancer (current or previous)<br>The 'unsure' responses were recoded as missing (<1%)<br>Six independent variables represent comorbidity<br><br>*Specific comment to 'No chronic disease':*<br>Reverse-scored in the profile plots to make a high score indicate a higher risk of a poor prognosis	Dichotomous		0 (0.0)
Depression/other psychological disease	Presence of a chronic disease: Depression or another psychological disease#	0=no<br>1=yes		Dichotomous		0 (0.0)
Musculoskeletal disorder	Presence of a chronic disease: Musculoskeletal disease apart from the low back#	0=no<br>1=yes		Dichotomous		0 (0.0)
Another chronic disease	Presence of a chronic disease: Another chronic disease #	0=no<br>1=yes		Dichotomous		0 (0.0)

LCA = Latent Class Analysis
#Variables from the clinician-reported physical examination. All other variables are from the patient-reported baseline questionnaire

Table 3. Pain domain

Abbreviated version of variable	Variable	Response options (range)	Categorisation or reversing of variables (if performed)	Data type	References	Missing observations, N (%)

Duration of LBP	For how long has this episode of LBP lasted?	1=0-2 weeks<br>2=2-4 weeks<br>3=1-3 months<br>4=>3 months		Ordinal	None<br>(own production)	18 (1.9)
LBP intensity	Back pain intensity on average the last week (Numeric Pain Rating Scale)	0=no pain<br>10=worst imaginable pain		Continuous	Validity and reliability<br>[16, 17]	25 (2.7)
Leg pain intensity	Leg pain intensity on average the last week (Numeric Pain Rating Scale)	0=no pain<br>1=mild pain<br>2=moderate-worst imaginable pain	*Original scale:*<br>0=no pain<br>10=worst imaginable pain<br>*Comment:*<br>Trichotomised before the LCA due to highly skewed data distribution:<br>0=no pain<br>1=1-4 mild pain<br>2=>4 moderate - worst imaginable pain	Ordinal	Validity and reliability<br>[16, 17]<br><br>Trichotomisation:<br>[18, 19]	43 (4.6)
Previous LBP episodes	How many episodes of LBP have you had before this one?	0=no previous episodes<br>1=1-3 previous episodes<br>2=more than 3 previous episodes		Ordinal	None<br>(own production)	19 (2.0)
More than 30 days of LBP last year	How many days have you had LBP within the last year?	0=≤30 days<br>1=>30 days		Dichotomous	None<br>(own production)	32 (3.4)
Pain has spread down leg(s)	My back pain has spread down my leg(s) at some time in the last 2 weeks (SBT-1)	0=no<br>1=yes		Dichotomous	Validity<br>[1-4]	16 (1.7)
Shoulder/neck pain	I have had pain in the shoulder or neck at some time in the last 2 weeks (SBT-2)	0=no<br>1=yes		Dichotomous		20 (2.2)

Very-extremely bothersome BP	Overall, how bothersome has your back pain been in the last 2 weeks? (SBT-9)	0=no-moderately bothersome pain<br>1=very-extremely bothersome pain	*Original scale:*<br>0=not at all<br>0=slightly<br>0=moderately<br>1=very much<br>1=extremely<br>*Comment:*<br>Dichotomised before the LCA as prescribed in the scoring instruction (0 and 1)	Dichotomous		12 (1.3)
Back/leg painful almost all the time	My back or leg is painful almost all the time (RMDQ-11)	0=no<br>1=yes		Dichotomous	Reliability and validity [5, 6]	19 (2.0)
Pain caused by physical activity	My pain was caused by physical activity (FABQ-1)	0=completely disagree<br>3=unsure<br>6=completely agree		Ordinal	Validity and reliability [7-9]	18 (1.9)
Physical activity makes worse	Physical activity makes my pain worse (FABQ-2)	0=completely disagree<br>3=unsure<br>6=completely agree		Ordinal		23 (2.5)
Pain distribution	Pain distribution[#]	0=back pain only<br>1=back pain and pain in one leg<br>2=back pain and pain in both legs<br>3=leg pain only	*Original scale:*<br>Variable created before the LCA based on the following three yes/no questions:<br>• Back pain<br>• Leg pain right<br>• Leg pain left<br>Participants who did not report any pain, were recoded as missing data<br>*Comment:*<br>For the profile plots, the category 'back pain only' was reverse scored so a high score indicated a higher risk of a poor prognosis (*not* only low back pain)	Multistate nominal	None<br>(own production)	15 (1.6)
LBP not dominating	Back pain is not dominating?[#]	0=no<br>1=yes	*Original scale:*<br>Variable created before the LCA was 'dominating back pain' based on the following three yes/no questions:<br>1=dominating back pain (above gluteal fold)<br>0=dominating leg pain (below gluteal fold)<br>0=back pain equals leg pain<br>*Comment:*<br>Two-stage LCA: For the descriptive analysis and profile	Dichotomous	None<br>(own production)	9 (1.0)

			plots, the variable was reverse scored so a high score indicated a higher risk of a poor prognosis (*not* having dominating back pain = higher risk)<br>Single stage LCA: The variable was reverse scored before the LCA			
No paraspinal pain onset	No paraspinal pain onset[#]	0=no<br>1=yes	*Original scale:*<br>Paraspinal pain onset, 1=yes<br>Part of a test battery for 'facet joint syndrome'. Only to be asked if patients had dominating back pain.<br>For the LCA, the variable was used in its original form<br>*Comment:*<br>Two-stage LCA: For the descriptive analysis and profile plots, the variable was reverse scored so a high score indicated a higher risk of a poor prognosis cf. the analysis (profile plots) showed, that patients seem to be worse off if *not* having paraspinal pain onset<br>Single stage LCA: The variable was reverse scored before the LCA	Dichotomous	Element of diagnostic classification [10, 11]	121* (13.2)

LCA = Latent Class Analysis
LBP = Low back pain
FABQ = Fear-Avoidance Beliefs Questionnaire
RMDQ = Roland-Morris Disability Questionnaire (23-item)
SBT = STarT Back Tool
* The question was only to be answered if there was dominating back pain and the amount of missing data reported relative to that
[#]Variables from the clinician-reported questionnaire. All other variables are from the patient self-reported baseline questionnaire

Table 4. Participation domain

Abbreviated version of variable	Variable	Response options (range)	Categorisation or reversing of variables (if performed)	Data type	References	Missing observations, N (%)
Physical workload	Physical load at work	1=sitting<br>2=sitting and walking<br>3=light physical load<br>4=heavy physical load		Ordinal	[20]	49 (5.3)

Days with sick leave last month	Days with sick leave the last month	0=0 days<br>1=1-5 days (workweek)<br>2=6-31 days	*Original scale:*<br>0-31 days, continuous scale (patient's noted a number themselves)<br>*Comment:*<br>Trichotomised before the LCA to an ordinal scale due to highly skewed data distribution (few observations had any days of sick leave)	Ordinal	None<br>(own production)	46 (5.0)
Stay home most of the time	I stay at home most of the time because of my back problem or leg pain (sciatica) (RMDQ-1)	0=no<br>1=yes		Dichotomous	Reliability and validity [5, 6]	18 (1.9)
Decreased sexual activity	Because of my back problem, my sexual activity is decreased (RMDQ-20)	0=no<br>1=yes		Dichotomous		31 (3.3)
Pain caused by work/accident at work	My pain was caused by my work or by an accident at work<br>(FABQ-6)	0=disagree<br>1=unsure<br>2=agree	*Original scale:*<br>0=completely disagree<br>3=unsure<br>6=completely agree<br>*Comment:*<br>Trichotomised before the LCA due to the highly skewed data distribution (few participants answering other than 'disagree'):<br>0=disagree (0-2)<br>1=unsure (3)<br>2=agree (4-6)	Ordinal	Validity and reliability [7-9]	19* (2.1)
Work-aggravated pain	My work aggravated my pain (FABQ-7)	0=disagree<br>1=unsure<br>2=agree		Ordinal		19*(2.1)
Work is too heavy	My work is too heavy for me (FABQ-9)	0=disagree<br>1=unsure<br>2=agree		Ordinal		40* (4.7)
Work makes/would make pain worse	My work makes or would make my pain worse (FABQ-10)	0=disagree<br>1=unsure<br>2=agree		Ordinal		43*(5.1)

LCA = Latent Class Analysis
RMDQ = Roland-Morris Disability Questionnaire (23-item)
*The questions were only to be answered if the patient was working. Calculation of missing data on those variables excludes: students, unemployed, early retirement/retirement and health-related retirement

Table 5. Physical impairment domain (all items are from the clinician-reported physical examination)

Abbreviated version of variable	Variable	Response options (range)	Categorisation or reversing of variables (if performed)	Type	References	Missing observations, N (%)
Acute lateral shift	Observation: Acute lateral shift	0=no<br>1=yes		Dichotomous	Validity and reliability [21]	78 (8.4)
Acute flexion deformity	Observation: Acute flexion deformity	0=no<br>1=yes		Dichotomous		78 (8.4)
Pain on flexion	Pain on active range of motion (AROM) of the lumbar back in flexion	0=no pain<br>1=back pain<br>2=leg pain with or without back pain	*Original scale:*<br>0=no pain<br>OR<br>any of the following response options could be ticked:<br>• back pain replicated on movement<br>• leg pain replicated on movement<br>*Comment:*<br>Trichotomised before the LCA.	Multistate nominal		91 (9.8)
Pain on extension	Pain on AROM in extension	0=no pain<br>1=back pain<br>2=leg pain with or without back pain		Multistate nominal		104 (11.2)
Pain on right side glide	Pain on AROM in side glide to the right	0=no pain<br>1=back pain<br>2=leg pain with or without back pain		Multistate nominal		136 (14.7)
Pain on left side glide	Pain on AROM in side glide to the left	0=no pain<br>1=back pain<br>2=leg pain with or without back pain		Multistate nominal		141 (15.2)
Pain on right rotation	Pain on AROM in rotation to the right	0=no pain<br>1=back pain<br>2=leg pain with or without back pain		Multistate nominal		162 (17.5)
Pain on left rotation	Pain on AROM in rotation to the left	0=no pain<br>1=back pain<br>2=leg pain with or without back pain		Multistate nominal		163 (17.6)

Reducible disc syndrome	Diagnosis based on 'Mechanical Diagnosis and Therapy' (MDT) as described by McKenzie': Reducible disc syndrome	0=no<br>1=yes	*Original scale:*<br>• Reducible disc syndrome<br>• Partly reducible disc syndrome<br>• Irreducible disc syndrome<br>• Dysfunction syndrome (disc-related diagnosis)<br>• Non-mechanical (disc-related diagnosis) [a]<br>• Adherent nerve root [a]<br>• Postural syndrome [a]<br>*Comment:*<br>The diagnosis made by the clinician was based on the McKenzie Method protocol (examination algorithm)<br>[a] These variables had a highly skewed data distribution (<1% of patients had the condition) and were not included in the LCA.<br>Patients without a diagnosis are therefore counted as missing for each of the four MDT diagnosis items	Dichotomous	Validity and reliability [10, 11]<br><br>Modifications performed to Petersen's diagnostic classifications [22]	6 (0.6)
Partly reducible disc syndrome	Diagnosis based on MDT: Partly reducible disc syndrome	0=no<br>1=yes		Dichotomous		6 (0.6)
Irreducible disc syndrome	Diagnosis based on 'MDT': Irreducible disc syndrome	0=no<br>1=yes		Dichotomous		6 (0.6)
Dysfunction syndrome	Diagnosis based on 'MDT': Dysfunction syndrome	0=no<br>1=yes		Dichotomous		6 (0.6)
Indication of herniated disc, right side	Neurological examination: Indication of herniated disc, right side	0=no<br>1=yes	*Original scale:*<br>0=No pain or muscular tightness upon Straight Leg Raise (SLR) or Reversed Laségue<br>OR<br>any of the following variables could be ticked:<br>• Positive SLR: replication of radicular pain to the leg (below the buttocks)<br>• Positive crossed SLR: replication of radicular pain to the leg not being raised<br>• Positive Reversed Laségue: replication of radicular pain to the leg (front of the thigh)<br>*Comment:*<br>Only prescribed if the patient had leg pain<br>Because of highly skewed distribution, the categories were pooled into 'indication of herniated disc' yes/no, though keeping the side specification (right/left)	Dichotomous	Validity and reliability [11, 10]	9 (1.0)
Indication of herniated disc, left side	Neurological examination: Indication of herniated disc, left side	0=no<br>1=yes		Dichotomous		15 (1.6)
Affected muscular strength, lower extremities	Neurological examination: Affected muscular strength for the lower extremities	0=no<br>1=yes	*Original scale:*<br>0=Normal muscle strength right and / or left<br>OR<br>any of the following variables could be graded for either	Dichotomous		0 (0.0)

			the right or left side:<br>• Hip flexion (L2-3)<br>• Knee flexion (L5)<br>• Knee extension (L4)<br>• Dorsal flexion of ankle joint / walk on heels (L5)<br>• Plantar flexion of ankle joint / walk on toes (S1)<br>• Extension first toe (L5)<br>• Flexion first toe (S1)<br>• Extension other toes<br>• Flexion other toes<br>Grading: 5=normal strength, 4=movement against external resistance, but less than normal, 3=Movement against gravity, but not against added resistance, 2=Movement at the joint with gravity eliminated, 1=Trace of contraction, but no movement at the joint, 0=No contraction or muscle movement<br>*Comment:*<br>Due to highly skewed data distribution and no obvious differences in affected muscle group or side, the overall response options (normal muscular strength left and right) were pooled and reverse scored before the LCA:<br>Affected muscular strength: yes/no			
Affected sensibility, lower extremities	Neurological examination: Affected sensibility for the lower extremities	0=no<br>1=yes	*Original scale:*<br>Normal sensibility (sense of touch) right and/or left<br>OR<br>any of the following variables could be graded for either the right or left side:<br>• L2 dermatome<br>• L3 dermatome<br>• L4 dermatome<br>• L5 dermatome<br>• S2 dermatome<br>• Non-dermatomal<br>Grading: Normal, reduced or dysesthesia (for touch)<br>*Comment:*<br>Due to highly skewed data distribution and no obvious difference in affected dermatome or side, the overall response options (normal sensibility left / right) were pooled and reverse scored before the LCA:<br>Affected sensibility: yes/no	Dichotomous		0 (0.0)

Affected deep tendon reflexes, lower extremities	Neurological examination: Affected deep tendon reflexes for the lower extremities	0=no<br>1=yes	*Original scale:*<br>Normal deep tendon reflexes right and/or left<br>OR<br>any of the following variables could be graded for either the right or left side:<br>• Patella reflex (L4)<br>• Hamstring reflex L5<br>• Achilles reflex (S1)<br>Grading: Normal, reduced, absent or hyperreflexia<br>*Comment:*<br>Due to highly skewed data distribution, the overall response options (normal deep tendon reflexes left / right) were pooled and reverse scored before the LCA: Affected deep tendon reflexes: yes/no	Dichotomous		0 (0.0)
Separation, SI test	Test of sacroiliac joint: Separation	0=negative (normal)<br>1=positive (uni- or bilaterally)	*Original scale:*<br>For each right sided SI joint test: positive or negative.<br>For each left sided SI joint test: positive or negative.<br>Tests were considered positive if there was replication of pain around the SI joint<br>*Comment:*<br>Due to no obvious difference in side, the variables were pooled to a dichotomous variable (negative or positive test)	Multistate nominal	Validity and reliability [11, 10]	64 (6.9)
Dorsal glide, SI test	Test of sacroiliac joint: Dorsal glide/thigh thrust (P4)	0=negative (normal)<br>1=positive (uni- or bilaterally)		Multistate nominal		61 (6.6)
Gaenslens, SI test	Test of sacroiliac joint: Gaenslens	0=negative (normal)<br>1=positive(uni- or bilaterally)		Multistate nominal		85 (9.2)
Compression, SI test	Test of sacroiliac joint: Compression	0=negative (normal)<br>1=positive(uni- or bilaterally)		Multistate nominal		81 (8.7)
Sacral thrust, SI test	Test of sacroiliac joint: Sacral thrust	0=negative (normal)<br>1=positive (uni- or bilaterally)		Multistate nominal		71 (7.7)
Pain on extension/ rotation	Pain on combined extension/rotation of the low back	1=no<br>2=yes	*Original scale:*<br>Pain on combined extension/rotation: 1=yes<br>Part of a test battery for 'facet joint syndrome'. Only to be asked if patients had dominating back pain.<br>Variable was used in its original form	Dichotomous	Validity and reliability [10, 11]	70* (7.6)

Pain on muscle palpation	Replication of pain on muscle palpation	0=no<br>1=yes		Dichotomous	Validity and reliability<br>[11, 10]	182 (19.6)
Painful muscles groups	Painful muscle group(s)	1=back muscles inclusive m. iliopsoas<br>2=buttock and leg muscles<br>3=both back and leg muscles	*Original scale:*<br>Chiropractors noted which muscles were painful / had pain referred from trigger points<br>*Comment:*<br>This variable was created before the LCA based on the chiropractors' written notes.<br>Three categories were made:<br>1=back muscles inclusive of the Iliopsoas muscles<br>2=buttock and leg muscles<br>3=both back and leg muscles	Multistate nominal	None<br>(own production)	219** (32.9)
Trigger points	Replication of pain by trigger points	0=no<br>1=yes		Dichotomous	Validity and reliability<br>[11, 10]	347 (37.4)
AROM = Active Range Of Motion<br>LCA = Latent Class Analysis<br>MDT = Mechanical Diagnosis and Therapy (responses to repeated end-range movements)[23]<br>* This question was only to be answered if there was dominating back pain and the amount of missing data reported relative to that<br>**The amount of missing data reported excludes those with no 'painful muscle palpation' *and* no 'referred pain from trigger points'						

Table 6. Psychology domain

Abbreviated version of variable	Variable	Response options (range)	Categorisation or reversing of variables (if performed)	Data type	References	Missing observations, N (%)
Able to decrease pain	Based on all things you do to cope or deal with your pain, on an average day, how much are you able to decrease it?	0=Can decrease it completely<br>10=Can't decrease it at all	*Original scale:*<br>0=can't decrease it at all<br>10=can decrease it completely<br>Item from the Örebro Musculoskeletal Pain Screening Questionnaire.<br>*Comment:*<br>Two-stage LCA: reverse-scored in the descriptive analysis and the profile plots to make a high score indicate a higher risk of a poor prognosis<br>Single-stage LCA: reverse-scored before the LCA	Continuous	Reliability and validity of the full Örebro pain questionnaire<br>[24, 25]	26 (2.8)
Negative recovery belief	How likely do you think it is that you would be fully	0=Likely to recover	*Original scale:*<br>0=not at all likely	Dichotomous	Validity<br>(Expectations and	13 (1.4)

	recovered in 3 months?	1=Unsure-not at all likely	10=very likely<br>*Comment:*<br>Dichotomised before the LCA due to highly skewed data distribution. Reverse-scored in the descriptive analysis and the profile plots to make a high score indicate a higher risk of a poor prognosis:<br>0=8-10 Likely to recover<br>1=0-7 Unsure-not at all likely		association with outcome/ change over time) [26, 27]	
Feel socially isolated	To what extent do you feel socially isolated?	0=not at all isolated<br>1=little to quite isolated	*Original scale:*<br>0=not at all isolated<br>10=quite isolated<br>*Comment:*<br>Dichotomised before the LCA due to highly skewed data distribution:<br>0=0 Not at all isolated<br>1=1-10 Little-quite isolated	Dichotomous	Validity [28]	11 (1.2)
Treatment not essential	How strongly do you agree: Treatment is essential to decrease my pain?	0=completely agree<br>10=completely disagree	*Comment:*<br>For the descriptive analysis, the question was rephrased to: Treatment not essential	Continuous	None<br>(own production)	20 (2.2)
Not safe to be physically active	It's not really safe for a person with a condition like mine to be physically active (SBT-5)	0=disagree<br>1=agree		Dichotomous	Validity<br>[1-4]	28 (3.0)
Worrying thoughts a lot of the time	Worrying thoughts have been going through my mind a lot of the time (SBT-6)	0=disagree<br>1=agree		Dichotomous		24 (2.6)
Terrible back pain, will never get better	I feel that my back pain is terrible and it's never going to get any better (SBT-7)	0=disagree<br>1=agree		Dichotomous		17 (1.8)
Not enjoyed things used to enjoy	In general I have not enjoyed all the things I used to enjoy (SBT-8)	0=disagree<br>1=agree		Dichotomous		19 (2.0)
Sleep less well	I sleep less well because of my back problem (RMDQ-15)	0=no<br>1=yes		Dichotomous	Reliability and validity [5, 6]	22 (2.4)
More irritable with people than usual	Because of my back problem, I am more irritable and bad tempered with people than usual<br>(RMDQ-17)	0=no<br>1=yes		Dichotomous		17 (1.8)

Rubbing/holding areas that hurt/are uncomfortable	I keep rubbing or holding areas of my body that hurt or are uncomfortable (RMDQ-21)	0=no<br>1=yes		Dichotomous		15 (1.6)
Often express concern	I often express concern to other people over what might be happening to my health (RMDQ-23)	0=no<br>1=yes		Dichotomous		17 (1.8)
Physical activity might harm back	Physical activity might harm my back (FABQ-3)	0=completely disagree<br>3=unsure<br>6=completely agree		Ordinal	Validity and reliability [7-9]	29 (3.1)
Should not do physical activity which (might) make pain worse	I should not do physical activities which (might) make my pain worse (FABQ-4)	0=completely disagree<br>3=unsure<br>6=completely agree		Ordinal		30 (3.2)
Work might harm back	My work might harm my back (FABQ-11)	0=completely disagree<br>3=unsure<br>6=completely agree		Ordinal		44* (5.2)
Should not do normal work with present pain	I should not do my normal work with my present pain (FABQ-12)	0=completely disagree<br>3=unsure<br>6=completely agree		Ordinal		41* (4.8)
Cannot work till pain is treated	I cannot do my normal work till my pain is treated (FABQ-14)	0=completely disagree<br>3=unsure<br>6=completely agree		Ordinal		43* (5.1)
Felt low in spirits/sad	Have you felt low in spirits or sad?<br>(MDI-1)	0=at no time<br>1=some of the time<br>2=slightly-all the time	*Original scale:*<br>0=at no time<br>1=some of the time<br>2=slightly less than half the time<br>3=slightly more than half the time<br>4=most of the time<br>5=all the time	Ordinal	Depression rating scale (validity) [29]	7 (0.8)

Lost interest in daily activities	Have you lost interest in your daily activities?<br>(MDI-2)	0=at no time<br>1=some of the time<br>2=slightly-all the time	*Comment:*<br>Trichotomised before the LCA due to highly skewed data distribution	Ordinal		10 (1.1)
Felt lacking in energy and strength	Have you felt lacking in energy and strength?<br>(MDI-3)	0=at no time<br>1=some of the time<br>2=slightly less than half the time<br>3=slightly more than half the time<br>4=Most of the time<br>5=All the time		Ordinal		5 (0.5)
Felt less self-confident	Have you felt less self-confident?<br>(MDI-4)	0=at no time<br>1=some of the time<br>2=slightly-all the time	*Original scale:*<br>0=at no time<br>1=some of the time<br>2=slightly less than half the time<br>3=slightly more than half the time<br>4=most of the time<br>5=all the time<br>*Comment:*<br>Trichotomised before the LCA due to highly skewed data distribution	Ordinal		9 (1.0)
Had a bad conscience	Have you had a bad conscience or feelings of guilt?<br>(MDI-5)	0=at no time<br>1=some of the time<br>2=slightly-all the time		Ordinal		5 (0.5)
Had difficulty in concentrating	Have you had difficulty in concentrating, e.g. when reading the newspaper or watching television?<br>(MDI-7)	0=at no time<br>1=some of the time<br>2=slightly-all the time		Ordinal		6 (0.6)

Felt very restless/subdued/ slowed down	Have you felt very restless/subdued or slowed down?<br>(MDI-8)	0=at no time<br>1=some of the time<br>2=slightly-all the time		Ordinal		6 (0.6)
Had trouble sleeping at night	Have you had trouble sleeping at night?<br>(MDI-9)	0=at no time<br>1=some of the time<br>2=slightly less than half the time<br>3=slightly more than half the time<br>4=Most of the time<br>5=All the time		Ordinal		6 (0.6)
Have suffered from reduced/increased appetite	Have you suffered from reduced/increased appetite?<br>(MDI-10)	0=at no time<br>1=some of the time<br>2=slightly-all the time	*Original scale:*<br>0=at no time<br>1=some of the time<br>2=slightly less than half the time<br>3=slightly more than half the time<br>4=most of the time<br>5=all the time<br>*Comment:*<br>Trichotomised before the LCA due to highly skewed data distribution	Ordinal		6 (0.6)

LCA = Latent Class Analysis
FABQ = Fear-Avoidance Beliefs Questionnaire
MDI = Major Depression Inventory
RMDQ = Roland-Morris Disability Questionnaire (23-item)
SBT = STarT Back Tool
*The questions were only to be answered if the patient was working. Calculation of missing data on those variables excludes: students, unemployed, early retirement/retirement and health-related retirement

References

1. Hill JC, Dunn KM, Lewis M, Mullis R, Main CJ, Foster NE et al. **A primary care back pain screening tool: identifying patient subgroups for initial treatment**. Arthritis Rheum. 2008; **59**(5):632-41.
2. Hill JC, Dunn KM, Main CJ, Hay EM. **Subgrouping low back pain: A comparison of the STarT Back Tool with the Örebro Musculoskeletal Pain Screening Questionnaire**. Eur J Pain. 2010; **14**(1):83-9.
3. Hill JC, Whitehurst DG, Lewis M, Bryan S, Dunn KM, Foster NE et al. **Comparison of stratified primary care management for low back pain with current best practice (STarT Back): a randomised controlled trial**. Lancet. 2011; **378**(9802):1560-71.
4. Morso L, Albert H, Kent P, Manniche C, Hill J. **Translation and discriminative validation of the STarT Back Screening Tool into Danish**. Eur Spine J. 2011; **20**(12):2166-73.
5. Albert HB, Jensen AM, Dahl D, Rasmussen MN. **[Criteria validation of the Roland Morris questionnaire. A Danish translation of the international scale for the assessment of functional level in patients with low back pain and sciatica]**. Ugeskr Laeger. 2003; **165**(18):1875-80.
6. Patrick DL, Deyo RA, Atlas SJ, Singer DE, Chapin A, Keller RB. **Assessing health-related quality of life in patients with sciatica**. Spine (Phila Pa 1976). 1995; **20**(17):1899-908; discussion 909.
7. Waddell G, Newton M, Henderson I, Somerville D, Main CJ. **A Fear-Avoidance Beliefs Questionnaire (FABQ) and the role of fear-avoidance beliefs in chronic low back pain and disability**. Pain. 1993; **52**(2):157-68.
8. Swinkels-Meewisse EJ, Swinkels RA, Verbeek AL, Vlaeyen JW, Oostendorp RA. **Psychometric properties of the Tampa Scale for kinesiophobia and the fear-avoidance beliefs questionnaire in acute low back pain**. Man Ther. 2003; **8**(1):29-36.
9. Grotle M, Brox JI, Vollestad NK. **Reliability, validity and responsiveness of the fear-avoidance beliefs questionnaire: methodological aspects of the Norwegian version**. J Rehabil Med. 2006; **38**(6):346-53.
10. Petersen T, Olsen S, Laslett M, Thorsen H, Manniche C, Ekdahl C et al. **Inter-tester reliability of a new diagnostic classification system for patients with non-specific low back pain**. Aust J Physiother. 2004; **50**(2):85-94.
11. Petersen T, Laslett M, Thorsen H, Manniche C, Ekdahl C, Jacobsen S. **Diagnostic classification of non-specific low back pain. A new system integrating patho-anatomic and clinical categories**. Physiother Theory Pract. 2003; **19**(4):213-37.
12. Bender D, Haubruck P, Boxriker S, Korff S, Schmidmaier G, Moghaddam A. **Validity of subjective smoking status in orthopedic patients**. Ther Clin Risk Manag. 2015; **11**:1297-303.
13. Niedhammer I, Bugel I, Bonenfant S, Goldberg M, Leclerc A. **Validity of self-reported weight and height in the French GAZEL cohort**. Int J Obes Relat Metab Disord. 2000; **24**(9):1111-8.
14. World Health Organization. **Physical status: the use and interpretation of anthropometry. Report of a WHO expert committee**. WHO technical report series no. 854. Geneva, Switzerland: WHO; 1995.
15. Rabin R, de Charro F. **EQ-5D: a measure of health status from the EuroQol Group**. Ann Med. 2001; **33**(5):337-43.
16. Jensen MP, Turner JA, Romano JM, Fisher LD. **Comparative reliability and validity of chronic pain intensity measures**. Pain. 1999; **83**(2):157-62.
17. Bolton JE, Wilkinson RC. **Responsiveness of pain scales: a comparison of three pain intensity measures in chiropractic patients**. J Manipulative Physiol Ther. 1998; **21**(1):1-7.
18. Dunn KM, Jordan K, Croft PR. **Characterizing the course of low back pain: a latent class analysis**. Am J Epidemiol. 2006; **163**(8):754-61.
19. Palos GR, Mendoza TR, Mobley GM, Cantor SB, Cleeland CS. **Asking the community about cutpoints used to describe mild, moderate, and severe pain**. J Pain. 2006; **7**(1):49-56.
20. Saltin B, Grimby G. **Physiological analysis of middle-aged and old former athletes. Comparison with still active athletes of the same ages**. Circulation. 1968; **38**(6):1104-15.

21. Triano JJ, Budgell B, Bagnulo A, Roffey B, Bergmann T, Cooperstein R et al. **Review of methods used by chiropractors to determine the site for applying manipulation**. Chiropr Man Therap. 2013; **21**(1):36.
22. Eirikstoft H, Kongsted A. **Patient characteristics in low back pain subgroups based on an existing classification system. A descriptive cohort study in chiropractic practice**. Man Ther. 2014; **19**(1):65-71.
23. McKenzie R, May S. **Lumbar Spine, Mechanical Diagnosis and Therapy**. 2nd ed. Waikanae, New Zealand: Spinal Publications Ltd; 2003.
24. Linton SJ, Boersma K. **Early identification of patients at risk of developing a persistent back problem: the predictive validity of the Orebro Musculoskeletal Pain Questionnaire**. Clin J Pain. 2003; **19**(2):80-6.
25. Grotle M, Vollestad NK, Brox JI. **Screening for yellow flags in first-time acute low back pain: reliability and validity of a Norwegian version of the Acute Low Back Pain Screening Questionnaire**. Clin J Pain. 2006; **22**(5):458-67.
26. Kamper SJ, Kongsted A, Haanstra TM, Hestbaek L. **Do recovery expectations change over time?** Eur Spine J. 2015; **24**(2):218-26.
27. Kongsted A, Vach W, Axo M, Bech RN, Hestbaek L. **Expectation of recovery from low back pain: a longitudinal cohort study investigating patient characteristics related to expectations and the association between expectations and 3-month outcome**. Spine (Phila Pa 1976). 2014; **39**(1):81-90.
28. Kent P, Mirkhil S, Keating J, Buchbinder R, Manniche C, Albert HB. **The concurrent validity of brief screening questions for anxiety, depression, social isolation, catastrophization, and fear of movement in people with low back pain**. Clin J Pain. 2014; **30**(6):479-89.
29. Olsen LR, Jensen DV, Noerholm V, Martiny K, Bech P. **The internal and external validity of the Major Depression Inventory in measuring severity of depressive states**. Psychol Med. 2003; **33**(2):351-6.
