# Supplementary figures and images for: Identifying subgroups of patients using latent class analysis: should we use a single-stage or a two-stage approach? A methodological study using a cohort of patients with low back pain
Source: BMC Musculoskelet Disord. 2017 Feb 1;18:57. doi: 10.1186/s12891-017-1411-x (PMC5286735; doi:10.1186/s12891-017-1411-x)

a

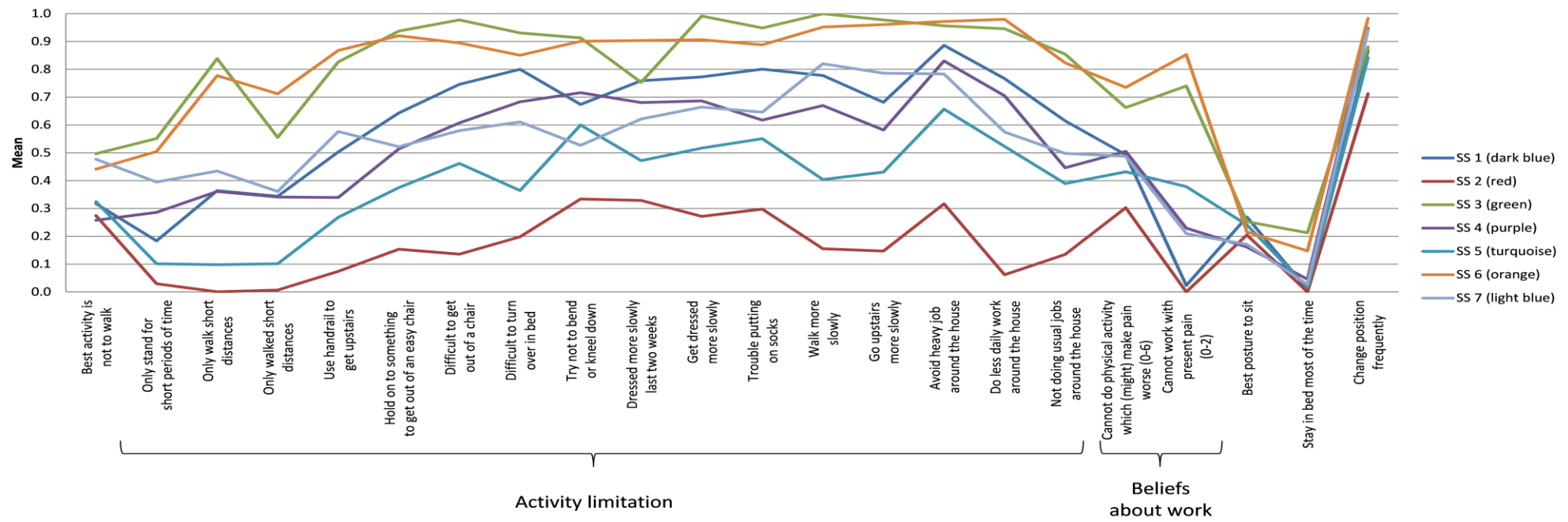

b

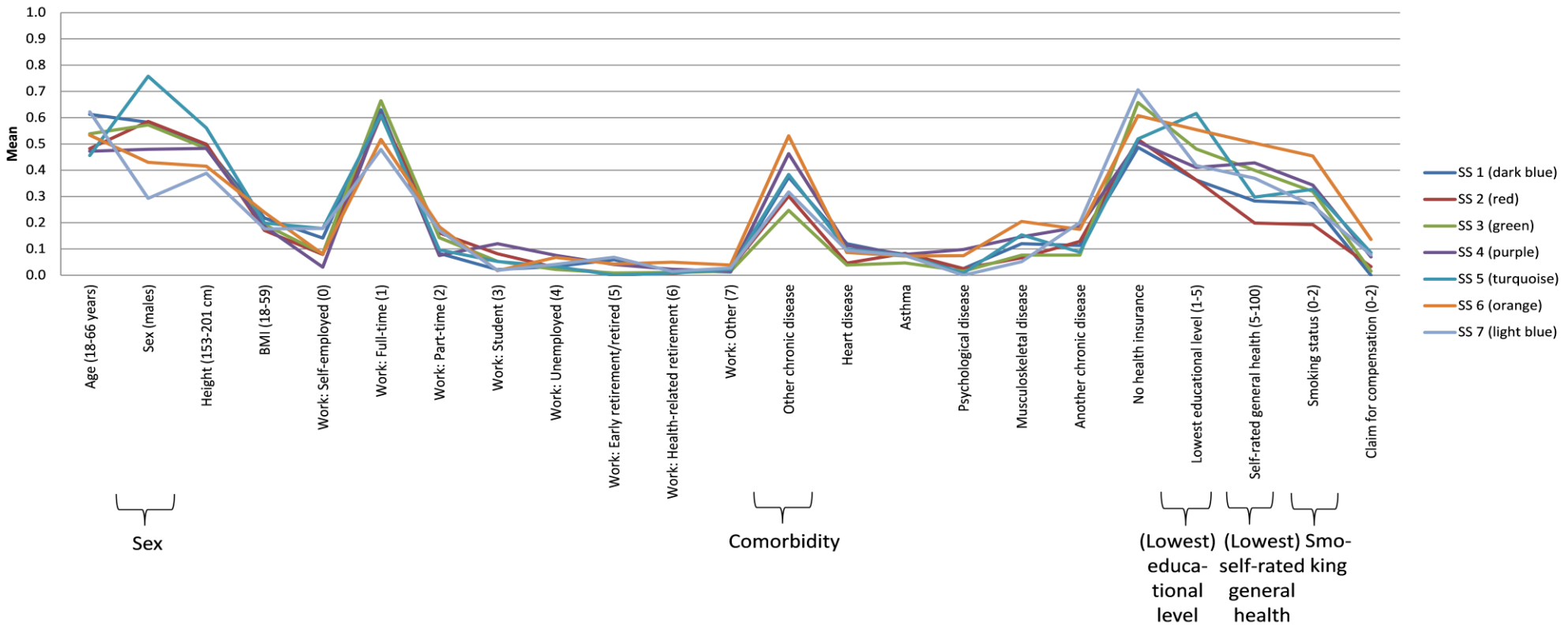

c

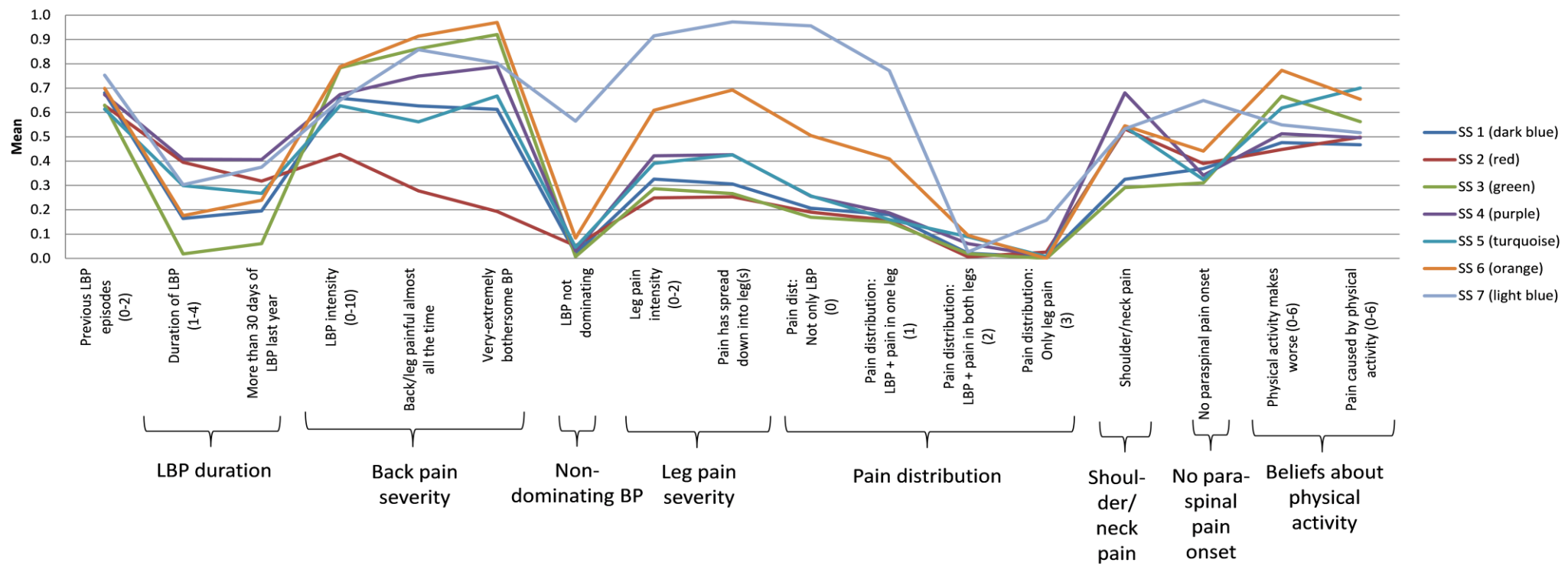

d

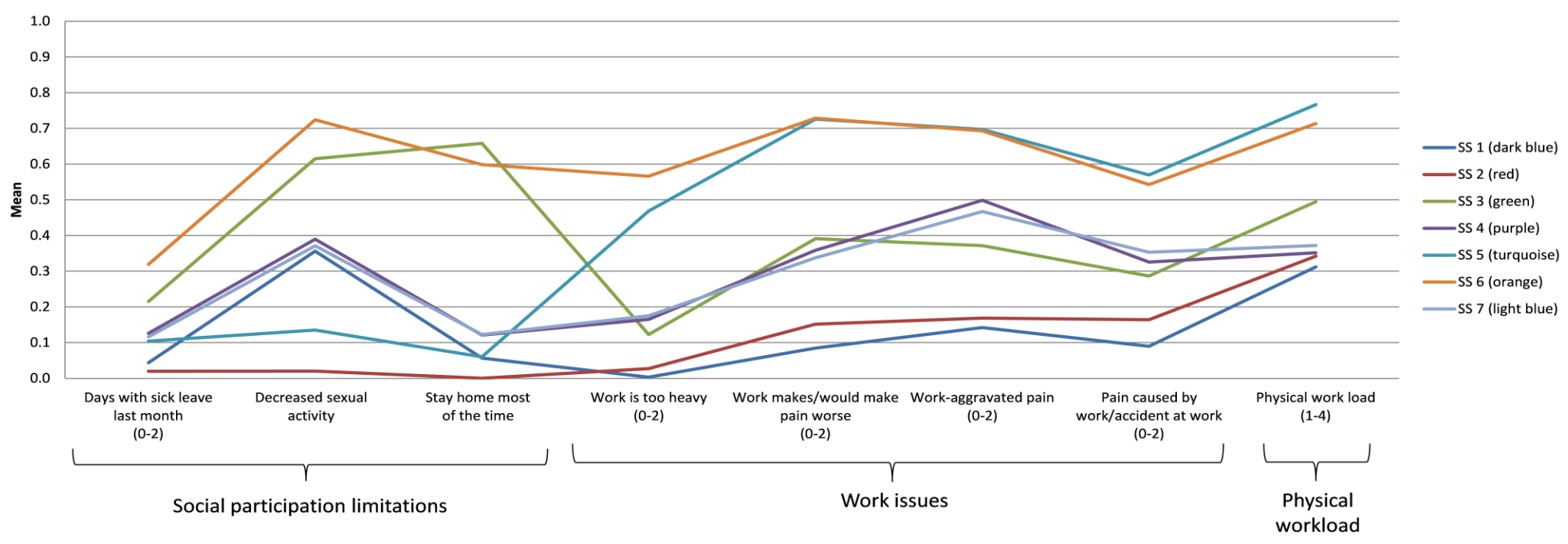

e

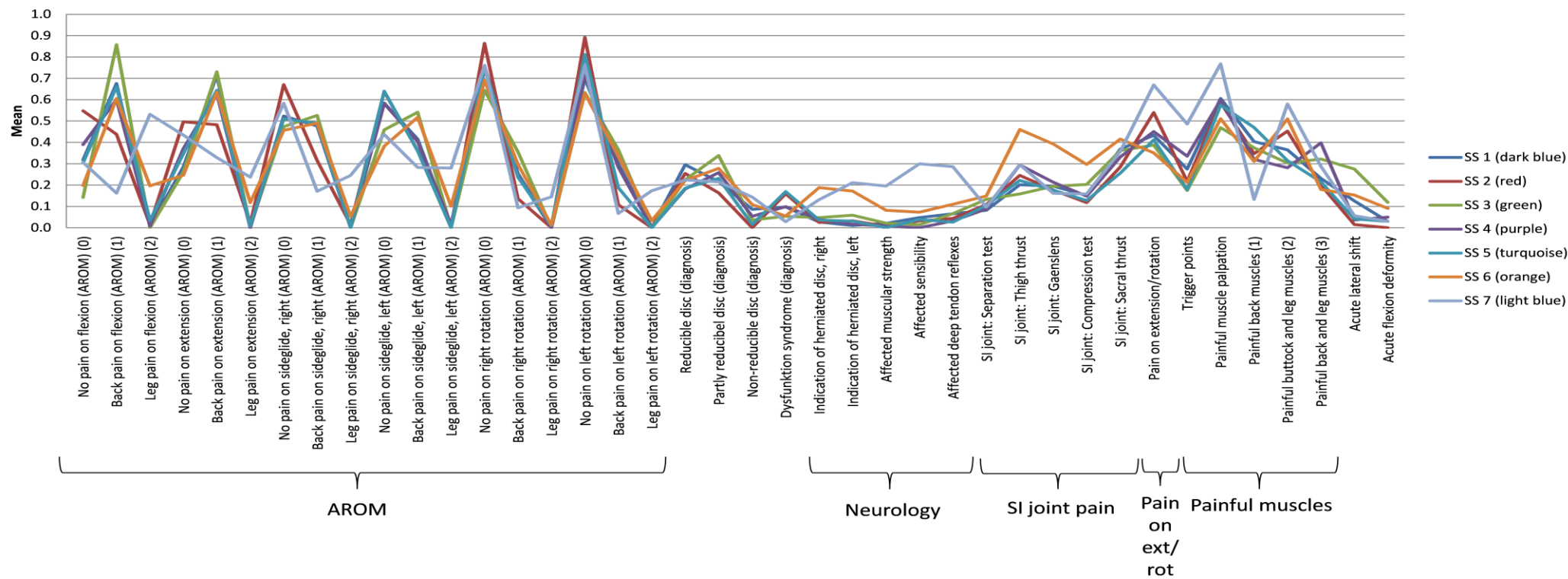

f

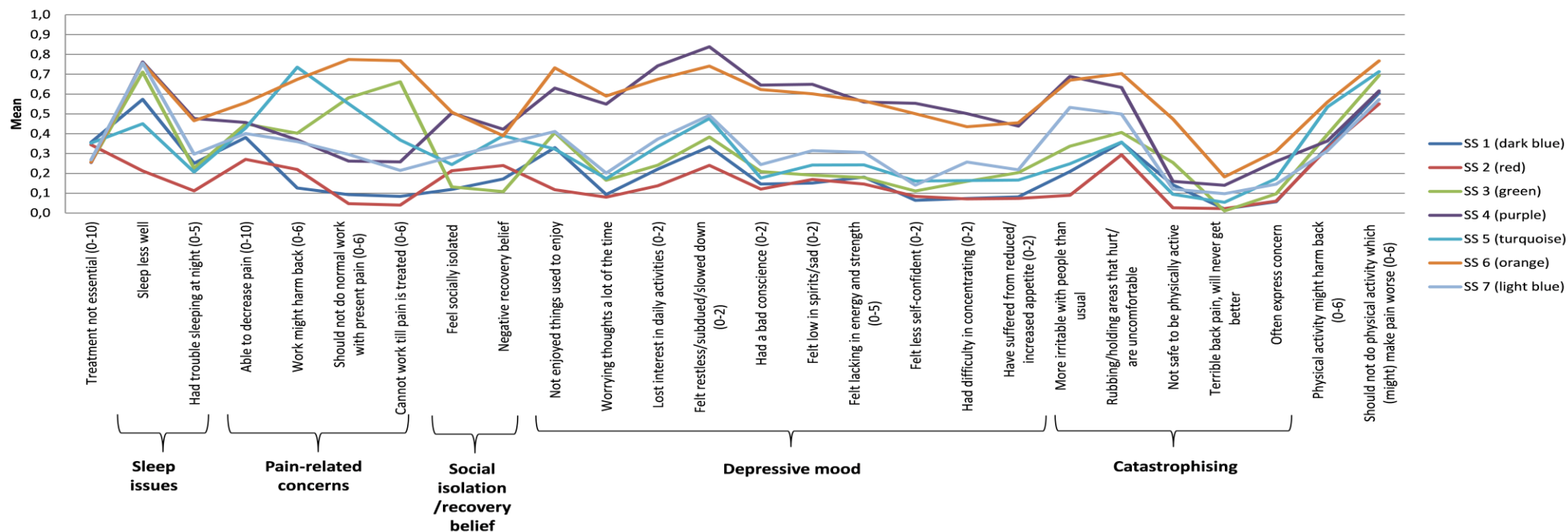

Supplement: Additional file 2: — Single-stage patient subgroups based on variables from the activity domain (a), the contextual factors domain (b), the pain domain (c), the participation domain (d), the physical impairment domain (e), and the psychology domain. (PDF 1.50 mb) [file 12891_2017_1411_MOESM2_ESM.pdf]

a

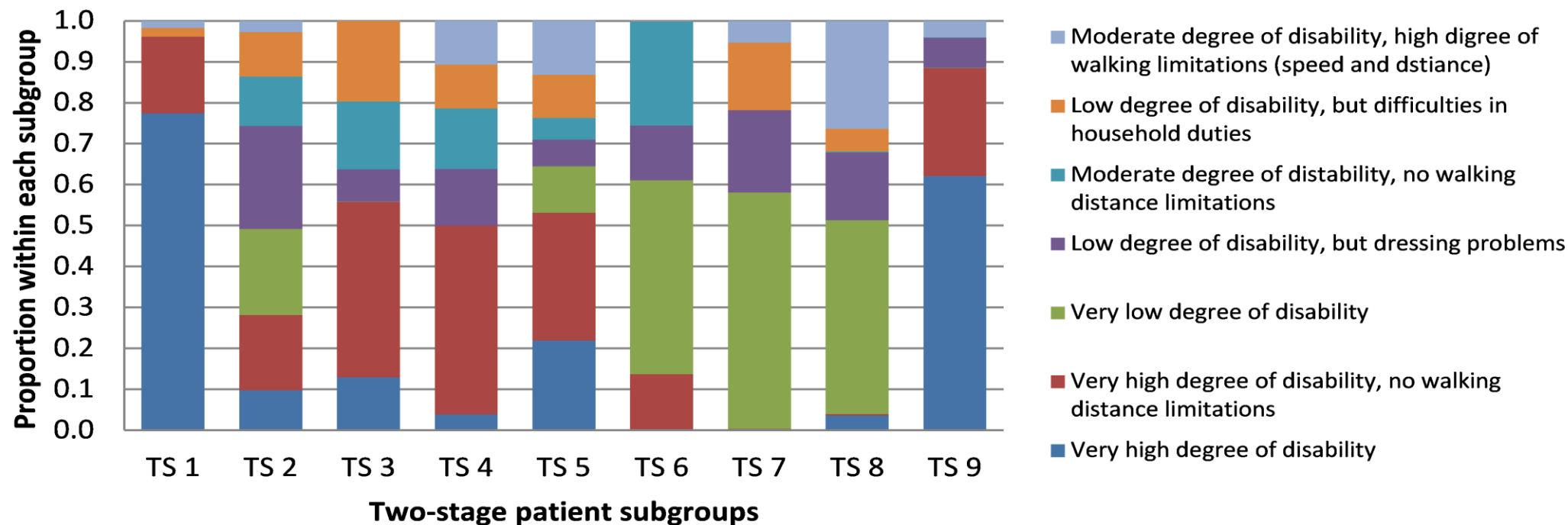

b

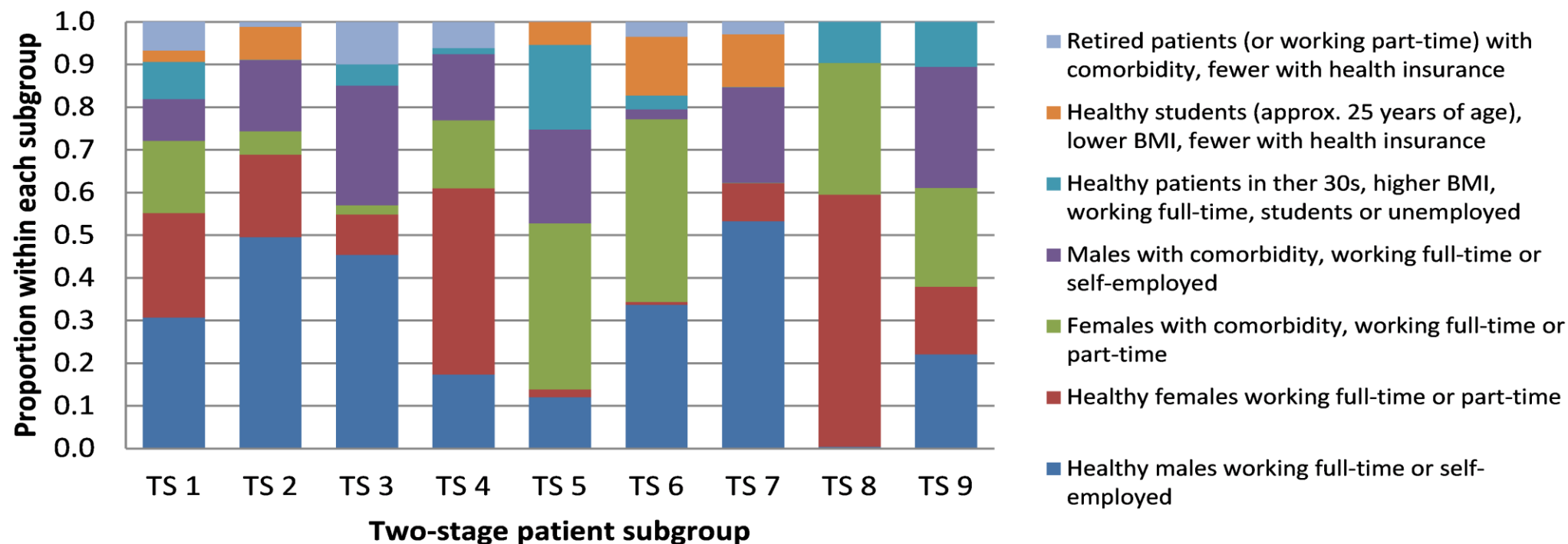

c

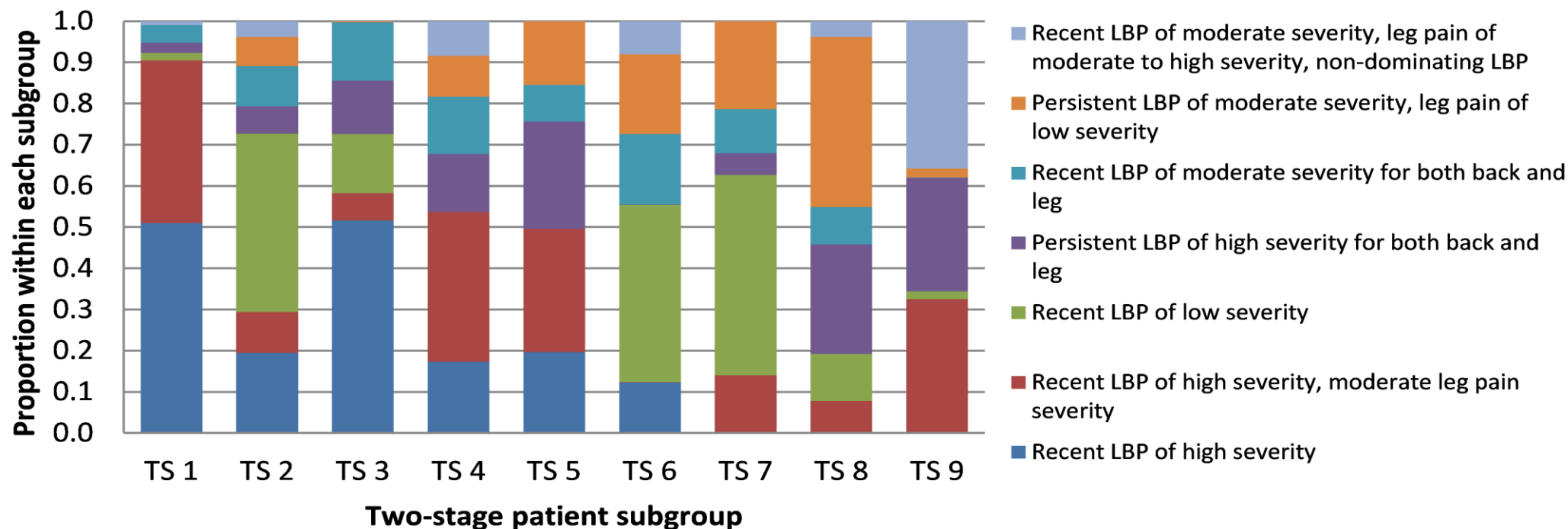

d

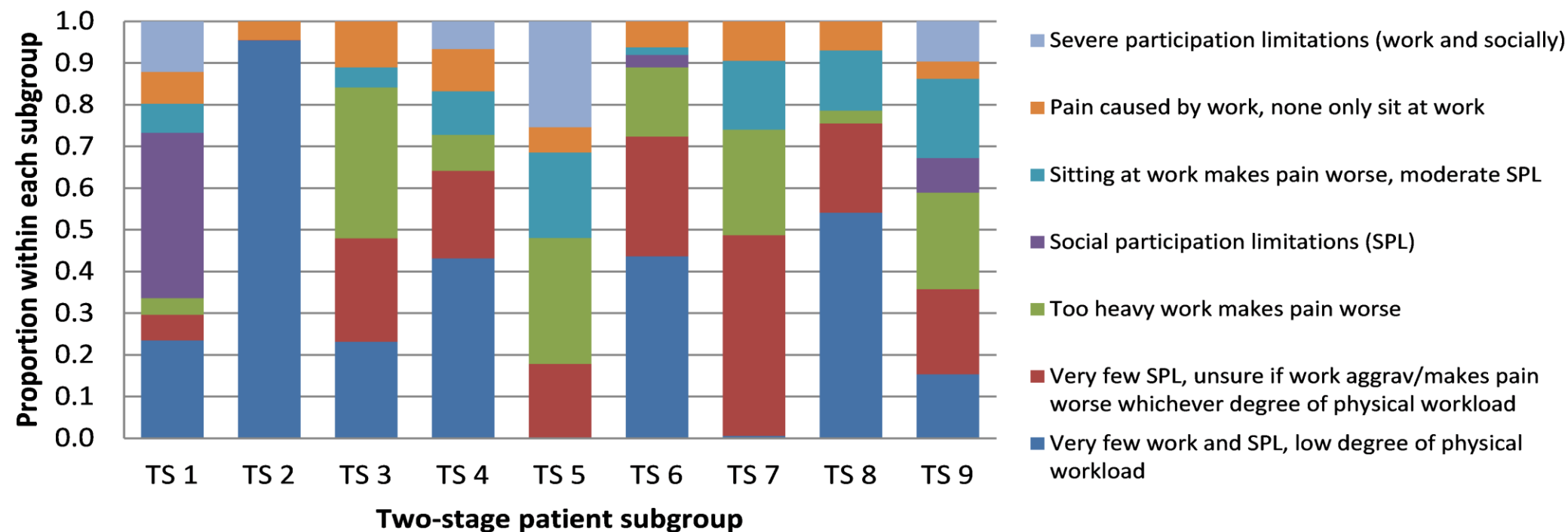

e

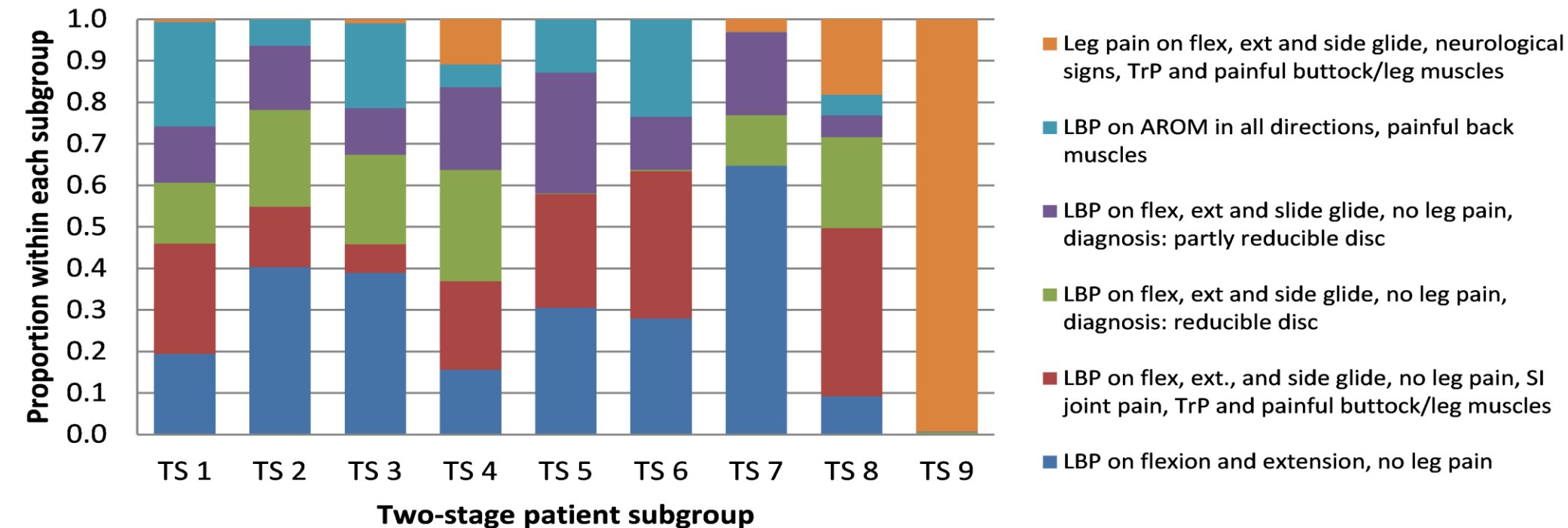

f

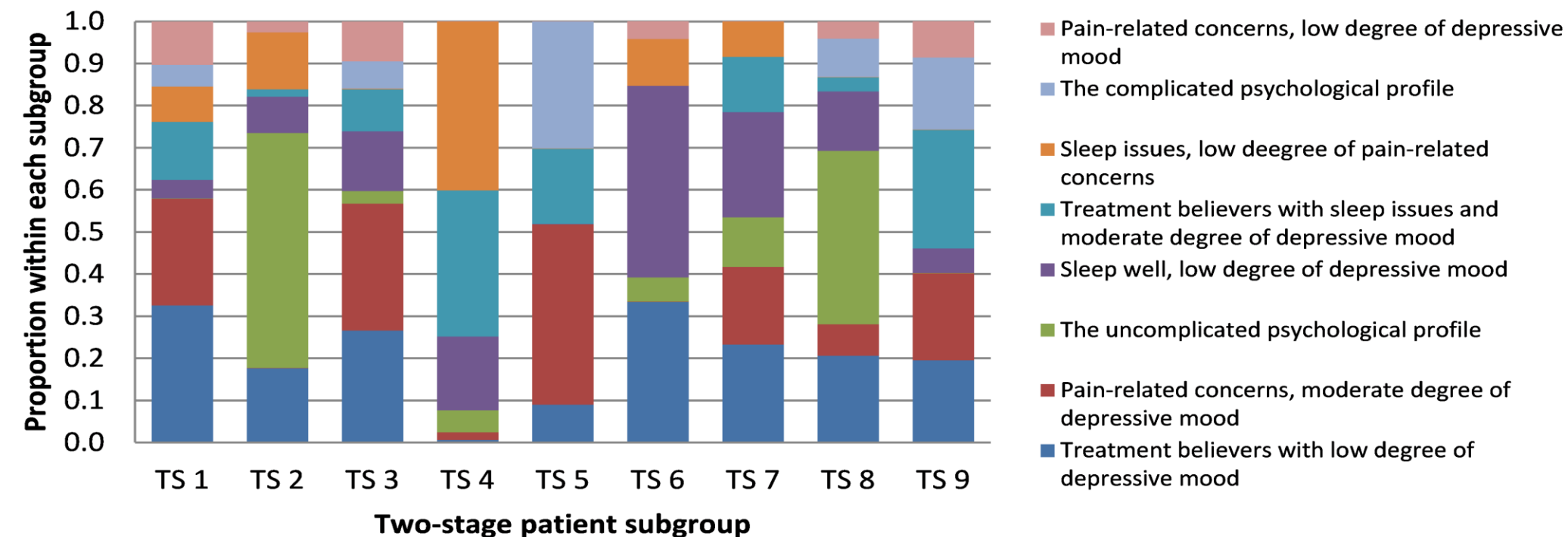

Supplement: Additional file 4: — Stacked bar chart for each two-stage patient subgroup based on the conditional probabilities of each domain-specific patient category (the identified latent variables from the first stage Latent Class Analysis) from the activity domain (a), the contextual factors domain (b), the pain domain (c), the participation domain (d), the physical impairment domain (e), and the psychology domain, respectively. (PDF 0.98 mb) [file 12891_2017_1411_MOESM4_ESM.pdf]
