# Supplementary material for: Identifying subgroups of patients using latent class analysis: should we use a single-stage or a two-stage approach? A methodological study using a cohort of patients with low back pain
Source: BMC Musculoskelet Disord. 2017 Feb 1;18:57. doi: 10.1186/s12891-017-1411-x (PMC5286735; doi:10.1186/s12891-017-1411-x)
Supplement: Additional file 3: — Descriptive presentation and profile plots of the preferred model solution for the second stage of the two-stage approach and for each of the domain-specific patient categorisations (result of the first stage of the two-stage LCA). Presentation of the standardised wording used in the descriptive process and tables for each of the preferred LCA models with descriptive presentation of each subgroup/category and associated profile plot. (PDF 10.4 mb) [file 12891_2017_1411_MOESM3_ESM.pdf]

Additional file 3

This document contains the descriptive presentation and profile plots of preferred model solutions for the two-stage LCA approach.
The patient subgroups (from the second stage) are presented first and subsequently the previously identified domain-specific patient categorisations (from the first stage) for each health domain.

Details of the used domain-specific patient categorisations (activity, pain, participation and psychology) have been published previously: Nielsen AM et al; *Using existing questionnaires in Latent Class Analysis: should we use summary scores or single items as input? A methodological study using a cohort of patients with low back pain*. Clin Epidemiol. 2016:73-89

Explanatory legend
We described each variable by using:

- standardised descriptive labels for quintiles (see Table 1)
- proportions (%) for nominal or ordinal variables with less than four categories
- interquartile ranges (IQR) for the remaining ordinal items and continuous items

Subgroup/category size and description of variables are based on the patients assigned to the subgroup/category for which their posterior probability was the largest

Table 1. Standardised wording used to report the quintiles within the estimates and subgroup profile plots

Range, %	0-19	20-39	40-59	60-79	80-100
'Very few to most'	Very few	Few	Half	Many	Most
'Low – high'	Very low	Low	Moderate	High	Very high
'All or none'	0=none				100=only, all

Table 2. Descriptive presentation of the nine two-stage (TS) patient subgroups based on categorical latent variables derived from the first stage of the two-stage LCA

Variable \ Patient subgroup		TS 1	TS 2	TS 3	TS 4	TS 5	TS 6	TS 7	TS 8	TS 9	Scale
Activity	Probabilities above 0.30	Act1=0.83	None	Act2=0.50	Act2=0.48	Act2=0.32	Act3=0.43	Act3=0.57	Act3=0.53	Act1=0.61	Multistate nominal<br>Act. 1- Act. 7
	Description	Very high degree of disability	Uncharacteristic profile	Very high degree of disability, no walking distance limitations<br><br>Overall: difficulties in household duties…	Very high degree of disability, no walking distance limitations	Very high degree of disability, no walking distance limitations<br><br>Overall: close to uncharacteristic	Very low degree of disability<br><br>Overall: no walking distance limitations	Very low degree of disability<br><br>Overall: more with dressing problems…	Very low degree of disability<br><br>Overall: Some with walking limitations	Very high degree of disability	
Contextual factors	Probabilities above 0.30	Context1=0.31	Context1=0.50	Context1=0.46<br>Context4=0.30	Context2=0.47	Context3=0.45	Context1=0.32<br>Context3=0.46	Context1=0.54	Context2=0.62	None	Multistate nominal<br>Context 1- Context 7
	Description	Healthy males working full-time or self-employed	Healthy males working full-time or self-employed	Healthy males OR males with comorbidity working full-time or self-employed	Healthy females working full-time or part-time	Females with comorbidity working full-time or part-time	Healthy males working full-time or self-employed OR females with comorbidity working full-time or part-time	Healthy males working full-time or self-employed	Healthy females working full-time or part-time	Uncharacteristic profile<br><br>Overall: more with comorbidity, less young students	
Pain	Probabilities above 0.30	Pain1=0.52<br>Pain2=0.39	Pain3=0.45	Pain1=0.56	Pain2=0.41	Pain2=0.30	Pain3=0.42	Pain3=0.50	Pain6=0.47	Pain2=0.33<br>Pain7=0.35	Multistate nominal<br>Pain 1- Pain 7
	Description	Recent LBP, high degree of LBP severity (some with leg pain, 'pain2')	Recent LBP with low degree of back pain severity	Recent LBP with high degree of LBP severity	Recent LBP with high degree of LBP severity and moderate degree of leg pain severity	Recent LBP with high degree of LBP severity and moderate degree of leg pain severity<br><br>*If taking the other categories into account:*<br>Back and leg pain of moderate-high severity, leg pain is not dominating	Recent LBP with low degree of back pain severity	Recent LBP with low degree of back pain severity	Persistent LBP of moderate severity, leg pain of low severity	Recent LBP of moderate-high severity for both back and leg pain<br><br>More<br>• with no dominating LBP	
Participation	Probabilities above 0.30	Part4=0.40	Part1=0.97	Part3=0.38	Part1=0.43	Part3=0.31	Part1=0.48	Part2=0.50	Part1=0.56	None	Multistate nominal<br>Part. 1- Part. 7
	Description	Social participation limitations	Very few work and SPL, low degree of physical workload	Too heavy work makes pain worse	Very few work and SPL, low degree of physical workload	Too heavy work makes pain worse<br><br>Overall: work-issues	Very few work and SPL, low degree of physical workload	Very few SPL, unsure if work aggrav/makes pain worse whichever degree of physical workload	Very few work and SPL, low degree of physical workload	Uncharacteristic profile<br><br>Overall: less with social participation	

										limitations/more with work participation limitations	
**Physical impairment**	Probabilities above 0.30	None	Phyimp1=0.43	Phyimp1=0.42	None	Phyimp2=0.30<br>Phyimp4=0.30	Phyimp2=0.39	Phyimp1=0.64	Phyimp2=0.42	Phyimp6=1.00	**Multistate nominal**<br>Phy.imp. 1-Phy.imp 6
	Description	Back pain, no leg pain	Mainly LBP on flexion and extension, no leg pain<br><br>More with the diagnosis: 'reducible disc'	Mainly LBP on flexion and extension, no leg pain<br><br>More with LBP on all directions and painful back muscles	Uncharacteristic profile	SI joint pain/TrP/painful buttock/leg muscles OR diagnosis: Partly reducible disc<br>No leg pain	SI joint pain, TrP and painful buttock/leg muscles, no leg pain	Mainly LBP on flexion and extension, no leg pain	SI joint pain, TrP and painful buttock/leg muscles	Leg pain on flex, ext and side glide. Neurological signs, TrP and painful buttok/leg muscles	
**Psychology**	Probabilities above 0.30	Psych1=0.33	Psych3=0.59	Psych2=0.32	Psych5=0.35<br>Psych6=0.44	Psych2=0.42<br>Psych7=0.34	Psych1=0.33<br>Psych4=0.49	None	Psych3=0.47	None	**Multistate nominal**<br>Psych 1-Psych8
	Description	Treatment believers with low degree of depressive mood<br><br>More<br>• believe treatment is essential<br>• sleep less well<br>• low degree of depressive mood	Uncomplicated psychological profile<br><br>Less<br>• sleep less well<br>• has pain-related concerns<br>• less feel socially isolated with depressive mood	Pain-related concerns, moderate degree of depressive mood<br><br>More<br>• sleep less well<br>• has pain-related concerns<br>• feel socially isolated<br>• with moderate degree of depressive mood	Sleep issues, low degree of pain-related concerns (low-moderate degree of depressive mood)<br><br>More<br>• with sleep issues<br><br>Less<br>• with pain-related concerns	Complicated psychological profile<br><br>More<br>• sleep less well<br>• with pain-related (work) concerns<br>• feel socially isolated<br>• moderate-high degree of depressive mood	Sleep well with low degree of depressive mood<br><br>More<br>• with low degree of depressive mood<br><br>Less<br>• sleep less well (psych4)	Not complicated psychological profile OR catastrophising	Uncomplicated psychological profile<br><br>Less<br>• sleep less well<br>• has pain-related concerns<br>• less feel socially isolated with depressive mood	Uncharacteristic psychological profile<br><br>More<br>• sleep less well	
**Labelling**		**Activity and social participation limitations, severe LBP**	**Copers with intermittent and mild LBP**	**Work-related severe LBP**	**Sleep issues, activity limitations, severe LBP**	**LBP with several consequences**	**Sleeps well and SI joint pain**	**Mild LBP with few consequences**	**Persistent LBP with SI joint pain**	**Nerve root involvement**	
**Subgroup size, N**		219 (24%)	161 (17%)	127 (14%)	113 (12%)	71 (8%)	69 (7%)	74 (8%)	45 (5%)	49 (5%)	
**Posterior probability, median**		0.98<br>(IQR=0.82-1.00)	0.92<br>(IQR=0.79-0.98)	0.83<br>(IQR=0.63-0.95)	0.87<br>(IQR=0.74-0.94)	0.87<br>(IQR=0.70-0.98)	0.89<br>(IQR=0.68-0.98)	0.85<br>(IQR=0.69-0.98)	0.94<br>(IQR=0.87-0.98)	0.98<br>(IQR=0.91-1.00)	
**Posterior probability less than 0.70, N**		32<br>(15%)	26<br>(16%)	41<br>(32%)	24<br>(21%)	17<br>(24%)	18<br>(26%)	19<br>(26%)	4<br>(9%)	3<br>(6%)	

TS = two-stage patient subgroup
Psych = psychology domain-specific categorisation
LBP = low back pain
Act = activity domain-specific categorisation
Part = participation domain-specific categorisation
SPL = social participation limitations
Phy.imp. = physical impairment domain-specific categorisation
SI = sacroiliac
TrP = trigger points
Context = contextual factors domain-specific categorisation

Figure 1. Profile plots of three model solutions for patient subgroups resulting from the second stage of the two-stage LCA

Description of the domain-specific patient categorisations (6 variables)

Psych_1: Treatment believers with low degree of depressive mood
Psych_2: Pain-related concerns, moderate degree of depressive mood
Psych_3: Uncomplicated psychological profile
Psych_4: Sleep well, low degree of depressive mood
Psych_5: Treatment believers with sleep issues and moderate degree of depressive mood
Psych_6: Sleep issues, low degree of pain-related concerns
Psych_7: The complicated psychological profile
Psych_8: Pain-related concerns, low degree of depressive mood
Pain_1: Recent LBP with high degree of back pain severity
Pain_2: Recent LBP with high degree of back pain severity and moderate degree of leg pain severity
Pain_3: Recent LBP with low degree of back pain severity
Pain_4: Persistent LBP, high degree of back and leg pain severity
Pain_5: Recent LBP with moderate degree of back pain severity, moderate degree of leg pain severity
Pain_6: Persistent LBP, moderate degree of back pain severity and low degree of leg pain severity
Pain_7: Recent LBP, moderate degree of LBP severity, moderate-high degree of LP severity, ND-LBP
Act_1: Very high degree of disability (transfer/climbing/bending/turning/walking/dressing/household/work)
Act_2: Very high degree of disability, but no walking distance limitation
Act_3: Very low degree of disability
Act_4: Low degree of disability, but dressing problems
Act_5: Moderate degree of disability, no walking distance limitations
Act_6: Low degree of disability, but difficulties in household duties
Act_7: Moderate degree of disability, high degree of walking limitations (speed and distance)
Part_1: Very few work and SPL, low degree of physical workload
Part_2: Very few SPL, unsure if work aggrav./makes worse, whichever degree of physical workload
Part_3: Very few SPL, work is too heavy, aggrav./makes worse, pain caused by or at work, high degree of PW
Part_4: High degree of SPL, but low degree of work limitations whichever degree of physical workload
Part_5: Moderate degree of SPL, work aggravated/makes pain worse, very low degree of physical workload
Part_6: Low degree of SPL, pain caused by work, none with very low degree of physical workload
Part_7: High degree of SPL, work is too heavy, aggrav./makes worse, pain caused by work, high degree of PW
Phy.imp_1: LBP on flexion and extension, no LP
Phy.imp_2: LBP on flex, ext, and side glide, no LP, *SI joint pain*, TP and painful buttock/leg muscles
Phy.imp_3: LBP on flex, ext and side glide, no LP, *diagnosis: reducible disc*
Phy.imp_4: LBP on flex, ext and side glide, no LP, *diagnosis: partly reducible disc*
Phy.imp_5: LBP on AROM in all directions, painful back muscles
Phy.imp_6: LP on flex, ext and side glide, neurological signs, TP and painful buttock/leg muscles
Context 1: Healthy males working full-time or self-employed
Context 2: Healthy females working full-time or part-time
Context 3: Females with comorbidity, working full-time or part-time
Context 4: Males with comorbidity, working full-time or self-employed
Context 5: Healthy patients in their 30s, higher BMI, working full-time, students or unemployed
Context 6: Healthy students (approx. 25 years of age), lower BMI, fewer with health insurance
Context 7: Retired patients (or working part-time) with comorbidity, fewer with health insurance

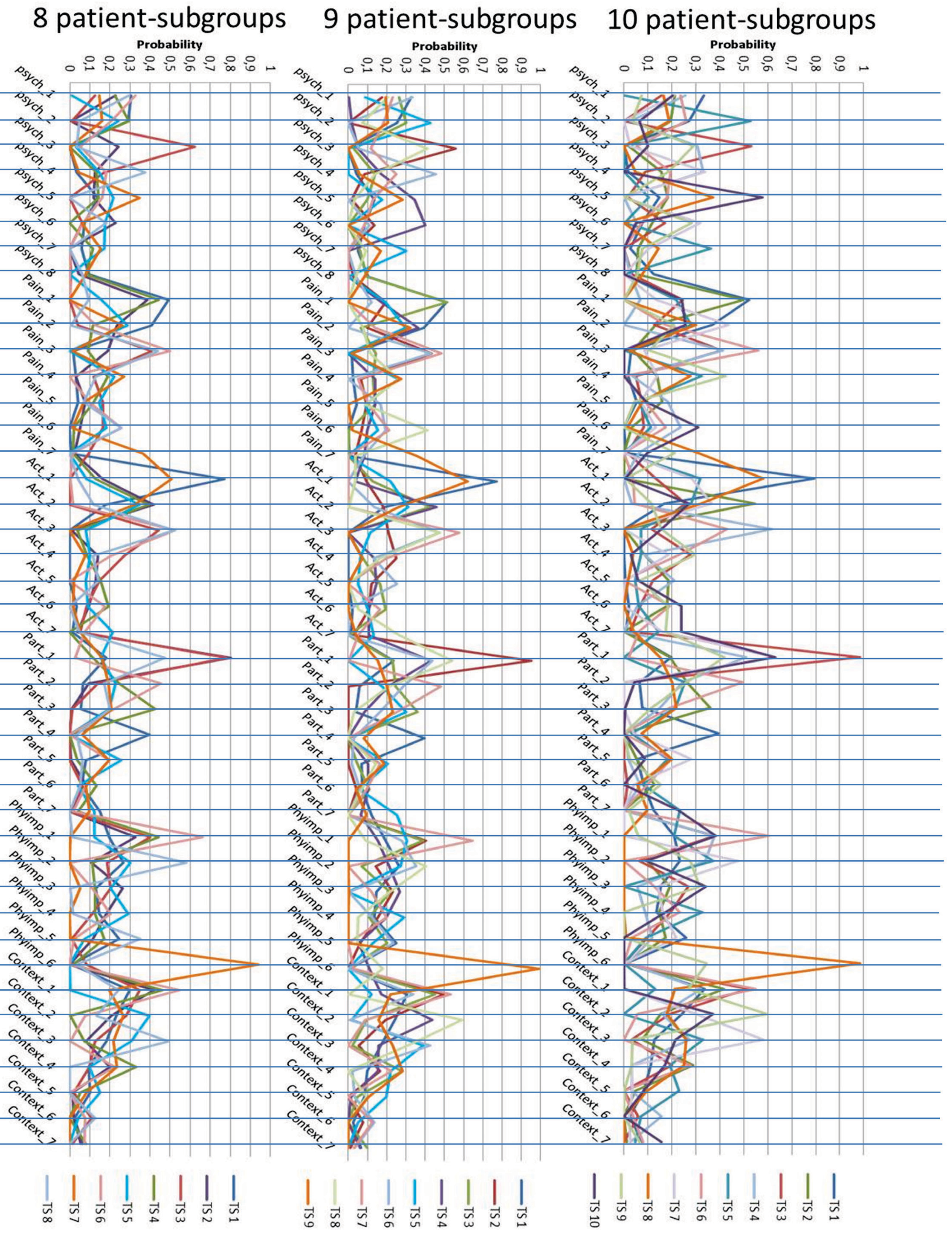

NOTE: Aggrav. = aggravated; AROM = active range of motion; BMI = body mass index; Ext = extension, Flex = flexion, LBP = low back pain; LP = leg pain; ND-LBP = non-dominating LBP; PW = physical workload; SI joint = sacroiliac joint, SPL = social participation limitations; TP = trigger points

Table 3. Descriptive presentation of the domain-specific patient categories identified in the activity domain by the first stage of the two-stage LCA

Variable \ Category	Act 1	Act 2	Act 3	Act 4	Act 5	Act 6	Act 7	Scale
**Best activity is *not* to walk***	**Half do not enjoy walking (47%)**	Few do not enjoy walking (25%)	Few do not enjoy walking (29%)	Few do not enjoy walking (33%)	Few do not enjoy walking (27%)	Few do not enjoy walking (26%)	**Most do not enjoy walking (83%)**	**Dichotomous** 0=no 1=yes
**Only stand for short periods of time (RMDQ-8)***	**Half only stand for short periods (59%)**	Very few only stand for short periods (18%)	**Very few only stand for short periods (7%)**	**Very few only stand for short periods (3%)**	**Very few only stand for short periods (5%)**	Very few only stand for short periods (16%)	**Many only stand for short periods (61%)**	**Dichotomous** 0=no 1=yes
**Only walk short distances (RMDQ-14)***	**Most only walk short distances (98%)**	Few only walk short distances (21%)	**Very few only walk short distances (1%)**	**Very few only walk short distances (1%)**	**Very few only walk short distances (1%)**	Most only walk short distances (16%)	**All only walk short distances (100%)**	**Dichotomous** 0=no 1=yes
**Only walked short distances (SBT-3)***	**Many have only walked short distances (69%)**	Few have only walked short distances (27%)	**Very few have only walked short distances (2%)**	**Very few have only walked short distances (7%)**	**Very few have only walked short distances (3%)**	Very few have only walked short distances (19%)	**Most have only walked short distances (86%)**	**Dichotomous** 0=disagree 1=agree
**Use handrail to get upstairs (RMDQ-5)**	**Most use handrail to get upstairs (88%)**	**Half use handrail to get upstairs (51%)**	**Very few use handrail to get upstairs (8%)**	Very few use handrail to get upstairs (11%)	Few use handrail to get upstairs (35%)	Few use handrail to get upstairs (33%)	**Half use handrail to get upstairs (47%)**	**Dichotomous** 0=no 1=yes
**Hold on to something to get out of an easy chair (RMDQ-6)****	**Most hold on to something to get out of an easy chair (94%)**	**Many hold on to something to get out of an easy chair (79%)**	**None hold on to something to get out of an easy chair (0%)**	Few hold on to something to get out of an easy chair (20%)	**Many hold on to something to get out of an easy chair (68%)**	Few hold on to something to get out of an easy chair (33%)	Few hold on to something to get out of an easy chair (31%)	**Dichotomous** 0=no 1=yes
**Difficult to get out of a chair (RMDQ-10)****	**Most with difficulties getting out of a chair (96%)**	**Most with difficulties getting out of a chair (95%)**	**Very few with difficulties getting out of a chair (1%)**	Few with difficulties getting out of a chair (25%)	**Many with difficulties getting out of a chair (72%)**	Few with difficulties getting out of a chair (33%)	Few with difficulties getting out of a chair (22%)	**Dichotomous** 0=no 1=yes
**Difficult to turn over in bed (RMDQ-12)*****	**Most with difficulties turning in bed (90%)**	**Most with difficulties turning in bed (84%)**	**Few with difficulties turning in bed (21%)**	Half with difficulties turning in bed (40%)	Half with difficulties turning in bed (53%)	Half with difficulties turning in bed (40%)	Half with difficulties turning in bed (53%)	**Dichotomous** 0=no 1=yes
**Try not to bend or kneel down (RMDQ-9)*****	**Most try not to bend or kneel down (92%)**	**Most try not to bend or kneel down (80%)**	**Few try not to bend or kneel down (19%)**	Half try not to bend or kneel down (55%)	Half try not to bend or kneel down (58%)	Half try not to bend or kneel down (59%)	Half try not to bend or kneel down (51%)	**Dichotomous** 0=no 1=yes
**Dressed more slowly last two weeks (SBT-4)[¤]**	**Most have dressed more slowly (81%)**	**Most have dressed more slowly (96%)**	**Very few have dressed more slowly (10%)**	**All have dressed more slowly (100%)**	Very few have dressed more slowly (19%)	Few have dressed more slowly (25%)	Half have dressed more slowly (43%)	**Dichotomous** 0=disagree 1=agree
**Get dressed more slowly (RMDQ-7) [¤]**	**Most get dressed more slowly (98%)**	**Most get dressed more slowly (99%)**	**Very few get dressed more slowly (1%)**	**Most get dressed more slowly (98%)**	Few get dressed more slowly (34%)	Very few get dressed more slowly (16%)	Few get dressed more slowly (33%)	**Dichotomous** 0=no 1=yes
**Trouble putting on socks (RMDQ-13) [¤]**	**Most have trouble putting on socks (95%)**	**Most have trouble putting on socks (96%)**	**Very few have trouble putting on socks (3%)**	**Most have trouble putting on socks (85%)**	Half have trouble putting on socks (50%)	Few have trouble putting on socks (27%)	Few have trouble putting on socks (35%)	**Dichotomous** 0=no 1=yes
**Walk more slowly (RMDQ-3)[¤¤]**	**All walk more slowly (100%)**	**Most walk more slowly (82%)**	**Very few walk more slowly (16%)**	Few walk more slowly (33%)	Half walk more slowly (44%)	Half walk more slowly (49%)	**Most walk more slowly (94%)**	**Dichotomous** 0=no 1=yes
**Go upstairs more slowly (RMDQ-18) [¤¤]**	**All go upstairs more slowly (100%)**	**Many go upstairs more slowly (78%)**	**Very few go upstairs more slowly (14%)**	Few go upstairs more slowly (26%)	Half go upstairs more slowly (44%)	Half go upstairs more slowly (48%)	**Many go upstairs more slowly (73%)**	**Dichotomous** 0=no 1=yes
**Avoid heavy jobs around the house (RMDQ-16)[¤¤¤]**	**Most avoid heavy jobs (99%)**	**Most avoid heavy jobs (86%)**	**Few avoid heavy jobs (27%)**	Half avoid heavy jobs (64%)	Half avoid heavy jobs (47%)	**Most avoid heavy jobs (98%)**	**Most avoid heavy jobs (92%)**	**Dichotomous** 0=no 1=yes
**Do less daily work around the house (RMDQ-22) [¤¤¤]**	**Most do less of the daily work (96%)**	**Most do less of the daily work (80%)**	**Very few do less of the daily work (10%)**	Few do less of the daily work (37%)	**Very few do less of the daily work (9%)**	**Most do less of the daily work (99%)**	Many do less of the daily work (65%)	**Dichotomous** 0=no 1=yes
**Not doing usual jobs around the house**	**Most are not doing any usual jobs**	**Half are not doing any usual jobs**	**Very few are not doing any usual jobs**	Few are not doing any usual jobs	**Very few are not doing any usual jobs**	**Many are not doing any usual jobs**	Half are not doing any usual jobs	**Dichotomous** 0=no

**(RMDQ-4)** [¤¤¤]	**(85%)**	**(60%)**	**(9%)**	(31%)	**(17%)**	**(73%)**	(45%)	1=yes
**Cannot do physical activities which (might) make worse (FABQ-5)** [ǂ]	Many cannot do physical activities which (might) make my pain worse<br><br>Disagree=18%<br>Unsure=22%<br>**Agree=60%**	Half cannot do physical activities which (might) make my pain worse<br><br>Disagree=33%<br>Unsure=21%<br>Agree=46%	Very few cannot do physical activities which (might) make my pain worse<br><br>**Disagree=58%**<br>Unsure=28%<br>Agree=14%	Very few cannot do physical activities which (might) make my pain worse<br><br>**Disagree=63%**<br>Unsure=22%<br>Agree=15%	Few cannot do physical activities which (might) make my pain worse<br><br>**Disagree=57%**<br>Unsure=18%<br>Agree=25%	Half cannot do physical activities which (might) make my pain worse<br><br>Disagree=31%<br>Unsure=28%<br>Agree=41%	Few cannot do physical activities which (might) make my pain worse<br><br>Disagree=42%<br>Unsure=21%<br>Agree=38%	**Ordinal**<br>0=completely disagree<br>3=unsure<br>6=completely agree<br>*For description analysis, trichotomised:*<br>*0=disagree (0-2)*<br>*1=unsure (3)*<br>*2=agree (4-6)*
**Cannot work with present pain (FABQ-13)** [ǂ]	**Half cannot work with present pain**<br><br>**Disagree=33%**<br>**Unsure=9%**<br>**Agree=58%**	Few cannot work with present pain<br><br>Disagree=68%<br>Unsure=7%<br>Agree=25%	Very few cannot work with present pain<br><br>**Disagree=91%**<br>Unsure=5%<br>Agree=4%	Very few cannot work with present pain<br><br>**Disagree=91%**<br>Unsure=4%<br>Agree=5%	Very few cannot work with present pain<br><br>**Disagree=81%**<br>Unsure=7%<br>Agree=12%	Few cannot work with present pain<br><br>Disagree=63%<br>Unsure=9%<br>Agree=28%	Few cannot work with present pain<br><br>Disagree=70%<br>Unsure=5%<br>Agree=24%	**Ordinal**<br>0=disagree<br>1=unsure<br>2=agree
**Best posture to sit**	Few enjoy sitting<br>(24%)	Few enjoy sitting<br>(21%)	Few enjoy sitting<br>(23%)	**Very few enjoy sitting**<br>**(12%)**	Very few enjoy sitting<br>(18%)	Few enjoy sitting<br>(33%)	Few enjoy sitting<br>(29%)	**Dichotomous**<br>0=no<br>1=yes
**Stay in bed most of the time (RMDQ-19)**	**Very few stay in bed**<br>**(19%)**	Very few stay in bed<br>(2%)	Very few stay in bed<br>(0%)	Very few stay in bed<br>(0%)	Very few stay in bed<br>(1%)	Very few stay in bed<br>(1%)	Very few stay in bed<br>(4%)	**Dichotomous**<br>0=no<br>1=yes
**Change position frequently (RMDQ-2)**	Most change position frequently<br>(92%)	Most change position frequently<br>(92%)	**Many change position frequently**<br>**(71%)**	Most change position frequently<br>(83%)	Most change position frequently<br>(90%)	Most change position frequently<br>(84%)	Most change position frequently<br>(92%)	**Dichotomous**<br>0=no<br>1=yes
**Labelling and clinical description**	**Very high degree of disability (transfer/climbing, bending/turning, walking, dressing, household, work)**<br><br>**More**<br>• with walking limitations (*do not enjoy walking, only stand for a short period of time, have only walked and walks only short distances)*<br>• use handrail to get upstairs<br>• with difficulties getting out of a chair<br>• with difficulties turning over in bed<br>• try not to bend or kneel down<br>• with dressing problems<br>• walk slowly	**Very high degree of disability, but no walking distance limitation**<br><br>**More**<br>• use handrail to get upstairs<br>• with difficulties getting out of a chair<br>• with difficulties turning over in bed<br>• try not to bend or kneel down<br>• with dressing problems<br>• walk slowly<br>• with difficulties doing household duties	**Very low degree of disability**<br><br>**Less**<br>• with walking limitation<br>• Change position frequently<br>• use handrail to get upstairs<br>• with difficulties getting out of a chair<br>• with difficulties turning over in bed<br>• try not to bend or kneel down<br>• with dressing problems<br>• walk slowly<br>• with difficulties doing household duties<br>• cannot do physical activities which make pain worse<br>• cannot work with present pain	**Low degree of disability, but dressing problems**<br><br>**More**<br>• with dressing problems<br><br>**Less**<br>• with walking limitation<br>• cannot do physical activities which make pain worse<br>• cannot work with present pain<br>• enjoy sitting	**Moderate degree of disability, no walking distance limitations (transfer and climbing problems, no trouble with normal household duties)**<br><br>**More**<br>• with difficulties getting out of a chair<br><br>**Less**<br>• with walking limitation<br>• cannot do physical activities which make pain worse<br>• cannot work with present pain	**Low degree of disability, but difficulties in household duties**<br><br>**More**<br>• with difficulties doing household duties	**Moderate degree of disability, high degree of walking limitations (speed and distance)**<br><br>**More**<br>• with walking limitation *(do not enjoy walking, only stand for a short period of time, have only walked and walks only short distances)*<br>• use handrail to get upstairs<br>• walk slowly<br>• avoid heavy jobs	

	• with difficulties doing household duties<br>• cannot do physical activities which make pain worse<br>• cannot work with present pain<br>• stay in bed							
**Category size, N**	254 (27%)	223 (24%)	137 (15%)	105 (11%)	79 (9%)	81 (9%)	49 (5%)	
**Posterior probability, median**	0.98 (IQR=88-100)	0.95 (IQR=83-99)	0.99 (IQR=92-100)	0.95 (IQR=80-99)	0.96 (IQR=78-100)	0.93 (IQR=87-98)	0.96 (IQR=85-99)	
**Posterior probability less than 0.70, N**	27 (11%)	30 (13%)	13 (9%)	18 (17%)	15 (19%)	12 (15%)	5 (10%)	

Bold text indicates characteristic features which mainly identify the respective domain-specific patient categories
RMDQ = Roland-Morris Disability Questionnaire; FABQ = Fear-Avoidance Beliefs Questionnaire; SBT = STarT Back Tool; IQR = interquartile range
Symbols and light lines: Variables which have similar scoring pattern across domain-specific patient categories representing an overall feature:
*Walking distance limitations
**Transfer and climbing problems
***Bending/turning challenges
¤ Dressing problems
¤¤ Walking speed limitations
¤¤¤ Difficulties in household duties
ƚ Cannot work with present pain

Figure 2. Profile plot of the domain-specific patient categories identified in the activity domain by the first stage of the two-stage LCA

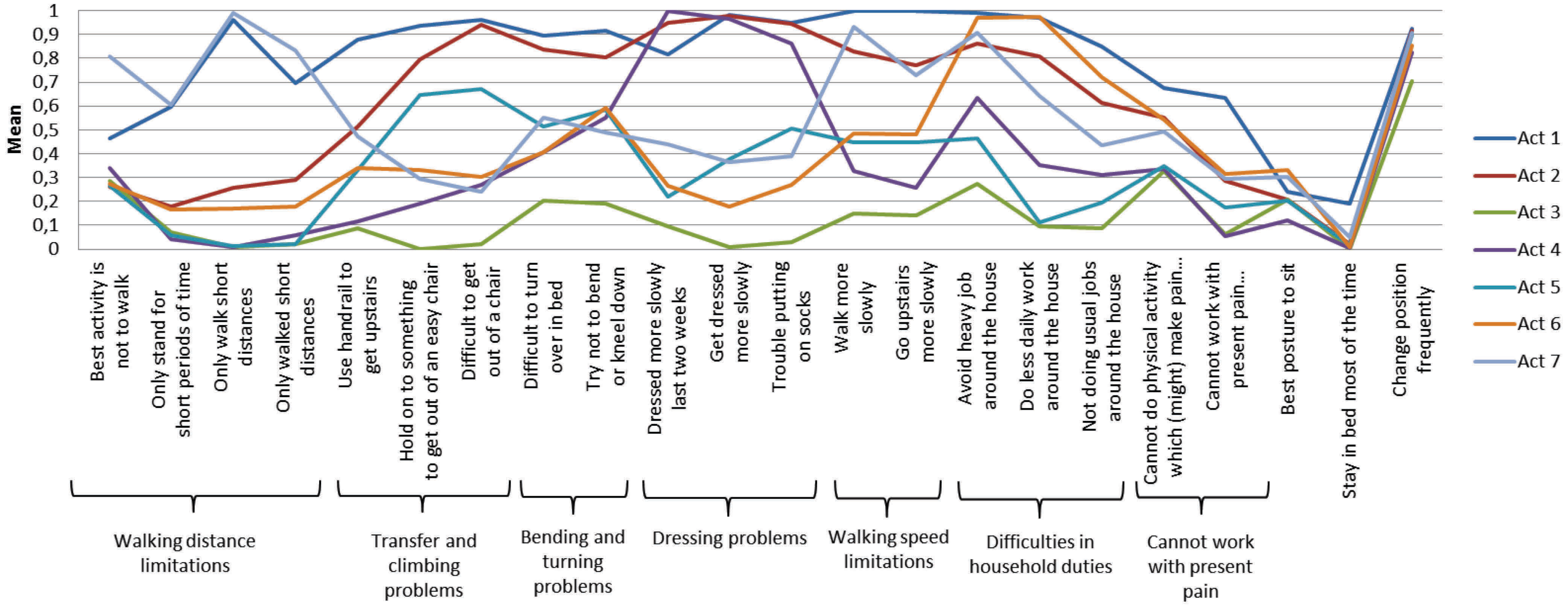

Brief conceptual description of domain-specific patient categories identified in the activity domain (first stage of two-stage LCA):

Act 1: Very high degree of disability (transfer/climbing, bending/turning, walking, dressing, household, work)
Act 2: Very high degree of disability, but no walking distance limitation
Act 3: Very low degree of disability
Act 4: Low degree of disability, but dressing problems
Act 5: Moderate degree of disability, no walking distance limitations
Act 6: Low degree of disability, but difficulties in household duties
Act 7: Moderate degree of disability, high degree of walking limitations (speed and distance)

Table 4. Descriptive presentation of the domain-specific patient categories identified in the contextual factors domain by the first stage of the two-stage LCA

Category / Feature	Context 1	Context 2	Context 3	Context 4	Context 5	Context 6	Context 7	Scale
**Age in years, median**	42<br>(IQR=35-50)	44<br>(IQR=36-53)	46<br>(IQR=37-54)	46<br>(IQR=37-55)	32<br>(IQR=29-37)	24<br>(IQR=22-26)	64<br>(IQR=63-65)	**Continuous**<br>18-66 years of age
**Sex**	Males<br>(100%)	Very few males<br>(1%)	No males<br>(0%)	Males<br>(100%)	Few males<br>(31%)	Half males<br>(50%)	Half males<br>(54%)	**Dichotomous**<br>0=female<br>1=male
**Height, median**	182 cm<br>(IQR=178-187)	169 cm (IQR=165-173)	168 cm (IQR=165-172)	181 cm (IQR=178-186)	174 cm (IQR=170-180)	179 cm (IQR=173-184)	170 cm (IQR=167-178)	**Continuous**<br>156-201 cm
**BMI, median**	26<br>(IQR=24-28)	24<br>(IQR=22-26)	25<br>(IQR=22-28)	27<br>(IQR=24-30)	32<br>(IQR=28-37)	23<br>(IQR=21-24)	28<br>(IQR=24-33)	**Continuous**<br>Range: 18-59
**Work situation**	Self-employed (15%)<br>Full-time (80%)	Self-employed (9%)<br>Full-time (59%)<br>Part-time (27%)	Full-time (47%)<br>Part-time (30%)<br>Unemployed (6%)	Self-employed (20%)<br>Full-time (67%)<br>Unemployed (8%)	Full-time (56%)<br>Part-time (11%)<br>Student (24%)<br>Unemployed (9%)	Full-time (5%)<br>Part-time (15%)<br>Student (78%)	Part-time (10%)<br>Early retirement/retired (69%)<br>Health-related retirement (21%)	**Multistate nominal**<br>1=self-employed<br>2=full-time<br>3=part-time<br>4=student<br>5=unemployed<br>6=early retirement/retired<br>7=health-related retirement<br>8=other
**Comorbidity**	Very **seldom** other chronic disease (0%)<br>*Other (1%, current/previous cancer)*	Very **seldom** other chronic disease (0%)<br>*Other (4%, 'current/previous cancer')*	**All** with other chronic disease (100%):<br>Heart (15%)<br>Asthma (20%)<br>Psychological (14%)<br>Musculoskeletal (34%)<br>Other (38%)	**All** with other chronic disease (100%):<br>Heart (26%)<br>Asthma (20%)<br>Psychological (5%)<br>Musculoskeletal (32%)<br>Other (28%)	Very **seldom** other chronic disease (4%)<br>Other (2%)	Very **seldom** other chronic disease (18%):<br>Asthma (10%)<br>Musculoskeletal (8%)<br>Other (3%)	Very **often** other chronic disease (85%):<br>Heart (44%)<br>Asthma (12%)<br>Psychological (10%)<br>Musculoskeletal (20%)<br>Other (32%)	**Dichotomous (6 items)**<br>0=no<br>1=yes<br>No other chronic disease<br>Heart disease<br>Asthma<br>Psychological disease<br>Musculoskeletal disease<br>Other chronic disease
**Health insurance (private or work related)**	Half have health insurance<br>(54%)	Half have health insurance<br>(42%)	Few have health insurance<br>(38%)	Half have health insurance<br>(54%)	Few have health insurance<br>(44%)	Very few have health insurance<br>(8%)	Very few have health insurance<br>(3%)	**Dichotomous**<br>0=no<br>1=yes<br>*(reversed in the profile plot)*
**Highest educational level**	1=6%<br>**2=36%**<br>3=14%<br>**4=26%**<br>5=19%	1=4%<br>2=13%<br>3=19%<br>**4=48%**<br>5=16%	1=7%<br>2=17%<br>**3=20%**<br>**4=46%**<br>5=10%	1=7%<br>**2=36%**<br>3=10%<br>**4=32%**<br>5=15%	**1=24%**<br>**2=24%**<br>**3=22%**<br>**4=22%**<br>5=7%	**1=43%**<br>2=5%<br>3=15%<br>4=18%<br>**5=20%**	1=16%<br>**2=34%**<br>3=11%<br>**4=32%**<br>5=8%	**Ordinal**<br>1=no qualification<br>2=vocational training<br>3=higher education <3 years<br>4=higher education 3-4 years<br>5=higher education >4 years<br>*(reversed in the profile plot)*
**Self-rated general health**	75<br>(IQR=60-85)	80<br>(IQR=60-86)	70<br>(IQR=50-80)	70<br>(IQR=53-80)	40<br>(IQR=29-60)	80<br>(IQR=70-85)	61<br>(IQR=50-79)	**Continuous**<br>Range: 0-100<br>0= lowest<br>100=highest<br>*(reversed in the profile plot)*
**Smoking status**	**Very few smokers**<br>Smoker (19%)<br>Ex-smoker (22%)<br>Non-smoker (59%)	**Very few smokers**<br>Smoker (18%)<br>Ex-smoker (22%)<br>Non-smoker (61%)	**Few smokers**<br>Smoker (22%)<br>Ex-smoker (23%)<br>Non-smoker (55%)	**Few smokers**<br>Smoker (21%)<br>Ex-smoker (28%)<br>Non-smoker (51%)	**Very few smokers**<br>Smoker (19%)<br>Ex-smoker (23%)<br>Non-smoker (58%)	**Very few smokers**<br>Smoker (13%)<br>Ex-smoker (11%)<br>Non-smoker (76%)	**Very few smokers**<br>Smoker (15%)<br>Ex-smoker (31%)<br>Non-smoker (54%)	**Ordinal**<br>1=smoker<br>2=ex-smoker<br>3=non-smoker<br>*(reversed in the profile plot)*

**Claim for compensation**	Very few have a claim<br><br>Disagree (94%)<br>Unsure (4%)<br>Agree<br>(3%)	Very few have a claim<br><br>Disagree (92%)<br>Unsure (3%)<br>Agree<br>(5%)	Very few have a claim<br><br>Disagree (92%)<br>Unsure (4%)<br>Agree<br>(4%)	Very few have a claim<br><br>Disagree (94%)<br>Unsure (1%)<br>Agree<br>(5%)	None have a claim<br><br>Disagree (100%)<br>Unsure (0%)<br>Agree<br>(0%)	Very few have a claim<br><br>Disagree (98%)<br>Unsure (0%)<br>Agree<br>(3%)	Very few have a claim<br><br>Disagree (91%)<br>Unsure (3%)<br>Agree<br>(6%)	**Ordinal**<br>0=disagree<br>1=unsure<br>2=agree
**Labelling and clinical description**	Healthy males working full-time or self-employed	Healthy females working full-time or part-time	Females with comorbidity, working full-time or part-time	Males with comorbidity, working full-time or self-employed	Healthy patients in their 30s, higher BMI, working full-time, students or unemployed	Healthy students (approx. 25 years of age), lower BMI, fewer with health insurance	Retired patients (or working part-time) with comorbidity, fewer with health insurance	
**Category size, N**	302 (33%)	191 (21%)	152 (16%)	148 (16%)	54 (6%)	40 (4%)	41 (4%)	
**Posterior probability, median**	0.99<br>(IQR=0.97-1.00)	0.99<br>(IQR=97-100)	1.00<br>(IQR=1.00-1.00)	1.00<br>(IQR=1.00-1.00)	0.99<br>(IQR=87-100)	0.99<br>(IQR=95-1.00)	1.00<br>(IQR=0.99-1.00)	
**Posterior probability less than 0.70, N**	8<br>(3%)	8<br>(4%)	1<br>(1%)	3<br>(2%)	6<br>(11%)	3<br>(8%)	0<br>(0%)	

Bold text indicates characteristic features which mainly identify the respective domain-specific patient categories
IQR = inter quartile range
Work situation: Only mentioned if percentage >5%

Figure 3. Profile plot of the domain-specific patient categories identified in the contextual factors domain by the first stage of the two-stage LCA

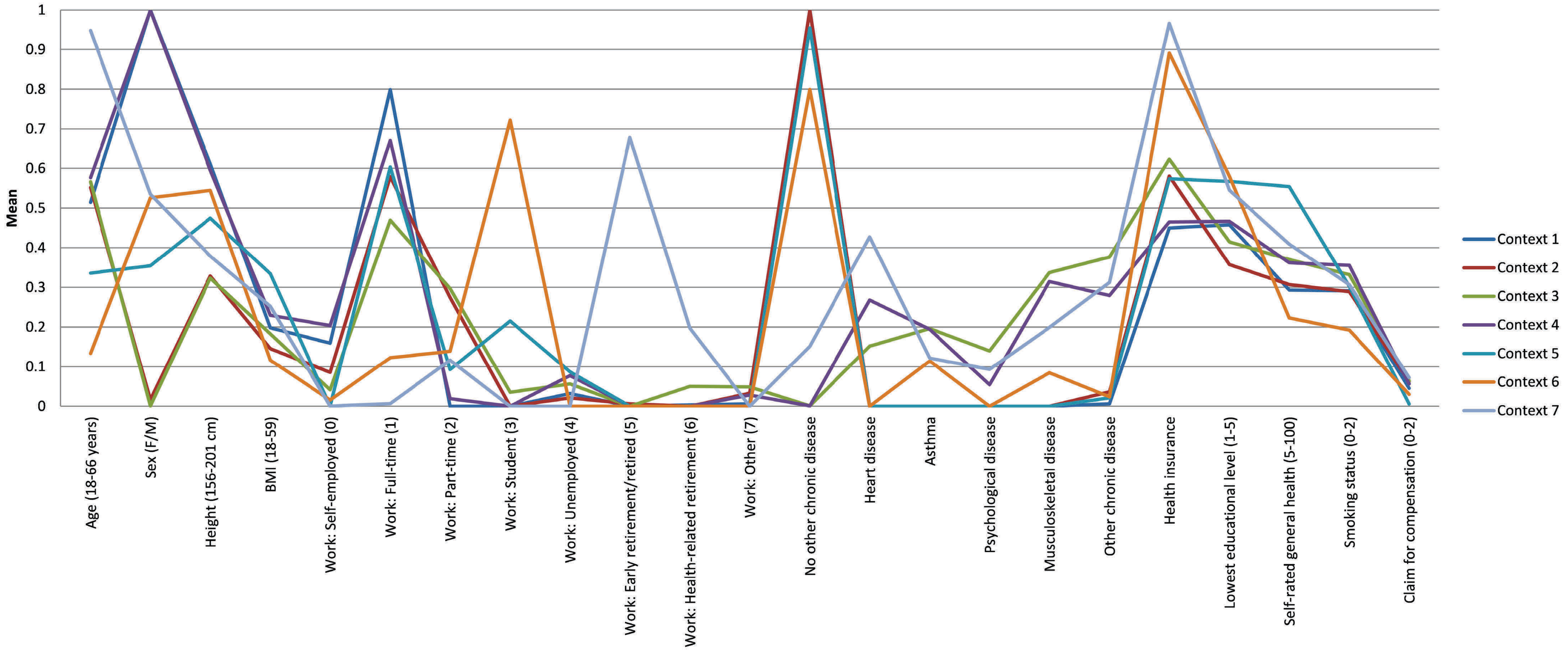

Brief conceptual description of domain-specific patient categories identified in the contextual factors domain (First stage of two-stage LCA):

Context 1: Healthy males working full-time or self-employed
Context 2: Healthy females working full-time or part-time
Context 3: Females with comorbidity, working full-time or part-time
Context 4: Males with comorbidity, working full-time or self-employed
Context 5: Healthy patients in their 30s, higher BMI, working full-time, students or unemployed
Context 6: Healthy students (approx. 25 years of age), lower BMI, fewer with health insurance
Context 7: Retired patients (or working part-time) with comorbidity, fewer with health insurance

Table 5. Descriptive presentation of the domain-specific patient categories identified in the pain domain by the first stage of the two-stage LCA

Category / Variable	Pain 1	Pain 2	Pain 3	Pain 4	Pain 5	Pain 6	Pain 7	Scale
**Previous LBP episodes**	Half have had more than three episodes<br><br>None=15%<br>1-3=38%<br>>3=47%	Half have had more than three episodes<br><br>None=19%<br>1-3=37%<br>>3=44%	Half have had more than three episodes<br><br>None=22%<br>1-3=35%<br>>3=42%	**Many have had more than three episodes**<br><br>**None=3%**<br>**1-3=21%**<br>**>3=76%**	Half have had more than three episodes<br><br>None=14%<br>1-3=40%<br>>3=46%	Half have had more than three episodes<br><br>None=25%<br>1-3=23%<br>>3=52%	Half have had more than three episodes<br><br>None=8%<br>1-3=49%<br>>3=44%	**Ordinal**<br>0=no previous episodes<br>1=1-3 previous episodes<br>2=more than 3 previous episodes
**Duration of LBP***	**0-2 weeks duration of LBP**<br><br>**0-2 weeks=89%**<br>**2-4 weeks=8%**<br>**1-3 months=3%**<br>**>3 months=<1%**	**0-2 weeks duration of LBP**<br><br>**0-2 weeks=79%**<br>**2-4 weeks=15%**<br>**1-3 months=4%**<br>**>3 months=2%**	0-4 weeks duration of LBP<br><br>0-2 weeks=65%<br>2-4 weeks=22%<br>1-3 months=9%<br>>3 months=4%	**More than 1 month duration of pain**<br><br>**0-2 weeks=13%**<br>**2-4 weeks=9%**<br>**1-3 months=31%**<br>**>3 months=46%**	0-4 weeks duration of LBP<br><br>0-2 weeks=59%<br>2-4 weeks=26%<br>1-3 months=6%<br>>3 months=9%	**More than 1 month duration of LBP**<br><br>**0-2 weeks=1%**<br>**2-4 weeks=1%**<br>**1-3 months=33%**<br>**>3 months=65%**	0-4 weeks duration of LBP<br><br>0-2 weeks=68%<br>2-4 weeks=15%<br>1-3 months=10%<br>>3 months=8%	**Ordinal**<br>1=0-2 weeks<br>2=2-4 weeks<br>3=1-3 months<br>4=>3 months
**More than 30 days of LBP last year***	Very few with more than 30 days of back pain last year<br>(7%)	Very few with more than 30 days of back pain last year<br>(6%)	Very few with more than 30 days of back pain last year<br>(5%)	**Most with more than 30 days of back pain last year**<br>**(98%)**	Very few with more than 30 days of back pain last year<br>(15%)	**All with more than 30 days of back pain last year**<br>**(100%)**	Very few with more than 30 days of back pain last year<br>(5%)	**Dichotomous**<br>0=≤ 30 days<br>1=>30 days
**LBP intensity****	**High intensity LBP**<br>**(IQR=7-9)**	**High intensity LBP**<br>**(IQR=7-8)**	Moderate intensity LBP<br>(IQR=3-6)	**High intensity LBP**<br>**(IQR=6-8)**	Moderate intensity LBP<br>(IQR=4-6)	Moderate intensity LBP<br>(IQR=4-7)	Moderate intensity LBP<br>(IQR=3-7)	**Continuous**<br>0=no pain<br>10=worst imaginable pain
**Back/leg painful almost all the time (RMDQ-11)****	Many with pain almost all the time<br>(76%)	Many with pain almost all the time<br>(77%)	**Few with pain almost all the time**<br>**(30%)**	**Most with pain almost all the time**<br>**(84%)**	Many with pain almost all the time<br>(62%)	Many with pain almost all the time<br>(61%)	Many with pain almost all the time<br>(73%)	**Dichotomous**<br>0=no<br>1=yes
**Very-extremely bothersome back pain last 2 weeks (SBT-9)****	**Most with high degree of bothersome pain**<br>**(96%)**	**Most with high degree of bothersome pain**<br>**(98%)**	**Very few with high degree of bothersome pain**<br>**(7%)**	**Most with high degree of bothersome pain**<br>**(89%)**	Few with high degree of bothersome pain<br>(24%)	Half with high degree of bothersome pain<br>(52%)	Many with high degree of bothersome pain<br>(63%)	**Dichotomous**<br>0=no-moderately bothersome pain<br>1=very-extremely bothersome pain
**LBP not dominating**	No one LBP not dominating<br>(0%)	Very few LBP not dominating<br>(4%)	Very few LBP not dominating<br>(1%)	Few LBP not dominating<br>(25%)	No one LBP not dominating<br>(0%)	No one LBP not dominating<br>(0%)	**All LBP not dominating**<br>**(100%)**	**Dichotomous**<br>0=no<br>1=yes
**Leg pain intensity*****	Most with no leg pain<br>(81%)<br>Very few with low intensity leg pain (19%)<br>None with moderate-high intensity leg pain (0%)	**Very few with no leg pain**<br>**(1%)**<br>**Half with low intensity leg pain**<br>**(53%)**<br>**Half with moderate-high intensity leg pain (46%)**	Most with no leg pain<br>(88%)<br>Very few with low intensity leg pain (12%)<br>None with moderate-high intensity leg pain (0%)	**None with no leg pain**<br>**(0%)**<br>**Few with low intensity leg pain**<br>**(22%)**<br>**Many with moderate-high intensity leg pain**<br>**(78%)**	**Very few with no leg pain**<br>**(2%)**<br>**Many with low intensity leg pain (71%)**<br>**Few with moderate-high intensity leg pain (27%)**	Half with no leg pain<br>(55%)<br>Half with low intensity leg pain<br>(45%)<br>None with moderate-high intensity leg pain (0%)	**None with no leg pain**<br>**(0%)**<br>**Few with low intensity leg pain**<br>**(25%)**<br>**Many with moderate-high intensity leg pain**<br>**(75%)**	**Ordinal**<br>0=no pain<br>1=mild pain<br>2=moderate-worst imaginable pain
**Pain has spread down leg(s) at some time last 2 weeks (SBT-1)*****	**Very few with spread of pain to leg(s)**<br>**(3%)**	Most with spread of pain to leg(s)<br>(82%)	**None with spread of pain to leg(s)**<br>**(0%)**	Most with spread of pain to leg(s)<br>(92%)	Most with spread of pain to leg(s)<br>(89%)	**Very few with spread of pain to leg(s)**<br>**(10%)**	All with spread of pain to leg(s)<br>(100%)	**Dichotomous**<br>0=no<br>1=yes
**Pain distribution*****	Most with LBP only<br><br>Only LBP (99%)<br>LBP and pain in one leg (<1%)<br>LBP and pain in both legs (<1%)<br>Only leg pain (0%)	Many with LBP only or LBP and pain in one leg<br><br>Only LBP (51%)<br>LBP and pain in one leg (37%)<br>LBP and pain in both legs (11%)	Most with LBP only<br><br>Only LBP (97%)<br>LBP and pain in one leg (3%)<br>LBP and pain in both legs (1%)<br>Only leg pain (0%)	Very few with LBP only, most with leg pain in one leg<br><br>Only LBP (18%)<br>LBP and pain in one leg (64%)<br>LBP and pain in both legs	Many with LBP only or LBP and pain in one leg<br><br>Only LBP (44%)<br>LBP and pain in one leg (53%)<br>LBP and pain in both legs (1%)	Most with LBP only<br><br>Only LBP (89%)<br>LBP and pain in one leg (11%)<br>LBP and pain in both legs (0%)<br>Only leg pain (0%)	**None with LBP only, most with LBP and pain in one leg or only leg pain**<br><br>Only LBP (0%)<br>LBP and pain in one leg (69%)	**Multistate nominal**<br>0=only low back pain<br>1=low back pain and pain in one leg<br>2=low back pain and pain in both legs<br>3=only leg pain

		Only leg pain (1%)		(14%)<br>Only leg pain (4%)	Only leg pain (1%)		LBP and pain in both legs (0%)<br>Only leg pain (31%)	
**Pain in shoulder/neck last 2 weeks (SBT-2)**	Half with shoulder/neck pain<br>(42%)	Half with shoulder/neck pain<br>(46%)	Half with shoulder/neck pain<br>(42%)	**Many with shoulder/neck pain<br>(65%)**	**Many with shoulder/neck pain<br>(62%)**	Half with shoulder/neck pain<br>(52%)	**Few with shoulder/neck pain<br>(33%)**	**Dichotomous**<br>0=no<br>1=yes
**No paraspinal pain onset**	Few with no paraspinal onset<br>(31%)	Few with no paraspinal onset<br>(38%)	Half with no paraspinal onset<br>(41%)	Half with no paraspinal onset<br>(55%)	Few with no paraspinal onset<br>(34%)	Few with no paraspinal onset<br>(36%)	Half with no paraspinal onset<br>(52%)	**Dichotomous**<br>0=no<br>1=yes
**Pain caused by physical activity (FABQ-1) ¤**	Half had pain caused by physical activity<br><br>Disagree=27%<br>Unsure=28%<br>Agree=46%	Half had pain caused by physical activity<br><br>Disagree=26%<br>Unsure=27%<br>Agree=47%	Half had pain caused by physical activity<br><br>Disagree=32%<br>Unsure=25%<br>Agree=43%	Half had pain caused by physical activity<br><br>Disagree=23%<br>Unsure=33%<br>Agree=44%	Half had pain caused by physical activity<br><br>Disagree=31%<br>Unsure=22%<br>Agree=47%	Few had pain caused by physical activity<br><br>Disagree=33%<br>Unsure=31%<br>Agree=36%	Few had pain caused by physical activity<br><br>Disagree=32%<br>Unsure=34%<br>Agree=34%	**Ordinal**<br>0=completely disagree<br>3=unsure<br>6=completely agree<br>*For description analysis, trichotomised:*<br>*0=disagree (0-2)*<br>*1=unsure (3)*<br>*2=agree (4-6)*
**Physical activity makes worse (FABQ-2) ¤**	Half get worse by physical activity<br><br>Disagree=24%<br>Unsure=20%<br>Agree=56%	Half get worse by physical activity<br><br>Disagree=25%<br>Unsure=21%<br>Agree=54%	**Few get worse by physical activity<br><br>Disagree=47%<br>Unsure=16%<br>Agree=37%**	Half get worse by physical activity<br><br>Disagree=23%<br>Unsure=18%<br>Agree=59%	Half get worse by physical activity<br><br>Disagree=43%<br>Unsure=13%<br>Agree=44%	Half get worse by physical activity<br><br>Disagree=39%<br>Unsure=13%<br>Agree=48%	Half get worse by physical activity<br><br>Disagree=37%<br>Unsure=22%<br>Agree=41%	**Ordinal**<br>0=completely disagree<br>3=unsure<br>6=completely agree<br>*For description analysis, trichotomised:*<br>*0=disagree (0-2)*<br>*1=unsure (3)*<br>*2=agree (4-6)*
**Labelling and clinical description**	**Recent LBP with high degree of back pain severity**<br><br>**More**<br>• With high intensity LBP and high degree of bothersome pain<br><br>**Less**<br>• With long duration of LBP<br>• With leg pain	**Recent LBP with high degree of back pain severity and moderate degree of leg pain severity**<br><br>**More**<br>• With high intensity LBP and high degree of bothersome pain<br>• With leg pain, low-high intensity<br><br>**Less**<br>• With long duration of LBP	**Recent LBP with low degree of back pain severity**<br><br>**Less**<br>• With pain almost all the time<br>• With leg pain<br>• Get worse with physical activity	**Persistent LBP, high degree of back and leg pain severity (and musculoskeletal comorbidity)**<br><br>**More**<br>• Previous episodes<br>• With long duration of LBP, >30 days last year<br>• With high intensity LBP and high degree of bothersome pain<br>• With pain almost all the time<br>• Moderate-high intensity leg pain<br>• With neck/shoulder pain	**Recent LBP with moderate degree of back pain severity, moderate degree of leg pain severity (and often neck/shoulder pain)**<br><br>**More**<br>• With low intensity leg pain<br>• With neck/shoulder pain	**Persistent LBP, moderate degree of back pain severity and low degree of leg pain severity**<br><br>**More**<br>• With long duration of LBP, >30 days last year<br><br>**Less**<br>• With leg pain (if present, was low intensity)	**Recent LBP, moderate degree of back pain severity, moderate to high degree of leg pain severity, non-dominating LBP**<br><br>**More**<br>• With LBP **not** dominating<br>• Moderate-high intensity leg pain<br>• With only leg pain<br><br>**Less**<br>• With neck/ shoulder pain	
**Category size, N**	252 (27%)	203 (22%)	160 (17%)	98 (11%)	89 (10%)	84 (9%)	42 (5%)	
**Posterior probability, median**	0.97<br>(IQR=80-99)	0.96<br>(IQR=84-98)	0.97<br>(IQR=82-100)	0.98<br>(IQR=88-99)	0.93<br>(IQR=75-99)	0.96<br>(IQR=85-99)	0.99<br>(IQR=84-100)	
**Posterior probability less than 0.70, N**	38<br>(15%)	29<br>(14%)	20<br>(13%)	11<br>(11%)	18<br>(20%)	8<br>(10%)	5<br>(12%)	

Bold text indicates characteristic features which mainly identify the respective domain-specific patient categories
LBP = low back pain; IQR = inter quartile range; RMDQ = Roland-Morris Disability Questionnaire; FABQ = Fear-Avoidance Beliefs Questionnaire; SBT = STarT Back Tool
Symbols and light lines: Variables which have similar scoring pattern across categories representing an overall feature:
*LBP duration
**Back pain severity
***Leg pain severity
¤Pain due to physical activity (not a diverse scoring and therefore not fulfilling the criteria for being a feature)

Figure 4. Profile plot of the domain-specific patient categories identified in the pain domain by the first stage of the two-stage LCA

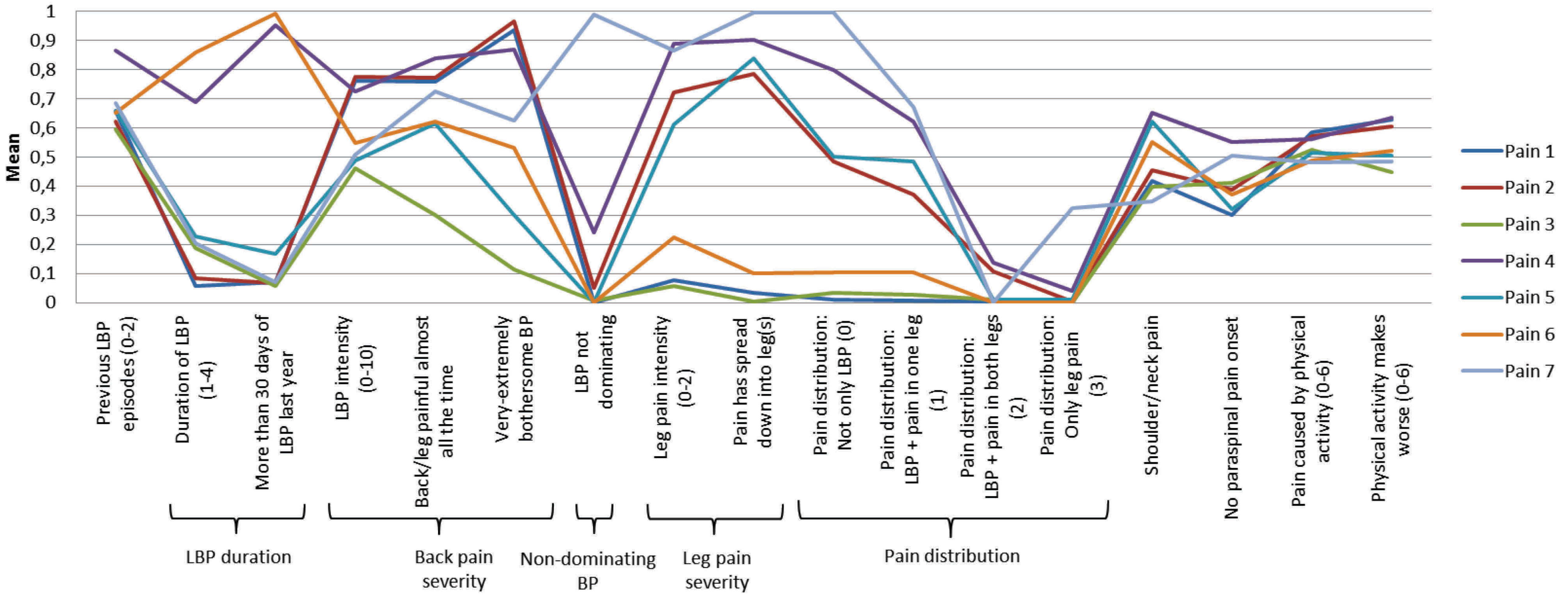

Brief conceptual description of domain-specific patient categories identified in the pain domain (first stage of two-stage LCA):

Pain 1: Recent LBP with high degree of back pain severity
Pain 2: Recent LBP with high degree of back pain severity and moderate degree of leg pain severity
Pain3: Recent LBP with low degree of back pain severity
Pain 4: Persistent LBP, high degree of back and leg pain severity
Pain 5: Recent LBP with moderate degree of back pain severity, moderate degree of leg pain severity
Pain 6: Persistent LBP, moderate degree of back pain severity and low degree of leg pain severity
Pain 7: Recent LBP, moderate degree of back pain severity, moderate-high degree of leg pain severity, non-dominating BP

LBP = low back pain

Table 6. Descriptive presentation of the domain-specific patient categories identified in the participation domain by the first stage of the two-stage LCA

Variable \ Category	Part 1	Part 2	Part 3	Part 4	Part 5	Part 6	Part 7	Scale
**Days with sick leave last month***	**Most with no sick leave (92%)**<br>Very few with 2-5 days of sick leave (8%)	**Most with no sick leave (94%)**<br>Very few with 2-5 days of sick leave (6%)	Most with no sick leave (84%)<br>Very few with 2-5 days of sick leave (16%)	Few with no sick leave (33%)<br>**Many with 2-5 days of sick leave (64%)**<br>Very few with 6-31 days of sick leave (4%)	Many with no sick leave (68%)<br>Few with 2-5 days of sick leave (30%)<br>Very few with 6-31 days of sick leave (1%)	Many with no sick leave (77%)<br>Few with 2-5 days of sick leave (20%)<br>Very few with 6-31 days of sick leave (3%)	Few with no sick leave (22%)<br>**Half with 2-5 days of sick leave (52%)**<br>**Few with 6-31 days of sick leave (26%)**	**Ordinal**<br>0=0 days<br>1=1-5 days (workweek)<br>2=6-31 days
**Decreased sexual activity (RMDQ-20)***	Few with decreased sexual activity (28%)	Few with decreased sexual activity (20%)	Few with decreased sexual activity (25%)	**Many with decreased sexual activity (79%)**	Half with decreased sexual activity (43%)	Few with decreased sexual activity (26%)	**Many with decreased sexual activity (74%)**	**Dichotomous**<br>0=no<br>1=yes
**Stay home most of the time (RMDQ-1)***	Very few stay home most of the time (9%)	None stay home most of the time (0%)	Very few stay home most of the time (2%)	**Most stay home most of the time (89%)**	Few stay home most of the time (29%)	Very few stay home most of the time (10%)	**Most stay home most of the time (86%)**	**Dichotomous**<br>0=no<br>1=yes
**Work is too heavy (FABQ-9)**	**None believe work is too heavy**<br>Disagree=100%<br>**Agree=0%**	Few believe work is too heavy<br>Disagree=74%<br>Unsure=19%<br>Agree=7%	**Half believe work is too heavy**<br>Disagree=19%<br>Unsure=23%<br>**Agree=58%**	Few believe work is too heavy<br>Disagree=71%<br>Unsure=9%<br>Agree=19%	**None believe work is too heavy**<br>Disagree=99%<br>Unsure=1%<br>**Agree=0%**	**None believe work is too heavy**<br>Disagree=100%<br>**Agree=0%**	**Half believe work is too heavy**<br>Disagree=29%<br>Unsure=13%<br>**Agree=58%**	**Ordinal**<br>0=disagree<br>1=unsure<br>2=agree
**Work makes/would make pain worse (FABQ-10)**	**None believe work makes pain worse**<br>Disagree=100%<br>**Agree=0%**	Few believe work makes pain worse<br>Disagree=22%<br>**Unsure=54%**<br>Agree=24%	**Most believe work makes pain worse**<br>Disagree=2%<br>Unsure=12%<br>**Agree=86%**	Few believe work makes pain worse<br>Disagree=41%<br>Unsure=36%<br>Agree=24%	Half believe work makes pain worse<br>Disagree=32%<br>Unsure=26%<br>Agree=42%	**None believe work makes pain worse**<br>Disagree=94%<br>Unsure=6%<br>Agree=0%	**Most believe work makes pain worse**<br>Disagree=0%<br>**Unsure=11%**<br>**Agree=89%**	**Ordinal**<br>0=disagree<br>1=unsure<br>2=agree
**Work-aggravated pain (FABQ-7)**	**None believe work aggravated pain**<br>Disagree=93%<br>Unsure=7%<br>**Agree=0%**	Few believe work aggravated pain<br>Disagree=35%<br>**Unsure=51%**<br>Agree=14%	**Most believe work aggravated pain**<br>Disagree=0%<br>Unsure=17%<br>**Agree=83%**	Very few believe work aggravated pain<br>Disagree=65%<br>Unsure=29%<br>Agree=6%	**Most believe work aggravated pain**<br>Disagree=0%<br>Unsure=8%<br>**Agree=92%**	Few believe work aggravated pain<br>Disagree=44%<br>Unsure=31%<br>Agree=24%	**Most believe work aggravated pain**<br>Disagree=0%<br>Unsure=13%<br>**Agree=88%**	**Ordinal**<br>0=disagree<br>1=unsure<br>2=agree
**Pain caused by work/accident at work (FABQ-6)**	**None believe pain caused by work/accident at work**<br>Disagree=100%<br>Agree=0%	Very few believe pain caused by work/accident at work<br>Disagree=69%<br>**Unsure=28%**<br>Agree=3%	Many believe pain caused by work/accident at work<br>Disagree=20%<br>Unsure=18%<br>Agree=61%	Very few believe pain caused by work/accident at work<br>Disagree=74%<br>Unsure=20%<br>Agree=6%	Few believe pain caused by work/accident at work<br>Disagree=40%<br>Unsure=29%<br>Agree=31%	**Most believe pain caused by work/accident at work**<br>Disagree=0%<br>Unsure=10%<br>**Agree=90%**	**Many believe pain caused by work/accident at work**<br>Disagree=13%<br>Unsure=14%<br>**Agree=73%**	**Ordinal**<br>0=disagree<br>1=unsure<br>2=agree
**Physical workload**	Sitting and walking at work<br>**Few only sit at work (30%)**<br>**Half sit and walk at work (50%)**<br>Very few have light physical load (15%)<br>Very few have heavy physical load (4%)	Sitting and walking / low physical load<br>Few only sit at work (20%)<br>Half sit and walk at work (41%)<br>Few have light physical load (24%)<br>Very few have heavy physical load (16%)	Low-high physical load<br>Very few only sit at work (5%)<br>Very few sit and walk at work (11%)<br>**Few have light physical load (23%)**<br>**Many have heavy physical load (61%)**	Low physical load (unspecific)<br>Very few only sit at work (17%)<br>Few sit and walk at work (27%)<br>Few have light physical load (30%)<br>Few have heavy physical load (26%)	Sit at work<br>**Many only sit at work (77%)**<br>Few sit and walk at work (21%)<br>Very few have light physical load (1%)<br>None have heavy physical load (0%)	Sitting and walking / low physical load<br>Very few only sit at work (3%)<br>**Few sit and walk at work (32%)**<br>**Few have light physical load (36%)**<br>**Few have heavy physical load (29%)**	Low-high physical load<br>None only sit at work (0%)<br>Very few sit and walk at work (9%)<br>**Few have light physical load (29%)**<br>**Many have heavy physical load (62%)**	**Ordinal**<br>1=sitting<br>2=sitting and walking<br>3=light physical load<br>4=heavy physical load

**Labelling and clinical description**	**'Copers'**<br>**Very few work and social participation limitations, low degree of physical workload**<br><br>**Less**<br>• With sick leave<br>• Believe work is too heavy<br>• Believe work makes pain worse<br>• Believe work aggravated pain<br>• Believe pain caused by work<br>• With light-heavy physical workload	**Very few social participation limitations, unsure if work aggravated/makes pain worse whichever degree of physical workload**<br><br>**Less**<br>• With sick leave	**'Too heavy work makes pain worse'**<br><br>**Very few social participation limitations, work is too heavy, work aggravated/makes pain worse, pain caused by or at work, high degree of physical workload**<br><br>**More**<br>• Believe work is too heavy<br>• Believe work makes pain worse<br>• Believe work aggravated pain<br>• With light-heavy physical workload	**'Social participation limitations'**<br><br>**High degree of social participation limitations, but low degree of work limitations whichever degree of physical workload**<br><br>**More**<br>• With sick leave 2-5 days<br>• With decreased sexual activity<br>• Stay home most of the time	**'Sitting at work makes pain worse'**<br><br>**Moderate degree of social participation limitations, work aggravated/makes pain worse, very low degree of physical workload**<br><br>**More**<br>• Sit at work<br>• Believe work aggravated pain<br><br>**Less**<br>• Believe work is too heavy	**'Pain caused by work'**<br><br>**Low degree of social participation limitations, pain caused by work, none with very low degree of physical workload**<br><br>**Less**<br>• Believe work is too heavy<br>• Believe work makes worse<br>• Believe pain caused by work<br>• Only sit at work	**'Severe participation limitations'**<br><br>**High degree of social participation limitations, work is too heavy, work aggravated/makes pain worse, pain caused by or at work, high degree of physical workload**<br><br>**More**<br>• With sick leave 2-5 days or 6-31 days<br>• With decreased sexual activity<br>• Stay home most of the time<br>• Believe work is too heavy<br>• Believe work makes pain worse<br>• Believe work aggravated pain<br>• Believe pain caused by work<br>• With low-high physical work load	
**Category size, N**	348 (38%)	154 (17%)	128 (14%)	93 (10%)	77 (8%)	70 (8%)	58 (6%)	
**Posterior probability, median**	0.94<br>(IQR=83-95)	0.67<br>(IQR=54-85)	0.87<br>(IQR=70-96)	0.83<br>(IQR=68-95)	0.79<br>(IQR=62-88)	0.84<br>(IQR=72-91)	0.91<br>(IQR=68-99)	
**Posterior probability less than 0.70, N**	58<br>(17%)	85<br>(55%)	32<br>(25%)	27<br>(29%)	25<br>(32%)	17<br>(24%)	15<br>(26%)	

IQR = interquartile range; FABQ-work = Fear-Avoidance Beliefs Questionnaire, work subscale
Symbols and light lines: Variables which have similar scoring pattern across categories representing an overall feature:
*Social participation limitations

Figure 5. Profile plot of the domain-specific patient categories identified in the participation domain by the first stage of the two-stage LCA

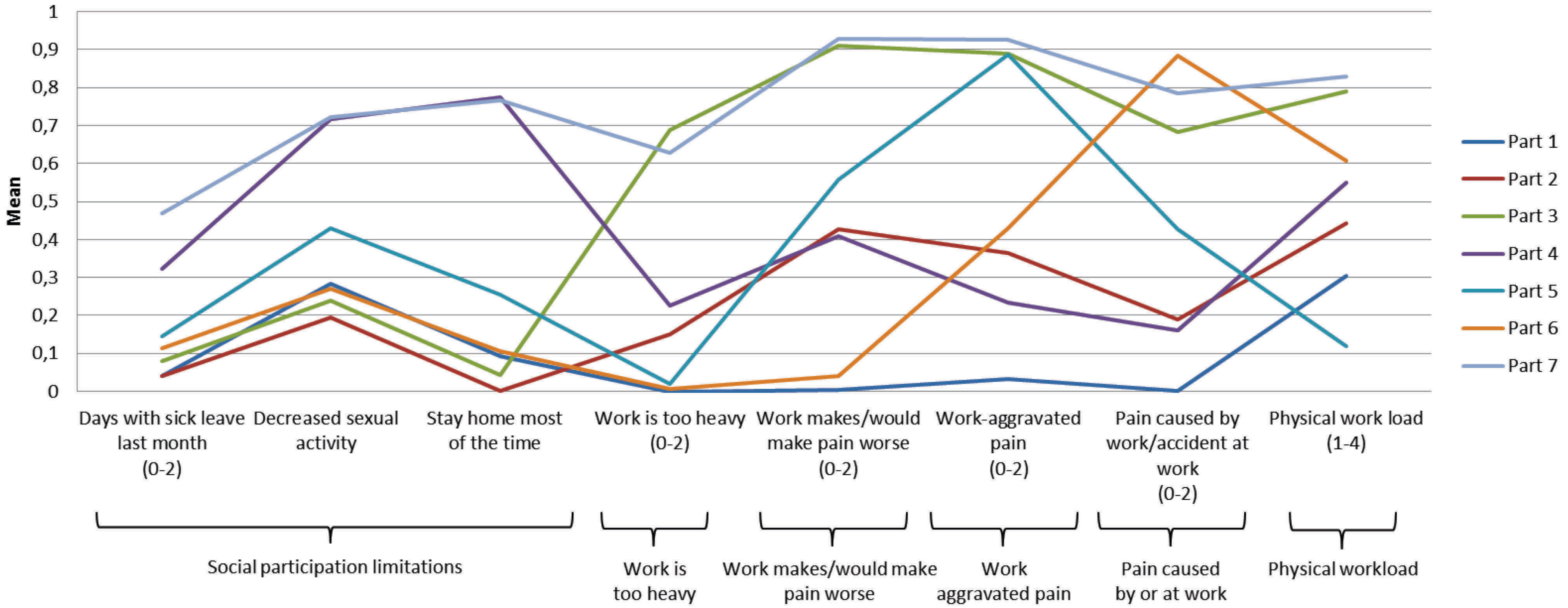

Brief conceptual description of domain-specific patient categories identified in the participation domain (first stage of two-stage LCA):

Part 1: Very low work and social participation limitations, low degree of physical workload
Part 2: Very low social participation limitations, unsure if work makes/would make worse or aggravated pain, whichever degree of physical workload
Part 3: Very low social participation limitations, work is too heavy, work aggravated/makes pain worse, pain caused by or at work, high degree of physical workload
Part 4: High degree of social participation limitations, but low degree of work limitations whichever degree of physical workload
Part 5: Moderate degree of social participation limitation, work aggravated/makes pain worse, very low degree of physical workload
Part 6: Low degree of social participation limitations, pain caused by work, none with very low degree of physical workload
Part 7: High degree of social participation limitations, work is too heavy work, work aggravated/makes pain worse, pain caused by or at work, high degree of physical workload

Table 7. Descriptive presentation of the domain-specific patient categories identified in the physical impairment domain by the first stage of the two-stage LCA

Feature \ Category	Phy. imp. 1	Phy. imp. 2	Phy. imp. 3	Phy. imp. 4	Phy. imp. 5	Phy. imp. 6	Scale
**Pain on flexion (ROM)**	**Half with no pain (48%)**<br>**Half with back pain (50%)**<br>Very few with leg pain w/wo back pain (2%)	**Half with no pain (40%)**<br>**Half with back pain (58%)**<br>Very few with leg pain w/wo back pain (1%)	Few with no pain (30%)<br>**Many with back pain (65%)**<br>Very few with leg pain w/wo back pain (5%)	Few with no pain (25%)<br>**Many with back pain (70%)**<br>Very few with leg pain w/wo back pain (5%)	Very few with no pain (8%)<br>**Most with back pain (92%)**<br>None with leg pain w/wo back pain (0%)	Very few with no pain (14%)<br>Few with back pain (21%)<br>**Many with leg pain w/wo back pain (65%)**	**Multistate nominal**<br>0=no pain<br>1=back pain<br>2=leg pain w/wo back pain
**Pain on extension (ROM)**	**Half with no pain (46%)**<br>**Half with back pain (54%)**<br>None with leg pain w/wo back pain (0%)	Few with no pain (28%)<br>**Many with back pain (70%)**<br>Very few with leg pain w/wo back pain (2%)	**Half with no pain (42%)**<br>**Half with back pain (58%)**<br>None with leg pain w/wo back pain (0%)	**Half with no pain (42%)**<br>**Half with back pain (58%)**<br>None with leg pain w/wo back pain (0%)	Very few with no pain (2%)<br>**Most with back pain (96%)**<br>Very few with leg pain w/wo back pain (2%)	Few with no pain (33%)<br>Few with back pain (27%)<br>**Half with leg pain w/wo back pain (40%)**	**Multistate nominal**<br>0=no pain<br>1=back pain<br>2=leg pain w/wo back pain
**Pain on right side glide (ROM)**	**Many with no pain (70%)**<br>Few with back pain (30%)<br>None with leg pain w/wo back pain (0%)	**Half with no pain (50%)**<br>**Half with back pain (48%)**<br>Very few with leg pain w/wo back pain (1%)	**Many with no pain (60%)**<br>**Few with back pain (39%)**<br>Very few with leg pain w/wo back pain (1%)	**Half with no pain (55%)**<br>**Half with back pain (45%)**<br>None with leg pain w/wo back pain (0%)	Very few with no pain (10%)<br>**Most with back pain (90%)**<br>None with leg pain w/wo back pain (0%)	**Half with no pain (52%)**<br>Very few with back pain (17%)<br>**Few with leg pain w/wo back pain (32%)**	**Multistate nominal**<br>0=no pain<br>1=back pain<br>2=leg pain w/wo back pain
**Pain on left side glide (ROM)**	**Many with no pain (73%)**<br>Few with back pain (27%)<br>None with leg pain w/wo back pain (0%)	**Half with no pain (43%)**<br>**Half with back pain (57%)**<br>None with leg pain w/wo back pain (0%)	**Many with no pain (70%)**<br>Few with back pain (30%)<br>None with leg pain w/wo back pain (0%)	**Many with no pain (60%)**<br>**Few with back pain (39%)**<br>Very few with leg pain w/wo back pain (1%)	Very few with no pain (10%)<br>**Most with back pain (88%)**<br>Very few with leg pain w/wo back pain (2%)	Few with no pain (36%)<br>Few with back pain (22%)<br>**Half with leg pain w/wo back pain (42%)**	**Multistate nominal**<br>0=no pain<br>1=back pain<br>2=leg pain w/wo back pain
**Pain on right rotation (ROM)**	**Most with no pain (90%)**<br>Very few with back pain (9%)<br>Very few with leg pain w/wo back pain (1%)	**Many with no pain (73%)**<br>Few with back pain (27%)<br>None with leg pain w/wo back pain (0%)	**Most with no pain (80%)**<br>Few with back pain (20%)<br>None with leg pain w/wo back pain (0%)	**Most with no pain (94%)**<br>Very few with back pain (6%)<br>None with leg pain w/wo back pain (0%)	Very few with no pain (1%)<br>**Most with back pain (99%)**<br>None with leg pain w/wo back pain (0%)	**Many with no pain (69%)**<br>Very few with back pain (15%)<br>Very few with leg pain w/wo back pain (15%)	**Multistate nominal**<br>0=no pain<br>1=back pain<br>2=leg pain w/wo back pain
**Pain on left rotation (ROM)**	**Most with no pain (93%)**<br>Very few with back pain (7%)<br>None with leg pain w/wo back pain (0%)	**Many with no pain (74%)**<br>Few with back pain (26%)<br>None with leg pain w/wo back pain (0%)	**Most with no pain (80%)**<br>Few with back pain (20%)<br>None with leg pain w/wo back pain (0%)	**Most with no pain (81%)**<br>Very few with back pain (19%)<br>None with leg pain w/wo back pain (0%)	Very few with no pain (3%)<br>**Most with back pain (97%)**<br>None with leg pain w/wo back pain (0%)	**Many with no pain (60%)**<br>Very few with back pain (17%)<br>**Few with leg pain w/wo back pain (22%)**	**Multistate nominal**<br>0=no pain<br>1=back pain<br>2=leg pain w/wo back pain
**Diagnosis based on 'McKenzie testing'**	None with reducible disc (0%)<br>None with partly reducible disc (0%)<br>Very few with non-reducible disc (10%)<br>**Few with dysfunction (25%)**	**Few with reducible disc (26%)**<br>Very few with partly reducible disc (15%)<br>Very few with non-reducible disc (4%)<br>Very few with dysfunction (4%)	**All with reducible disc (100%)**<br>None with partly reducible disc (0%)<br>None with non-reducible disc (0%)<br>None with dysfunction (0%)	None with reducible disc (0%)<br>**All with partly reducible disc (100%)**<br>None with non-reducible disc (0%)<br>None with dysfunction (0%)	Very few with reducible disc (9%)<br>**Few with partly reducible disc (29%)**<br>Very few with non-reducible disc (2%)<br>**Very few with dysfunction (16%)**	**Very few with reducible disc (16%)**<br>**Few with partly reducible disc (30%)**<br>**Few with non-reducible disc (25%)**<br>None with dysfunction (0%)	**Dichotomous (4 items)**<br>0=no<br>1=yes<br>Reducible disc<br>Partly reducible disc<br>Non-reducible disc<br>Dysfunction
**Positive neurological examination**	Very few with indication of herniated disc (1% right, 1% left)<br>Very few with affected muscular strength (1%), sensibility (2%) or reflexes (2%)	Very few with indication of herniated disc (4% right, 2% left)<br>Very few with affected muscular strength (1%), sensibility (7%) or reflexes (7%)	Very few with indication of herniated disc (7% right, 7% left)<br>Very few with affected muscular strength (2%), sensibility (6%) or reflexes (5%)	Very few with indication of herniated disc (0% right, 2% left)<br>Very few with affected muscular strength (2%), sensibility (1%) or reflexes (5%)	Very few with indication of herniated disc (7% right, 5% left)<br>Very few with affected muscular strength (4%), sensibility (3%) or reflexes (6%)	**Few (36% right) half (42% left) with indication of herniated disc**<br>**Few with affected muscular strength (26%) and sensibility (27%), and half with reflexes (42%)**	**Dichotomous (5 items)**<br>0=no<br>1=yes<br>Indication of herniated disc, right side<br>Indication of herniated disc, left side<br>Affected muscular strength<br>Affected sensibility<br>Affected deep tendon reflexes
**Positive sacroiliac joint tests**	Very few with positive tests:<br>Separation (2%)<br>Thigh thrust (10%)<br>Gaenslen's (7%)	**Many with positive tests:**<br>**Separation (42%)**<br>**Thigh thrust (77%)**<br>**Gaenslen's (70%)**	Very few with positive tests:<br>Separation (0%)<br>Thigh thrust (5%)<br>Gaenslen's (9%)	Very few with positive tests:<br>Separation (5%)<br>Thigh thrust (10%)<br>Gaenslen's (6%)	Few with positive tests:<br>Separation (6%)<br>Thigh thrust (26%)<br>Gaenslen's (16%)	Few with positive tests:<br>Separation (10%)<br>Thigh thrust (27%)<br>Gaenslen's (20%)	**Dichotomous (5 items)**<br>0=negative<br>1=positive

	Compression (2%)<br>Sacral thrust (20%)	**Compression (63%)<br>Sacral thrust (83%)**	Compression (4%)<br>Sacral thrust (14%)	Compression (3%)<br>Sacral thrust (17%)	Compression (9%)<br>Sacral thrust (36%)	Compression (18%)<br>Sacral thrust (29%)	Separation test<br>Thigh thrust<br>Gaenslen's test,<br>Compression test,<br>Sacral thrust
**Pain on combined extension/rotation**	Half with pain on extension/rotation (44%)	Half with pain on extension/rotation (43%)	Half with pain on extension/rotation (57%)	**Many with pain on extension/rotation (63%)**	Very few with pain on extension/rotation (13%)	Half with pain on extension/rotation (58%)	**Dichotomous**<br>0=no<br>1=yes
**Trigger points**	Few with replication of pain by trigger point (25%)	**Half with replication of pain by trigger point (52%)**	Very few with replication of pain by trigger point (18%)	Very few with replication of pain by trigger point (9%)	Very few with replication of pain by trigger point (11%)	Few with replication of pain by trigger point (38%)	**Dichotomous**<br>0=no<br>1=yes
**Pain on muscle palpation**	Half with replication of pain on muscle palpation (56%)	**Most with replication of pain on muscle palpation (80%)**	Half with replication of pain on muscle palpation (55%)	Few with replication of pain on muscle palpation (36%)	Half with replication of pain on muscle palpation (44%)	**Many with replication of pain on muscle palpation (77%)**	**Dichotomous**<br>0=no<br>1=yes
**Painful muscle groups**	**Half with painful back muscles (46%)**<br>Few with painful buttock and leg muscles (27%)<br>Few with both painful back and buttock/leg muscles (28%)	Very few with painful back muscles (18%)<br>**Half with painful buttock and leg muscles (55%)**<br>Few with both painful back and buttock/leg muscles (27%)	Few with painful back muscles (37%)<br>Few with painful buttock and leg muscles (39%)<br>Few with both painful back and buttock/leg muscles (24%)	Few with painful back muscles (37%)<br>Few with painful buttock and leg muscles (31%)<br>Few with both painful back and buttock/leg muscles (31%)	**Half with painful back muscles (50%)**<br>Few with painful buttock and leg muscles (27%)<br>Few with both painful back and buttock/leg muscles (23%)	Few with painful back muscles (25%)<br>**Half with painful buttock and leg muscles (53%)**<br>Few with both painful back and buttock/leg muscles (22%)	**Multistate nominal**<br>0=back muscles<br>1=buttock and leg muscles<br>2=back and buttock/leg muscles
**Acute lateral shift**	Very few with acute lateral shift (4%)	Very few with acute lateral shift (8%)	Very few with acute lateral shift (13%)	Very few with acute lateral shift (16%)	Very few with acute lateral shift (16%)	Very few with acute lateral shift (12%)	**Dichotomous**<br>0=no<br>1=yes
**Acute flexion deformity**	Very few with acute flexion deformity (5%)	Very few with acute flexion deformity (2%)	Very few with acute flexion deformity (3%)	Very few with acute flexion deformity (7%)	Very few with acute flexion deformity (7%)	Very few with acute flexion deformity (7%)	**Dichotomous**<br>0=no<br>1=yes
**Labelling and clinical description**	**Mainly back pain on flexion and extension, no leg pain**<br><br>**More**<br>• with no pain in most directions<br>• with the diagnosis: dysfunction<br>• with painful back muscles on palpation	**Mainly back pain on flexion, extension and side glide, no leg pain, but SI joint pain, trigger points and painful buttock/leg muscles**<br><br>**More**<br>• with the diagnosis: Reducible disc<br>• with positive SI joint tests<br>• with painful muscle palpation/trigger points	**Diagnosis: reducible disc; mainly back pain on flexion, extension and side glide, no leg pain**<br><br>**More**<br>• with the diagnosis: Reducible disc	**Diagnosis: partly reducible disc, mainly back pain on flexion, extension and side glide, no leg pain**<br><br>**More**<br>• with the diagnosis: Partly reducible disc<br>• with pain on extension/rotation	**Back pain on AROM in all directions, painful back muscles**<br><br>**More**<br>• with painful back muscles	**Mainly leg pain on flexion, extension and side glide, neurological signs, trigger points and painful buttock/leg muscles**<br><br>**More**<br>• with leg pain on ROM<br>• with a 'disc-diagnosis'<br>• with neurological signs<br>• with painful buttock and leg muscles	
**Category size, N**	264 (28%)	180 (19%)	147 (16%)	138 (15%)	126 (14%)	73 (8%)	
**Posterior probability, median (IQR)**	0.97<br>(0.88-0.99)	0.99<br>(0.89-1.00)	0.99<br>(0.91-1.00)	0.99<br>(0.95-1.00)	0.88<br>(0.73-0.99)	1.00<br>(0.99-1.00)	
**Posterior probability less than 0.70, N**	28<br>(11%)	22<br>(12%)	14<br>(10%)	5<br>(4%)	27<br>(2%)	6<br>(8%)	

Bold text indicates characteristic features which mainly identify the respective domain-specific patient categories
Symbols and light lines: Variables which have similar scoring pattern across categories representing an overall feature:
Phy.imp. = physical impairment, IQR=inter quartile range, ROM = range of motion, w/wo = with or without

Figure 6. Profile plot of the domain-specific patient categories identified in the physical impairment domain by the first stage of the two-stage LCA

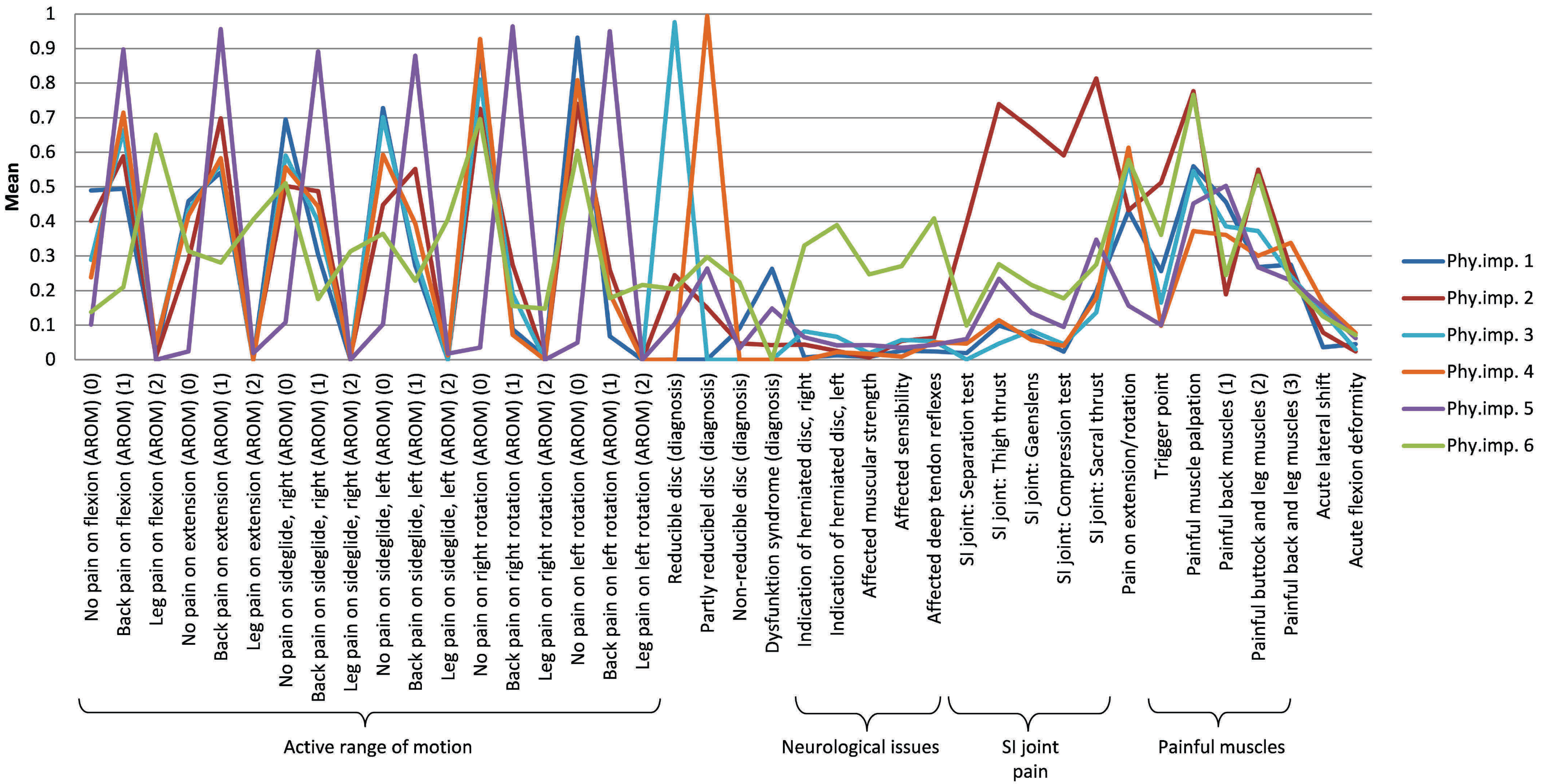

Brief conceptual description of domain-specific patient categories identified in the physical impairment domain (first stage of two-stage LCA):

Phy. imp. 1: Mainly back pain on flexion and extension, no leg pain

Phy. imp. 2: Mainly back pain on flexion, extension and side glide, no leg pain, *SI joint pain*, trigger points and painful buttock/leg muscles

Phy. imp. 3: Mainly back pain on flexion, extension and side glide, no leg pain, *diagnosis: reducible disc*

Phy. imp. 4: Mainly back pain on flexion, extension and side glide, no leg pain, *diagnosis: partly reducible disc*

Phy. imp. 5: Back pain on AROM in all directions, painful back muscles

Phy. imp. 6: Mainly leg pain on flexion, extension and side glide, neurological signs, trigger points and painful buttock/leg muscles

Table 8: Descriptive presentation of the domain-specific patient categories identified in the psychology domain by the first stage of the two-stage LCA

Category / Variable	Psych 1	Psych 2	Psych 3	Psych 4	Psych 5	Psych 6	Psych 7	Psych 8	Scale
**Treatment not essential**	**Very few believe treatment is not essential to reduce pain<br>(IQR=0-0)**	Half believe treatment is not essential to reduce pain<br>(IQR=2-8)	Half believe treatment is not essential to reduce pain<br>(IQR=2-5)	Half believe treatment is not essential to reduce pain<br>(IQR=2-8)	**Very few believe treatment is not essential to reduce pain<br>(IQR=0-0)**	Half believe treatment is not essential to reduce pain<br>(IQR=2-7)	Few believe treatment is not essential to reduce pain<br>(IQR=1-6)	**Many believe treatment is not essential to reduce pain<br>(IQR=3-10)**	**Continuous**<br>0=completely disagree<br>10=completely agree
**Sleep less well (RMDQ-15)***	Many sleep less well<br>(61%)	Many sleep less well<br>(72%)	**Few sleep less well<br>(28%)**	**None sleep less well<br>(0%)**	Many sleep less well<br>(78%)	**All sleep less well<br>(100%)**	Many sleep less well<br>(69%)	Many sleep less well<br>(74%)	**Dichotomous**<br>0=no<br>1=yes
**Had trouble sleeping at night (MDI-9)***	Very few had trouble sleeping<br>(IQR=0-1)	Few had trouble sleeping<br>(IQR=1-3)	Very few had trouble sleeping<br>(IQR=0-1)	Very few had trouble sleeping<br>(IQR=0-0)	**Half had trouble sleeping<br>(IQR=1-4)**	**Half had trouble sleeping<br>(IQR=1-3)**	**Many had trouble sleeping<br>(IQR=2-4)**	Few had trouble sleeping<br>(IQR=0-2)	**Ordinal**<br>0=at no time<br>5=all the time
**Able to decrease pain****	Moderately able to decrease pain<br>(IQR=2-7)	Moderately able to decrease pain<br>(IQR=3-6)	Highly able to decrease pain<br>(IQR=1-4)	Highly able to decrease pain<br>(IQR=2-5)	Moderately able to decrease pain<br>(IQR=2-7)	Highly able to decrease pain<br>(IQR=2-5)	Moderately able to decrease pain<br>(IQR=3-6)	**Moderately able to decrease pain<br>(IQR=3.5-7)**	**Continuous**<br>0=Completely<br>10=Not at all
**Work might harm back (FABQ-11)****	Few believe work will harm<br><br>Disagree=56%<br>Unsure=17%<br>Agree=28%	**Half believe work will harm**<br><br>Disagree=24%<br>Unsure=21%<br>Agree=55%	**Very few believe work will harm**<br><br>Disagree=87%<br>Unsure=8%<br>Agree=5%	Few believe work will harm<br><br>Disagree=54%<br>Unsure=19%<br>Agree=27%	Few believe work will harm<br><br>Disagree=35%<br>Unsure=28%<br>Agree=37%	**Very few believe work will harm**<br><br>Disagree=72%<br>Unsure=9%<br>Agree=18%	**Half believe work will harm**<br><br>Disagree=24%<br>Unsure=28%<br>Agree=48%	Few believe work will harm<br><br>Disagree=34%<br>Unsure=30%<br>Agree=36%	**Ordinal[ǂ]**<br>0=completely disagree<br>3=unsure<br>6=completely agree
**Should not do normal work with present pain (FABQ-12)****	Few believe they should not do normal work<br><br>Disagree=52%<br>Unsure=21%<br>Agree=27%	**Half believe they should not do normal work**<br><br>Disagree=29%<br>Unsure=18%<br>Agree=53%	**Very few believe they should not do normal work**<br><br>Disagree=99%<br>Unsure=1%<br>Agree=0%	**Very few believe they should not do normal work**<br><br>Disagree=73%<br>Unsure=17%<br>Agree=11%	Few believe they should not do normal work<br><br>Disagree=43%<br>Unsure=22%<br>Agree=35%	**Very few believe they should not do normal work**<br><br>Disagree=90%<br>Unsure=7%<br>Agree=3%	Few believe they should not do normal work<br><br>Disagree=37%<br>Unsure=24%<br>Agree=39%	**Half believe they should not do normal work**<br><br>Disagree=23%<br>Unsure=23%<br>Agree=55%	**Ordinal[ǂ]**<br>0=completely disagree<br>3=unsure<br>6=completely agree
**Cannot work till pain is treated (FABQ-14)****	Few cannot work<br><br>Disagree=56%<br>Unsure=10%<br>Agree=34%	**Half cannot work**<br><br>Disagree=36%<br>Unsure=20%<br>Agree=44%	**None cannot work**<br><br>Disagree=100%<br>Unsure=0%<br>Agree=0%	**Very few cannot work**<br><br>Disagree=83%<br>Unsure=8%<br>Agree=9%	Few cannot work<br><br>Disagree=60%<br>Unsure=8%<br>Agree=33%	**Very few cannot work**<br><br>Disagree=92%<br>Unsure=3%<br>Agree=5%	**Half cannot work**<br><br>Disagree=43%<br>Unsure=9%<br>Agree=48%	**Half cannot work**<br><br>Disagree=36%<br>Unsure=7%<br>Agree=57%	**Ordinal[ǂ]**<br>0=completely disagree<br>3=unsure<br>6=completely agree
**Feel socially isolated**	**Very few feel socially isolated<br>(7%)**	Half feel socially isolated<br>(53%)	**Very few feel socially isolated<br>(6%)**	Few feel socially isolated<br>(37%)	Few feel socially isolated<br>(30%)	Few feel socially isolated<br>(21%)	**Many feel socially isolated<br>(76%)**	**Very few feel socially isolated<br>(2%)**	**Dichotomous**<br>0=Not at all isolated<br>1=little to quite isolated
**Not enjoyed things used to enjoy (SBT-8) ¤**	Few have not enjoyed usual things<br>(29%)	Many have not enjoyed usual things<br>(61%)	**Very few have not enjoyed usual things<br>(6%)**	Few have not enjoyed usual things<br>(24%)	Many have not enjoyed usual things<br>(62%)	Half have not enjoyed usual things<br>(41%)	**Most have not enjoyed usual things<br>(83%)**	Half have not enjoyed usual things<br>(44%)	Dichotomous<br>0=disagree<br>1=agree
**Negative recovery belief ¤**	**Very few with negative recovery belief<br>(18%)**	Few with negative recovery belief<br>(38%)	**Very few with negative recovery belief<br>(15%)**	Few with negative recovery belief<br>(33%)	Few with negative recovery belief<br>(33%)	Few with negative recovery belief<br>(28%)	**Half with negative recovery belief<br>(53%)**	**Very few with negative recovery belief<br>(15%)**	**Dichotomous**<br>0=Likely to recover<br>1=Unsure-Not at all likely
**Worrying thoughts a lot of the time (SBT-6) ¤**	**Very few worried<br>(9%)**	Half worried<br>(42%)	**Very few worried<br>(2%)**	**Very few worried<br>(8%)**	Half worried<br>(47%)	Few worried<br>(24%)	**Most worried<br>(85%)**	**Very few worried<br>(4%)**	Dichotomous<br>0=disagree<br>1=agree
**Lost interest in daily activities (MDI-2) ¤**	Very few have lost interest all the time, half some of the time<br><br>At no time=65%	Few have lost interest all the time, half some of the time<br><br>At no time=17%	**None have lost interest all the time, very few some of the time**	Very few have lost interest all the time, half some of the time<br><br>At no time=35%	Half have lost interest all the time, half some of the time<br><br>At no time=9%	Very few have lost interest all the time, half some of the time<br><br>At no time=50%	**Most have lost interest all the time, very few some of the time**	**None have lost interest all the time, very few some of the time**	**Ordinal**<br>0=at no time<br>1=some of the time<br>2=slightly-all the time

	Some of the time=31%<br>Slightly-all the time=4%	Some of the time=55%<br>Slightly-all the time=28%	At no time=92%<br>Some of the time=8%<br>Slightly-all the time=0%	Some of the time=54%<br>Slightly-all the time=11%	Some of the time=44%<br>Slightly-all the time=47%	Some of the time=47%<br>Slightly-all the time=4%	At no time=2%<br>Some of the time=9%<br>Slightly-all the time=89%	At no time=92%<br>Some of the time=8%<br>Slightly-all the time=0%	
**Felt very restless/subdued/slowed down (MDI-8)** ¤	Very few have felt restless all the time, half some of the time<br><br>At no time=47%<br>Some of the time=46%<br>Slightly-all the time=7%	Half have felt restless all the time, half some of the time<br><br>At no time=4%<br>Some of the time=50%<br>Slightly-all the time=46%	**Very few have felt restless all the time, few some of the time**<br><br>At no time=71%<br>Some of the time=28%<br>Slightly-all the time=1%	Very few have felt restless all the time, many some of the time<br><br>At no time=25%<br>Some of the time=66%<br>Slightly-all the time=8%	Half have felt restless all the time, few some of the time<br><br>At no time=2%<br>Some of the time=39%<br>Slightly-all the time=58%	Very few have felt restless all the time, many some of the time<br><br>At no time=21%<br>Some of the time=62%<br>Slightly-all the time=17%	**Most have felt restless all the time, very few some of the time**<br><br>At no time=0%<br>Some of the time=5%<br>Slightly-all the time=95%	**Very few have felt restless all the time, few some of the time**<br><br>At no time=65%<br>Some of the time=33%<br>Slightly-all the time=2%	**Ordinal**<br>0=at no time<br>1=some of the time<br>2=slightly-all the time
**Had a bad conscience (MDI-5)** ¤	Very few had bad conscience all the time or some of the time<br><br>At no time=77%<br>Some of the time=19%<br>Slightly-all the time=4%	Few had bad conscience all the time or some of the time<br><br>At no time=35%<br>Some of the time=37%<br>Slightly-all the time=28%	**None had bad conscience all the time and very few some of the time**<br><br>At no time=92%<br>Some of the time=8%<br>Slightly-all the time=0%	Very few had bad conscience all the time and few some of the time<br><br>At no time=60%<br>Some of the time=37%<br>Slightly-all the time=3%	Half had bad conscience all the time and few some of the time<br><br>At no time=25%<br>Some of the time=32%<br>Slightly-all the time=43%	Very few had bad conscience all the time and half some of the time<br><br>At no time=57%<br>Some of the time=40%<br>Slightly-all the time=3%	**Many had bad conscience all the time and very few some of the time**<br><br>At no time=11%<br>Some of the time=16%<br>Slightly-all the time=73%	**None had bad conscience all the time and very few some of the time**<br><br>At no time=98%<br>Some of the time=2%<br>Slightly-all the time=0%	**Ordinal**<br>0=at no time<br>1=some of the time<br>2=slightly-all the time
**Felt low in spirits/sad (MDI-1)** ¤	Very few had felt sad all the time and few some of the time<br><br>At no time=76%<br>Some of the time=24%<br>Slightly-all the time=1%	Very few had felt sad all the time, many some of the time<br><br>At no time=18%<br>**Some of the time=73%**<br>Slightly-all the time=9%	**None had felt sad all the time and very few some of the time**<br><br>At no time=90%<br>Some of the time=10%<br>Slightly-all the time=0%	Very few had felt sad all the time and half some of the time<br><br>At no time=40%<br>Some of the time=60%<br>Slightly-all the time=1%	Few had felt sad all the time and many some of the time<br><br>At no time=11%<br>Some of the time=61%<br>Slightly-all the time=27%	Very few had felt sad all the time and half some of the time<br><br>At no time=49%<br>Some of the time=47%<br>Slightly-all the time=3%	**Most had felt sad all the time and few some of the time**<br><br>At no time=0%<br>Some of the time=16%<br>Slightly-all the time=84%	**None had felt sad all the time and very few some of the time**<br><br>At no time=98%<br>Some of the time=2%<br>Slightly-all the time=0%	**Ordinal**<br>0=at no time<br>1=some of the time<br>2=slightly-all the time
**Felt lacking in energy and strength (MDI-3)** ¤	Very few felt lacking in energy some of or all the time<br>(IQR=0-1)	**Half felt lacking in energy some of or all the time**<br>(IQR=1-3)	Very few felt lacking in energy some of or all the time<br>(IQR=0-1)	Few felt lacking in energy some of or all the time<br>(IQR=1-1)	**Half felt lacking in energy some of or all the time**<br>(IQR=2-4)	Few felt lacking in energy some of or all the time<br>(IQR=1-1)	**Many felt lacking in energy some of or all the time**<br>(IQR=3-4)	Very few felt lacking in energy some of or all the time<br>(IQR=0-1)	**Ordinal**<br>0=at no time<br>5=all the time
**Felt less self-confident (MDI-4)** ¤	None had felt less self-confident all the time and very few some of the time<br><br>At no time=88%<br>Some of the time=12%<br>Slightly-all the time=0%	Very few had felt less self-confident all the time and half some of the time<br><br>At no time=36%<br>Some of the time=49%<br>Slightly-all the time=15%	**None had felt less self-confident all the time or some of the time**<br><br>At no time=100%<br>Some of the time=0%<br>Slightly-all the time=0%	Very few had felt less self-confident all the time and few some of the time<br><br>At no time=73%<br>Some of the time=23%<br>Slightly-all the time=4%	Few had felt less self-confident all the time or some of the time<br><br>At no time=43%<br>Some of the time=33%<br>Slightly-all the time=24%	Very few had felt less self-confident all the time and few some of the time<br><br>At no time=76%<br>Some of the time=23%<br>Slightly-all the time=1%	**Many had felt less self-confident all the time and few some of the time**<br><br>At no time=5%<br>Some of the time=24%<br>Slightly-all the time=71%	**None had felt less self-confident all the time or some of the time**<br><br>At no time=100%<br>Some of the time=0%<br>Slightly-all the time=0%	**Ordinal**<br>0=at no time<br>1=some of the time<br>2=slightly-all the time
**Had difficulty in concentrating (MDI-7)** ¤	Very few had difficulty in concentrating all the time or some of the time<br><br>At no time=81%<br>Some of the time=17%	Very few had difficulty in concentrating all the time and few some of the time<br><br>At no time=47%<br>Some of the time=38%	**None had difficulty in concentrating all the time and few some of the time**<br><br>At no time=95%<br>Some of the time=5%<br>Slightly-all the	Very few had difficulty in concentrating all the time or some of the time<br><br>At no time=77%<br>Some of the time=19%	Very few had difficulty in concentrating all the time and half some of the time<br><br>At no time=39%<br>Some of the time=42%	Very few had difficulty in concentrating all the time and few some of the time<br><br>At no time=74%<br>Some of the time=23%	**Half had difficulty in concentrating all the time and few some of the time**<br><br>At no time=4%<br>Some of the time=38%	**None had difficulty in concentrating all the time and very few some of the time**<br><br>At no time=94%<br>Some of the time=6%<br>Slightly-all the	**Ordinal**<br>0=at no time<br>1=some of the time<br>2=slightly-all the time

	Slightly-all the time=2%	Slightly-all the time=15%	time=0%	Slightly-all the time=3%	Slightly-all the time=20%	Slightly-all the time=3%	Slightly-all the time=58%	time=0%	
**Have suffered from reduced/increased appetite (MDI-10)** ¤	Very few had reduced/increased appetite all the time or some of the time<br><br>At no time=78%<br>Some of the time=16%<br>Slightly-all the time=5%	Very few had reduced/increased appetite all the time and few some of the time<br><br>At no time=48%<br>Some of the time=34%<br>Slightly-all the time=18%	None had reduced/increased appetite all the time and few some of the time<br><br>At no time=98%<br>Some of the time=2%<br>Slightly-all the time=0%	Very few had reduced/increased appetite all the time or some of the time<br><br>At no time=82%<br>Some of the time=13%<br>Slightly-all the time=6%	**Few had reduced/increased appetite all the time or some of the time**<br><br>At no time=44%<br>Some of the time=31%<br>Slightly-all the time=25%	Very few had reduced/increased appetite all the time or some of the time<br><br>At no time=80%<br>Some of the time=15%<br>Slightly-all the time=5%	**Half had reduced/increased appetite all the time and few some of the time**<br><br>At no time=18%<br>Some of the time=27%<br>Slightly-all the time=55%	Very few had reduced/increased appetite all the time or some of the time<br><br>At no time=85%<br>Some of the time=13%<br>Slightly-all the time=2%	**Ordinal**<br>0=at no time<br>1=some of the time<br>2=slightly-all the time
**More irritable with people than usual (RMDQ-17)** ¤¤	Few more irritable (23%)	Half more irritable (54%)	**Very few more irritable (7%)**	Few more irritable (21%)	Half more irritable (59%)	Few more irritable (37%)	**Most more irritable (91%)**	Few more irritable (25%)	**Dichotomous**<br>0=no<br>1=yes
**Rubbing or holding areas that hurt/are uncomfortable (RMDQ-21)** ¤¤	Few rub/hold areas that hurt (35%)	Half rub/hold areas that hurt (54%)	Few rub/hold areas that hurt (22%)	Half rub/hold areas that hurt (50%)	Half rub/hold areas that hurt (59%)	Half rub/hold areas that hurt (43%)	**Many rub/hold areas that hurt (75%)**	Few rub/hold areas that hurt (33%)	**Dichotomous**<br>0=no<br>1=yes
**Not safe to be physically active (SBT-5)** ¤¤	Very few believe it is not safe to be physically active (14%)	Few believe it is not safe to be physically active (28%)	Very few believe it is not safe to be physically active (5%)	Very few believe it is not safe to be physically active (7%)	Few believe it is not safe to be physically active (24%)	Very few believe it is not safe to be physically active (18%)	Few believe it is not safe to be physically active (33%)	Very few believe it is not safe to be physically active (17%)	Dichotomous<br>0=disagree<br>1=agree
**Terrible back pain, will never get better (SBT-7)** ¤¤	Very few with terrible back pain (1%)	Very few with terrible back pain (11%)	Very few with terrible back pain (1%)	Very few with terrible back pain (5%)	Very few with terrible back pain (15%)	Very few with terrible back pain (1%)	**Few with terrible back pain (26%)**	Very few with terrible back pain (4%)	Dichotomous<br>0=disagree<br>1=agree
**Often express concern (RMDQ-23)** ¤¤	Very few express concern (7%)	Few express concern (26%)	Very few express concern (2%)	Very few express concern (7%)	Few express concern (26%)	Very few express concern (9%)	Half express concern (42%)	Very few express concern (10%)	**Dichotomous**<br>0=no<br>1=yes
**Physical activity might harm back (FABQ-3)** ¤¤¤	Few believe physical activity might harm back<br><br>Disagree=38%<br>Unsure=39%<br>Agree=22%	Few believe physical activity might harm back<br><br>Disagree=31%<br>Unsure=39%<br>Agree=30%	Very few believe physical activity might harm back<br><br>Disagree=59%<br>Unsure=25%<br>Agree=15%	Few believe physical activity might harm back<br><br>Disagree=50%<br>Unsure=28%<br>Agree=21%	Few believe physical activity might harm back<br><br>Disagree=40%<br>Unsure=31%<br>Agree=29%	Very few believe physical activity might harm back<br><br>Disagree=56%<br>Unsure=30%<br>Agree=14%	Very few believe physical activity might harm back<br><br>Disagree=38%<br>Unsure=43%<br>Agree=19%	Few believe physical activity might harm back<br><br>Disagree=48%<br>Unsure=30%<br>Agree=22%	**Ordinal**ǂ<br>0=completely disagree<br>3=unsure<br>6=completely agree
**Should not do physical activity which (might) make worse (FABQ-4)** ¤¤¤	Half believe avoiding physical activity is better<br><br>Disagree=20%<br>Unsure=25%<br>Agree=55%	Many believe avoiding physical activity is better<br><br>Disagree=14%<br>Unsure=25%<br>Agree=61%	Few believe avoiding physical activity is better<br><br>Disagree=37%<br>Unsure=24%<br>Agree=39%	Many believe avoiding physical activity is better<br><br>Disagree=21%<br>Unsure=15%<br>Agree=65%	Many believe avoiding physical activity is better<br><br>Disagree=19%<br>Unsure=18%<br>Agree=63%	Half believe avoiding physical activity is better<br><br>Disagree=22%<br>Unsure=25%<br>Agree=53%	Half believe avoiding physical activity is better<br><br>Disagree=18%<br>Unsure=29%<br>Agree=53%	Half believe avoiding physical activity is better<br><br>Disagree=18%<br>Unsure=29%<br>Agree=53%	**Ordinal**ǂ<br>0=completely disagree<br>3=unsure<br>6=completely agree
**Labelling and clinical description**	**Treatment believers with low degree of depressive mood**<br><br>**Less**<br>• believe that treatment is not essential<br>• with negative recovery belief	**Pain-related concerns, moderate degree of depressive mood**<br><br>**More**<br>• have work concerns (more believe work will harm, they should not do	**The uncomplicated psychological profile**<br><br>**Less**<br>• sleep less well<br>• have work concerns (less believe work will harm or they should not do normal work or	**Sleep well, low degree of depressive mood**<br><br>**Less**<br>• (None) sleep less well<br>• believe they cannot work till pain treated or should	**Treatment believers with sleep issues and moderate degree of depressive mood**<br><br>**Less**<br>• believe that treatment is not essential	**Sleep issues, low degree of pain-related concern**<br><br>**More**<br>• (All) sleep less well<br>• had trouble sleeping<br><br>**Less**	**The complicated psychological profile**<br>**More**<br>• have work concerns (more believe work will harm and that they cannot work till pain treated)<br>• with negative recovery belief	**Pain-related concerns, low degree of depression**<br><br>**More**<br>• believe that treatment is not essential<br>• are not able to decrease pain	

	• feel socially isolated<br>• feel worried	normal work and cannot work till pain treated)<br>• have felt sad some of the time<br>• felt lacking in energy some of the time	cannot work till pain treated)<br>• with negative recovery belief<br>• feel socially isolated<br>• feel worried<br>• have not enjoyed usual things<br>• more irritable<br>• depressive mood:<br>• *have lost interest in daily activities*<br>• *have felt restless*<br>• *had bad conscience some or all the time*<br>• *have felt sad some or all the time*<br>• *(none) had felt less self-confident all the time or some of the time*<br>• *(none) had difficulty in concentrating all the time and few some of the time*<br>• *(none) had suffered from reduced/ increased appetite*	not do normal work with present pain<br>• feel worried	**More**<br>• felt lacking in energy some of the time<br>• had suffered from reduced/increased appetite<br>• had trouble sleeping	• have work concerns (less believe work will harm or they should not do normal work or cannot work till pain treated)	• feel socially isolated<br>• feel worried<br>• rub/holds areas that hurt<br>• have not enjoyed usual things<br>• more irritable<br>• depressive mood:<br>• *have lost interest in daily activities*<br>• *have felt restless*<br>• *had bad conscience some or all the time*<br>• *have felt sad some or all the time*<br>• *felt lacking in energy most of the time*<br>• *had felt less self-confident all the time or some of the time*<br>• *had difficulty in concentrating all the time and few some of the time*<br>• *had trouble sleeping*<br>• *had suffered from reduced/increased appetite*<br>• *had trouble sleeping*	• have work concerns (more believe they should not do normal work and cannot work till pain treated)<br><br>**Less**<br>• with negative recovery belief<br>• feel socially isolated<br>• feel worried<br>• depressive mood:<br>• *have lost interest in daily activities*<br>• *have felt restless*<br>• *had bad conscience some or all the time*<br>• *have felt sad some or all the time*<br>• *had felt less self-confident all the time or some of the time*<br>• *(none) had difficulty in concentrating all the time and few some of the time*	
**Category size, N**	199 (21%)	154 (17%)	132 (14%)	119 (13%)	122 (13%)	99 (11%)	55 (6%)	48 (5%)	
**Posterior probability, median**	0.98 (IQR=0.88-1.00)	0.98 (IQR=0.90-1.00)	0.96 (IQR=0.87-1.00)	0.97 (IQR=0.89-0.99)	0.98 (IQR=0.90-0.99)	0.95 (IQR=0.86-0.99)	0.99 (IQR=0.96-1.00)	0.96 (IQR=0.75-1.00)	
**Posterior probability less than 0.70, N**	20 (10%)	13 (8%)	17 (13%)	10 (8%)	10 (8%)	11 (11%)	4 (7%)	9 (19%)	

Bold text indicates characteristic features which mainly identify the respective domain-specific patient categories
ƚ Trichotomised for the descriptive analysis:0=disagree (0-2), 1=unsure (3), 2=agree (4-6)
IQR = interquartile range; RMDQ = Roland-Morris Disability Questionnaire; FABQ, Fear-Avoidance Beliefs Questionnaire; SBT = STarT Back Tool; MDI = Major Depression Inventory
Symbols and light lines: Variables which have similar scoring pattern across categories representing an overall feature:
*sleep issues
** pain-related concerns
¤ depressive mood
¤¤ catastrophising
¤¤¤ fear avoidance of physical activity

Figure 7. Profile plot of the domain-specific patient categories identified in the psychology domain by the first stage of the two-stage LCA

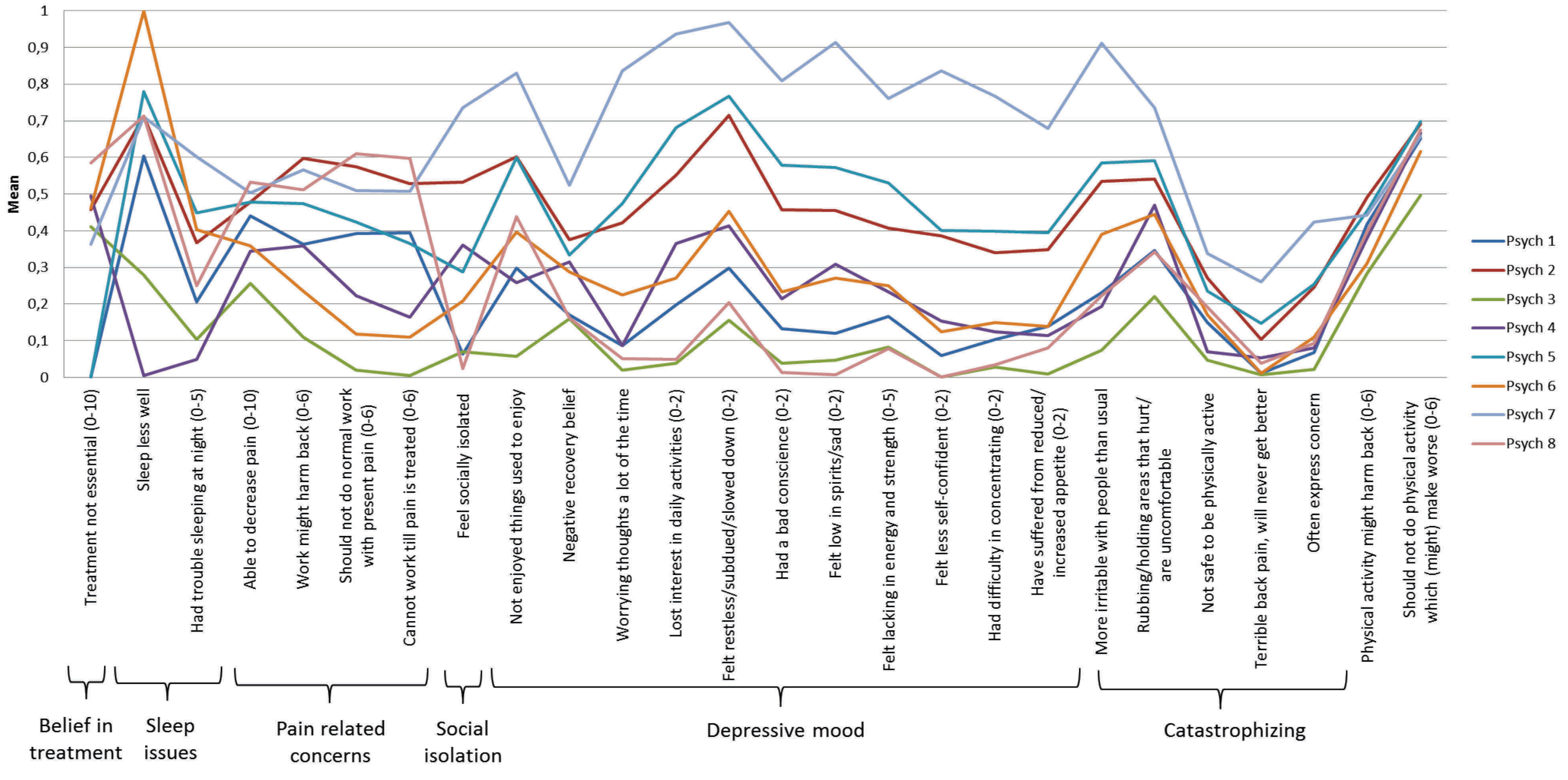

Brief conceptual description of domain-specific patient categories identified in the psychology domain (first stage of two-stage LCA):

Psych 1: Treatment believers with low degree of depressive mood
Psych 2: Pain-related concerns, moderate degree of depressive mood
Psych 3: Uncomplicated psychological profile
Psych 4: Sleep well, low degree of depressive mood
Psych 5: Treatment believers with sleep issues and moderate degree of depressive mood
Psych 6: Sleep issues, low degree of pain-related concerns
Psych 7: The complicated psychological profile
Psych 8: Pain-related concerns, low degree of depressive mood
