# Supplementary material for: Identifying subgroups of patients using latent class analysis: should we use a single-stage or a two-stage approach? A methodological study using a cohort of patients with low back pain
Source: BMC Musculoskelet Disord. 2017 Feb 1;18:57. doi: 10.1186/s12891-017-1411-x (PMC5286735; doi:10.1186/s12891-017-1411-x)
Supplement: Additional file 5: — Statistical characteristics for identified patient subgroup models. One table with statistical characteristics for each LCA approach. (PDF 115 kb) [file 12891_2017_1411_MOESM5_ESM.pdf]

Additional file 5

Table 1. Statistical characteristics for identified patient subgroup models

Approach	Sub-groups	Size of smallest subgroup, N (%)	BIC_{LL}	% change of BIC_{LL}	Frequency of presented BIC	Log-likelihood	Para-meters
Single-stage LCA	1	928 (100)	177996.0		10/10	-88294.2	206
	2	*276 (29.7)*	*172314.1*	*3.19*	*10/10*	*-84992.0*	*341*
	3	163 (17.6)	170843.3	0.85	10/10	-83795.4	476
	4	150 (16.1)	170390.4	0.27	3/10	-83107.7	611
	5	152 (16.4)	170116.5	0.16	3/10	-82509.5	746
	6	123 (13.3)	170193.0	-0.04	3/10	-82086.5	881
	**7**	**77 (8.3)**	**170287.8**	**-0.06**	**1/10**	**-81672.7**	**1016**
	8	73 (7.9)	170539.9	-0.15	1/10	-81337.5	1151
	9	55 (6.0)	170919.8	-0.22	1/10	-81066.3	1286
Two-stage LCA (second stage)	1	928 (100)	20279,3		10/10	-10016,7	36
	2	*(47.8)*	*19892,4*	*1,91*	*10/10*	*-9696,8*	*73*
	3	(17.0)	19947,0	-0,27	8/10	-9597,7	110
	4	(12.7)	20051,9	-0,53	9/10	-9523,7	147
	5	(11.0)	20181,8	-0,65	3/10	-9462,2	184
	6	(6.5)	20344,8	-0,81	2/10	-9417,3	221
	7	(6.4)	20517,9	-0,85	1/10	-9377,5	258
	8	(6.3)	20689,7	-0,84	1/10	-9337,0	295
	**9**	**(5.0)**	**20875,4**	**-0,90**	**1/10**	**-9303,4**	**332**
	10	(4.8)	21055,5	-0,86	1/10	-9267,1	369
	11	(2.1)	21240,8	-0,88	1/10	-9233,3	406

BIC = Bayesian Information Criterion. %-change of BIC: A positive change represents a reduction of BIC
Italics: Selected starting model improving BIC by at least 1%
Bold: Preferred subgroup model
Underline: Best model if based only on lowest BIC
Shown are results of model sizes up to preferred model plus two. Additional results are available on request from the first author
