# Supplementary material for: Identifying subgroups of patients using latent class analysis: should we use a single-stage or a two-stage approach? A methodological study using a cohort of patients with low back pain
Source: BMC Musculoskelet Disord. 2017 Feb 1;18:57. doi: 10.1186/s12891-017-1411-x (PMC5286735; doi:10.1186/s12891-017-1411-x)
Supplement: Additional file 8: — Descriptive presentation of the patient subgroups identified in the single-stage LCA. Presentation of the standardised wording used in the descriptive process and detailed statistical characteristics of each of the patient subgroups. In addition, descriptive presentation of each of the patient subgroups divided into health domain to ease the interpretation. (PDF 442 kb) [file 12891_2017_1411_MOESM8_ESM.pdf]

Additional file 8

This document contains the descriptive presentation of the patient subgroups identified in the single-stage LCA

The descriptive presentation is divided into health domains to ease the interpretation.

Explanatory legend
We described each variable by using:

- standardised descriptive labels for quintiles (see Table 1)
- proportions (%) for nominal or ordinal variables with less than four categories
- interquartile ranges (IQR) for the remaining ordinal items and continuous items

Subgroup size and description of variables are based on the patients assigned to the subgroup for which their posterior probability was the largest

Table 1. Standardised wording used to report the quintiles within the estimates and subgroup profile plots

Range, %	0-19	20-39	40-59	60-79	80-100
'Very few to most'	Very few	Few	Half	Many	Most
'Low – high'	Very low	Low	Moderate	High	Very high
'All or none'	0=none				100=only, all

Table 2. Statistical characteristics of the patient subgroups identified in the single stage LCA

	**SS 1**	**SS 2**	**SS 3**	**SS 4**	**SS 5**	**SS 6**	**SS 7**
**Subgroup size, N**	192 (21%)	154 (17%)	136 (15%)	132 (14%)	130 (14%)	109 (12%)	75 (8%)
**Posterior probability, median**	1.00 (IQR=0.98-1.00)	1.00% (IQR=1.00-1.00)	1.00 (IQR=0.99-1.00)	1.00 (IQR=0.97-1.00)	1.00 (IQR=0.97-1.00)	1.00 (IQR=0.99-1.00)	1.00 (IQR=1.00-1.00)
**Posterior probability less than 0.70, N**	7 (4%)	1 (1%)	3 (2%)	6 (5%)	4 (3%)	3 (3%)	1 (1%)

Table 3a. Descriptive presentation of the seven patient subgroups based on variables from the activity domain, single-stage LCA

Subgroup / Variable	**SS 1**	**SS 2**	**SS 3**	**SS 4**	**SS 5**	**SS 6**	**SS 7**	**Scale**
**Best activity is *not* to walk**	Few do not enjoy walking (32%)	Few do not enjoy walking (28%)	**Half do not enjoy walking (50%)**	Few do not enjoy walking (26%)	Few do not enjoy walking (32%)	**Half do not enjoy walking (45%)**	**Half do not enjoy walking (48%)**	**Dichotomous** 0=no 1=yes
**Only stand for short periods of time (RMDQ-8)***	Very few only stand for short periods (19%)	Very few only stand for short periods (3%)	**Half only stand for short periods (55%)**	Few only stand for short periods (28%)	Very few only stand for short periods (10%)	**Half only stand for short periods (51%)**	**Half only stand for short periods (40%)**	**Dichotomous** 0=no 1=yes
**Only walk short distances (RMDQ-14)***	Few only walk short distances (37%)	**None only walk short distances (0%)**	**Most only walk short distances (84%)**	Few only walk short distances (37%)	**Very few only walk short distances (9%)**	**Many only walk short distances (78%)**	Half only walk short distances (42%)	**Dichotomous** 0=no 1=yes
**Only walked short distances (SBT-3)***	Few have only walked short distances (34%)	**Very few have only walked short distances (1%)**	Half have only walked short distances (57%)	Few have only walked short distances (35%)	Very few have only walked short distances (10%)	**Many have only walked short distances (71%)**	Few have only walked short distances (36%)	**Dichotomous** 0=disagree 1=agree
**Use handrail to get**	Half use handrail to get	**Very few use handrail to**	**Most use handrail to get**	Few use handrail to get	Few use handrail to get	**Most use handrail to get**	Half use handrail to get	**Dichotomous**

**upstairs (RMDQ-5)’**	upstairs<br>(51%)	**get upstairs**<br>**(7%)**	**upstairs**<br>**(83%)**	upstairs<br>(33%)	upstairs<br>(27%)	**upstairs**<br>**(88%)**	upstairs<br>(58%)	0=no<br>1=yes
**Hold on to something to get out of an easy chair (RMDQ-6)***	Many hold on to something to get out of an easy chair<br>(64%)	**Very few hold on to something to get out of an easy chair**<br>**(15%)**	**Most hold on to something to get out of an easy chair**<br>**(94%)**	Half hold on to something to get out of an easy chair<br>(52%)	Few hold on to something to get out of an easy chair<br>(37%)	**Most hold on to something to get out of an easy chair**<br>**(93%)**	Half hold on to something to get out of an easy chair<br>(53%)	**Dichotomous**<br>0=no<br>1=yes
**Difficult to get out of a chair (RMDQ-10)***	Many with difficulties getting out of a chair<br>(74%)	**Very few with difficulties getting out of a chair**<br>**(14%)**	**Most with difficulties getting out of a chair**<br>**(98%)**	Many with difficulties getting out of a chair<br>(60%)	Half with difficulties getting out of a chair<br>(46%)	**Most with difficulties getting out of a chair**<br>**(90%)**	Half with difficulties getting out of a chair<br>(59%)	**Dichotomous**<br>0=no<br>1=yes
**Difficult to turn over in bed (RMDQ-12)***	Many with difficulties turning in bed<br>(79%)	**Few with difficulties turning in bed**<br>**(20%)**	**Most with difficulties turning in bed**<br>**(94%)**	Many with difficulties turning in bed<br>(69%)	Few with difficulties turning in bed<br>(37%)	**Most with difficulties turning in bed**<br>**(85%)**	Many with difficulties turning in bed<br>(60%)	**Dichotomous**<br>0=no<br>1=yes
**Try not to bend or kneel down (RMDQ-9)***	Many try not to bend or kneel down<br>(68%)	**Few try not to bend or kneel down**<br>**(33%)**	**Most try not to bend or kneel down**<br>**(92%)**	Many try not to bend or kneel down<br>(72%)	Many try not to bend or kneel down<br>(60%)	**Most try not to bend or kneel down**<br>**(90%)**	Half try not to bend or kneel down<br>(53%)	**Dichotomous**<br>0=no<br>1=yes
**Dressed more slowly last two weeks (SBT-4)***	Many have dressed more slowly<br>(75%)	**Few have dressed more slowly**<br>**(33%)**	Many have dressed more slowly<br>(75%)	Many have dressed more slowly<br>(68%)	Half have dressed more slowly<br>(48%)	**Most have dressed more slowly**<br>**(91%)**	Many have dressed more slowly<br>(63%)	**Dichotomous**<br>0=disagree<br>1=agree
**Get dressed more slowly (RMDQ-7)***	Many get dressed more slowly<br>(77%)	**Few get dressed more slowly**<br>**(27%)**	**Most get dressed more slowly**<br>**(99%)**	Many get dressed more slowly<br>(69%)	Half get dressed more slowly<br>(52%)	**Most get dressed more slowly**<br>**(91%)**	Many get dressed more slowly<br>(67%)	**Dichotomous**<br>0=no<br>1=yes
**Trouble putting on socks (RMDQ-13)***	**Most have trouble putting on socks**<br>**(80%)**	Few have trouble putting on socks<br>(29%)	**Most have trouble putting on socks**<br>**(95%)**	Half have trouble putting on socks<br>(62%)	Half have trouble putting on socks<br>(56%)	**Most have trouble putting on socks**<br>**(89%)**	Many have trouble putting on socks<br>(65%)	**Dichotomous**<br>0=no<br>1=yes
**Walk more slowly (RMDQ-3)***	Many walk more slowly<br>(78%)	**Very few walk more slowly**<br>**(16%)**	**All walk more slowly**<br>**(100%)**	Many walk more slowly<br>(68%)	Half walk more slowly<br>(40%)	**Most walk more slowly**<br>**(95%)**	**Most walk more slowly**<br>**(82%)**	**Dichotomous**<br>0=no<br>1=yes
**Go upstairs more slowly (RMDQ-18)***	Many go upstairs more slowly<br>(69%)	**Very few go upstairs more slowly**<br>**(14%)**	**Most go upstairs more slowly**<br>**(98%)**	Half go upstairs more slowly<br>(58%)	Half go upstairs more slowly<br>(43%)	**Most go upstairs more slowly**<br>**(96%)**	Many go upstairs more slowly<br>(79%)	**Dichotomous**<br>0=no<br>1=yes
**Avoid heavy jobs around the house (RMDQ-16)***	**Most avoid heavy jobs**<br>**(89%)**	**Few avoid heavy jobs**<br>**(32%)**	**Most avoid heavy jobs**<br>**(95%)**	**Most avoid heavy jobs**<br>**(83%)**	Many avoid heavy jobs<br>(66%)	**Most avoid heavy jobs**<br>**(97%)**	Many avoid heavy jobs<br>(78%)	**Dichotomous**<br>0=no<br>1=yes
**Do less daily work around the house (RMDQ-22)***	Many do less of the daily work<br>(77%)	**Very few do less of the daily work**<br>**(6%)**	**Most do less of the daily work**<br>**(95%)**	Many do less of the daily work<br>(71%)	Half do less of the daily work<br>(52%)	**Most do less of the daily work**<br>**(98%)**	Half do less of the daily work<br>(58%)	**Dichotomous**<br>0=no<br>1=yes
**Not doing usual jobs around the house (RMDQ-4)***	Many are not doing any usual jobs<br>(62%)	**Very few are not doing any usual jobs**<br>**(13%)**	**Most are not doing any usual jobs**<br>**(85%)**	Half are not doing any usual jobs<br>(44%)	Few are not doing any usual jobs<br>(39%)	**Most are not doing any usual jobs**<br>**(83%)**	Half are not doing any usual jobs<br>(51%)	**Dichotomous**<br>0=no<br>1=yes
**Cannot do physical activities which (might) make worse (FABQ-5)***	Few cannot do physical activities which (might) make my pain worse<br><br>Disagree=40%<br>Unsure=23%<br>Agree=37%	**Very few cannot do physical activities which (might) make my pain worse**<br><br>Disagree=63%<br>Unsure=25%<br>**Agree=12%**	**Many cannot do physical activities which (might) make my pain worse**<br><br>Disagree=19%<br>Unsure=21%<br>**Agree=60%**	Half cannot do physical activities which (might) make my pain worse<br><br>Disagree=37%<br>Unsure=23%<br>Agree=40%	Few cannot do physical activities which (might) make my pain worse<br><br>Disagree=47%<br>Unsure=27%<br>Agree=26%	**Many cannot do physical activities which (might) make my pain worse**<br><br>Disagree=14%<br>Unsure=15%<br>**Agree=70%**	Few cannot do physical activities which (might) make my pain worse<br><br>Disagree=38%<br>Unsure=25%<br>Agree=37%	**Ordinal**[ǂ]<br>0=completely disagree<br>3=unsure<br>6=completely agree
**Cannot work with present pain (FABQ-13)**	Very few cannot work with present pain<br><br>Disagree=96%<br>Unsure=3%<br>Agree=1%	None cannot work with present pain<br><br>Disagree=100%<br>Unsure=0%<br>Agree=0%	**Many cannot work with present pain**<br><br>Disagree=19%<br>Unsure=14%<br>**Agree=67%**	Very few cannot work with present pain<br><br>Disagree=71%<br>Unsure=10%<br>Agree=19%	Few cannot work with present pain<br><br>Disagree=57%<br>Unsure=12%<br>Agree=32%	**Most cannot work with present pain**<br><br>Disagree=9%<br>Unsure=9%<br>**Agree=81%**	Very few cannot work with present pain<br><br>Disagree=77%<br>Unsure=3%<br>Agree=19%	**Ordinal**<br>0=disagree<br>1=unsure<br>2=agree
**Best posture to sit**	Few enjoy sitting	Few enjoy sitting	Few enjoy sitting	Very few enjoy sitting	Very few enjoy sitting	Few enjoy sitting	Very few enjoy sitting	**Dichotomous**

	(28%)	(20%)	(25%)	(16%)	(23%)	(22%)	(17%)	0=no<br>1=yes
**Stay in bed most of the time (RMDQ-19)**	Very few stay in bed<br>(1%)	None stay in bed<br>(0%)	Few stay in bed<br>(21%)	Very few stay in bed<br>(5%)	Very few stay in bed<br>(2%)	Very few stay in bed<br>(15%)	Very few stay in bed<br>(3%)	**Dichotomous**<br>0=no<br>1=yes
**Change position frequently (RMDQ-2)**	Most change position frequently<br>(87%)	Many change position frequently<br>(71%)	Most change position frequently<br>(89%)	Most change position frequently<br>(95%)	Most change position frequently<br>(83%)	Most change position frequently<br>(98%)	Most change position frequently<br>(95%)	**Dichotomous**<br>0=no<br>1=yes
**Subgroup description**	**High degree of disability, low degree of walking distance limitations, can work**	**Very low degree of disability, can work**	**High degree of disability**	**Moderate degree of disability, household challenges**	**Moderate degree of disability, low degree of walking distance limitations**	**High degree of disability**	**Moderate degree of disability, walking (speed) limitations**	

Bold text indicates characteristic variables which mainly identify the respective subgroups
ǂ Trichotomised for the descriptive analysis:0=disagree (0-2), 1=unsure (3), 2=agree (4-6)
RMDQ = Roland-Morris Disability Questionnaire; FABQ = Fear-Avoidance Beliefs Questionnaire; SBT = STarT Back Tool; IQR=interquartile range
Symbols and light lines: Variables which have similar scoring pattern across subgroups representing an overall feature:
*Activity limitation

Table 3b. Descriptive presentation of the patient subgroups based on variables from the contextual factors domain, single-stage LCA

Variable \ Subgroup	SS 1	SS 2	SS 3	SS 4	SS 5	SS 6	SS 7	Scale
**Age in years, median**	48<br>(IQR=39-56)	41<br>(IQR=33-49)	43<br>(IQR=35-53)	39<br>(IQR=30-50)	38<br>(IQR=29-51)	44<br>(IQR=35-52)	49<br>(IQR=38-57)	**Continuous**<br>18-66 years of age
**Sex**	Half males<br>(58%)	Half males<br>(58%)	Half males<br>(57%)	Half males<br>(48%)	Many males<br>(76%)	Half males<br>(42%)	Few males<br>(29%)	**Dichotomous**<br>0=female<br>1=male
**Height, median**	176cm (IQR=170-182)	178 cm (IQR=170-183)	176 cm (IQR=169-182)	176 cm (IQR=170-182)	180 cm (IQR=173-185)	172 cm (IQR=167-178)	170 cm (IQR=167-178)	**Continuous**<br>156-201 cm
**BMI, median**	26<br>(IQR=23-30)	25<br>(IQR=23-27)	26<br>(IQR=23-28)	25<br>(IQR=22-28)	26<br>(IQR=23-28)	27<br>(IQR=24-31)	25<br>(IQR=23-27)	**Continuous**<br>Range: 18-59
**Work situation**	Self-employed (14%)<br>Full-time (63%)<br>Part-time (8%)<br>Early retirement/retired (6%)	Self-employed (8%)<br>Full-time (61%)<br>Part-time (16%)<br>Student (8%)	Self-employed (8%)<br>Full-time (66%)<br>Part-time (14%)<br>Student (5%)	Full-time (62%)<br>Part-time (8%)<br>Student (12%)<br>Unemployed (8%)	Self-employed (18%)<br>Full-time (61%)<br>Part-time (9%)<br>Student (5%)	Self-employed (8%)<br>Full-time (51%)<br>Part-time (19%)<br>Unemployed (7%)	Self-employed (19%)<br>Full-time (47%)<br>Part-time (17%)<br>Early retirement/retired (7%)	**Multistate nominal**<br>1=self-employed<br>2=full-time<br>3=part-time<br>4=student<br>5=unemployed<br>6=early retirement/retired<br>7=health-related retirement<br>8=other
**Comorbidity**	Few with other chronic disease (37%):<br>Heart (12%)<br>Asthma (7%)<br>Psychological (3%)<br>Musculoskeletal (12%)<br>Other (12%)	Few with other chronic disease (30%):<br>Heart (5%)<br>Asthma (8%)<br>Psychological (3%)<br>Musculoskeletal (6%)<br>Other (13%)	Few with other chronic disease (24%):<br>Heart (4%)<br>Asthma (5%)<br>Psychological (1%)<br>Musculoskeletal (7%)<br>Other (7%)	**Half with other chronic disease (46%):**<br>Heart (11%)<br>Asthma (8%)<br>Psychological (10%)<br>Musculoskeletal (14%)<br>Other (19%)	Few with other chronic disease (39%):<br>Heart (11%)<br>Asthma (8%)<br>Psychological (1%)<br>Musculoskeletal (15%)<br>Other (8%)	**Half with other chronic disease (53%):**<br>Heart (8%)<br>Asthma (7%)<br>Psychological (7%)<br>Musculoskeletal (21%)<br>Other (17%)	Few with other chronic disease (32%):<br>Heart (9%)<br>Asthma (8%)<br>Musculoskeletal (5%)<br>Other (20%)	**Dichotomous (6 items)**<br>0=no<br>1=yes<br>No other chronic disease<br>Heart disease<br>Asthma<br>Psychological disease<br>Musculoskeletal disease<br>Other chronic disease
**Health insurance (private or work-related)**	Half have health insurance<br>(52%)	Half have health insurance<br>(49%)	Few have health insurance<br>(34%)	Half have health insurance<br>(50%)	Half have health insurance<br>(48%)	Few have health insurance<br>(39%)	Few have health insurance<br>(27%)	**Dichotomous**<br>0=no<br>1=yes<br>*(reversed in the profile plot)*
**Highest educational level**	1=3%<br>2=19%<br>3=15%<br>**4=44%**<br>5=18%	1=5%<br>2=18%<br>3=17%<br>**4=35%**<br>**5=25%**	1=9%<br>**2=31%**<br>3=15%<br>**4=35%**<br>5=10%	1=11%<br>**2=20%**<br>3=12%<br>**4=36%**<br>**5=20%**	1=16%<br>**2=45%**<br>3=14%<br>4=17%<br>5=7%	1=15%<br>**2=31%**<br>3=18%<br>**4=28%**<br>5=8%	1=6%<br>2=20%<br>3=21%<br>**4=44%**<br>5=10%	**Ordinal**<br>1=no qualification<br>2=vocational training<br>3=higher education <3 years<br>4=higher education 3-4 years<br>5=higher education >4 years<br>*(reversed in the profile plot)*
**Self-rated general health, median**	80<br>(IQR=65-86)	80<br>(IQR=73-90)	70<br>(IQR=47-80)	60<br>(IQR=45-72)	75<br>(IQR=65-80)	55<br>(IQR=30-70)	70<br>(IQR=50-80)	**Continuous**<br>Range: 0-100<br>0= lowest<br>100=highest<br>*(reversed in the profile plot)*
**Smoking status**	**Very few smokers**<br><br>Smoker (15%)<br>Ex-smoker (24%)	**Very few smokers**<br><br>Smoker (9%)<br>Ex-smoker (20%)	**Few smokers**<br><br>Smoker (20%)<br>Ex-smoker (25%)	**Few smokers**<br><br>Smoker (23%)<br>Ex-smoker (24%)	**Few smokers**<br><br>Smoker (21%)<br>Ex-smoker (24%)	**Few smokers**<br><br>Smoker (34%)<br>Ex-smoker (21%)	**Very few smokers**<br><br>Smoker (16%)<br>Ex-smoker (21%)	**Ordinal**<br>1=smoker<br>2=ex-smoker<br>3=non-smoker

	Non-smoker (62%)	Non-smoker (70%)	Non-smoker (55%)	Non-smoker (53%)	Non-smoker (55%)	Non-smoker (45%)	Non-smoker (63%)	*(reversed in the profile plot)*
**Claim for compensation**	None have a claim<br><br>Disagree=100%<br>Unsure=0%<br>Agree=0%	Very few have a claim<br><br>Disagree=96%<br>Unsure=1%<br>Agree= 3%	Very few have a claim<br><br>Disagree=98%<br>Unsure=2%<br>Agree=1%	Very few have a claim<br><br>Disagree=91%<br>Unsure=3%<br>Agree=5%	Very few have a claim<br><br>Disagree= 88%<br>Unsure=7%<br>Agree=5%	Very few have a claim<br><br>Disagree=83%<br>Unsure=7%<br>Agree=10%	Very few have a claim<br><br>Disagree=91%<br>Unsure=1%<br>Agree=7%	**Ordinal**<br>0=disagree<br>1=unsure<br>2=agree
**Subgroup description**	**More with higher education ≥3 years**	**More with higher education ≥3 years**	**More with no health insurance**	**Comorbidity, more with higher education ≥3 years**	**Males, more with lower educational level**	**'Unhealthy lifestyle' Higher BMI, lower self-rated general health, smokers, comorbidity**	**Females, more with no health insurance**	

Bold text indicates characteristic features which mainly identify the respective subgroups
IQR=inter quartile range
Work situation: Only mentioned if percentage >5%

Table 3c. Descriptive presentation of the patient subgroups based on variables from the pain domain, single-stage LCA

Variable \ Subgroup	SS 1	SS 2	SS 3	SS 4	SS 5	SS 6	SS 7	Scale
**Previous LBP episodes**	Half have had more than three episodes<br><br>None=16%<br>1-3=32%<br>>3=52%	Half have had more than three episodes<br><br>None=20%<br>1-3=35%<br>>3=45%	Half have had more than three episodes<br><br>None=14%<br>1-3=45%<br>>3=40%	Half have had more than three episodes<br><br>None=15%<br>1-3=35%<br>>3=50%	Half have had more than three episodes<br><br>None=20%<br>1-3=37%<br>>3=43%	Half have had more than three episodes<br><br>None=17%<br>1-3=27%<br>>3=56%	Half have had more than three episodes<br><br>None=8%<br>1-3=32%<br>>3=59%	**Ordinal**<br>0=no previous episodes<br>1=1-3 previous episodes<br>2=more than 3 previous episodes
**Duration of LBP***	**0-2 weeks duration of LBP**<br><br>**0-2 weeks=73%**<br>2-4 weeks=10%<br>1-3 months=9%<br>>3 months=7%	**0-4 weeks duration of LBP**<br>**(Any duration)**<br><br>**0-2 weeks=41%**<br>**2-4 weeks=21%**<br>1-3 months=16%<br>>3 months=22%	**0-2 weeks duration of LBP**<br><br>**0-2 weeks=97%**<br>2-4 weeks=2%<br>1-3 months=1%<br>>3 months=1%	**0-4 weeks duration of LBP**<br>**(Any duration)**<br><br>**0-2 weeks=41%**<br>**2-4 weeks=21%**<br>1-3 months=13%<br>>3 months=25%	**0-4 weeks duration of LBP**<br><br>**0-2 weeks=56%**<br>**2-4 weeks=16%**<br>1-3 months=10%<br>>3 months=18%	**0-2 weeks duration of LBP**<br><br>**0-2 weeks=71%**<br>2-4 weeks=12%<br>1-3 months=8%<br>>3 months=9%	**0-4 weeks duration of LBP**<br><br>**0-2 weeks=56%**<br>**2-4 weeks=11%**<br>1-3 months=19%<br>>3 months=14%	**Ordinal**<br>1=0-2 weeks<br>2=2-4 weeks<br>3=1-3 months<br>4=>3 months
**More than 30 days of LBP last year***	Few with more than 30 days of back pain last year<br>(20%)	Few with more than 30 days of back pain last year<br>(32%)	Very few with more than 30 days of back pain last year<br>(6%)	**Half with more than 30 days of back pain last year**<br>**(41%)**	Very few with more than 30 days of back pain last year<br>(27%)	Few with more than 30 days of back pain last year<br>(24%)	Few with more than 30 days of back pain last year<br>(37%)	**Dichotomous**<br>0=≤ 30 days<br>1=>30 days
**LBP intensity****	High intensity LBP<br>(IQR=5-8)	Moderate intensity LBP<br>(IQR=3-6)	**Very high intensity LBP**<br>**(IQR=7-9)**	High intensity LBP<br>(IQR=6-8)	High intensity LBP<br>(IQR=5-8)	**Very high intensity LBP**<br>**(IQR=7-9)**	High intensity LBP<br>(IQR=6-8)	**Continuous**<br>0=no pain<br>10=worst imaginable pain
**Back/leg painful almost all the time (RMDQ-11)****	Many with pain almost all the time<br>(63%)	Few with pain almost all the time<br>(27%)	**Most with pain almost all the time**<br>**(87%)**	Many with pain almost all the time<br>(75%)	Half with pain almost all the time<br>(56%)	**Most with pain almost all the time**<br>**(92%)**	**Most with pain almost all the time**<br>**(86%)**	**Dichotomous**<br>0=no<br>1=yes
**Very-extremely bothersome back pain last 2 weeks (SBT-9)****	Many with high degree of bothersome pain<br>(61%)	Very few with high degree of bothersome pain<br>(19%)	**Most with high degree of bothersome pain**<br>**(93%)**	Many with high degree of bothersome pain<br>(79%)	Many with high degree of bothersome pain<br>(68%)	**Most with high degree of bothersome pain**<br>**(97%)**	**Most with high degree of bothersome pain**<br>**(80%)**	**Dichotomous**<br>0=no-moderately bothersome pain<br>1=very-extremely bothersome pain
**LBP not dominating**	Very few LBP not dominating<br>(2%)	Very few LBP not dominating<br>(5%)	Very few LBP not dominating<br>(1%)	Very few LBP not dominating<br>(3%)	Very few LBP not dominating<br>(5%)	Very few LBP not dominating<br>(8%)	**Half LBP not dominating**<br>**(57%)**	**Dichotomous**<br>0=no<br>1=yes
**Leg pain intensity*****	Half with no leg pain<br>(50%)<br>Few with low intensity leg pain (35%)<br>Very few with moderate-high intensity leg pain<br>(15%)	Many with no leg pain<br>(63%)<br>Few with low intensity leg pain<br>(25%)<br>Very few with moderate-high intensity leg pain<br>(12%)	Half with no leg pain<br>(56%)<br>Few with low intensity leg pain (31%)<br>Very few with moderate-high intensity leg pain<br>(13%)	Few with no leg pain<br>(34%)<br>Half with low intensity leg pain<br>(48%)<br>Very few with moderate-high intensity leg pain<br>(18%)	Half with no leg pain<br>(40%)<br>Half with low intensity leg pain (40%)<br>Few with moderate-high intensity leg pain (20%)	Few with no leg pain<br>(23%)<br>Few with low intensity leg pain<br>(32%)<br>**Half with moderate-high intensity leg pain (45%)**	None with no leg pain<br>(0%)<br>Very few with low intensity leg pain<br>(16%)<br>**Most with moderate-high intensity leg pain**<br>**(84%)**	**Ordinal**<br>0=no pain<br>1=mild pain<br>2=moderate-worst imaginable pain
**Pain has spread down leg(s) at some time last 2 weeks (SBT-1)*****	Few with spread of pain to leg(s)<br>(30%)	Few with spread of pain to leg(s)<br>(25%)	Few with spread of pain to leg(s)<br>(27%)	Half with spread of pain to leg(s)<br>(42%)	Half with spread of pain to leg(s)<br>(44%)	**Many with spread of pain to leg(s)**<br>**(69%)**	**Most with spread of pain to leg(s)**<br>**(97%)**	**Dichotomous**<br>0=no<br>1=yes
**Pain distribution*****	Many with LBP only<br><br>Only LBP (79%)<br>LBP and pain in one leg<br>(18%)<br>LBP and pain in both legs<br>(2%)	Most with LBP only<br><br>Only LBP (81%)<br>LBP and pain in one leg<br>(16%)<br>LBP and pain in both legs<br>(1%)	Most with LBP only<br><br>Only LBP (83%)<br>LBP and pain in one leg<br>(16%)<br>LBP and pain in both legs<br>2%)	Many with LBP only<br><br>Only LBP (74%)<br>LBP and pain in one leg<br>(19%)<br>LBP and pain in both legs<br>(6%)	Many with LBP only<br><br>Only LBP (74%)<br>LBP and pain in one leg<br>(16%)<br>LBP and pain in both legs<br>(9%)	Half with LBP only and half with LBP and unilateral leg pain<br><br>Only LBP (50%)<br>LBP and pain in one leg<br>(40%)	**Many with LBP and unilateral leg pain or leg pain only**<br><br>Only LBP (4%)<br>**LBP and pain in one leg**<br>**(77%)**	**Multistate nominal**<br>0=only low back pain<br>1=low back pain and pain in one leg<br>2=low back pain and pain in both legs<br>3=only leg pain

	Only leg pain (1%)	Only leg pain (3%)	Only leg pain (0%)	Only leg pain (1%)	Only leg pain (1%)	LBP and pain in both legs (10%)<br>Only leg pain (0%)	LBP and pain in both legs (3%)<br>**Only leg pain (16%)**	
**Pain in shoulder/neck last 2 weeks (SBT-2)**	Few with shoulder/neck pain (32%)	Half with shoulder/neck pain (53%)	Few with shoulder/neck pain (29%)	**Many with shoulder/neck pain (69%)**	Half with shoulder/neck pain (54%)	Half with shoulder/neck pain (54%)	Half with shoulder/neck pain (53%)	**Dichotomous**<br>0=no<br>1=yes
**No paraspinal pain onset**	Few with no paraspinal onset (37%)	Few with no paraspinal onset (39%)	Few with no paraspinal onset (30%)	Few with no paraspinal onset (33%)	Few with no paraspinal onset (32%)	Half with no paraspinal onset (46%)	**Many with no paraspinal onset (66%)**	**Dichotomous**<br>0=no<br>1=yes
**Pain caused by physical activity (FABQ-1)**	Few had pain caused by physical activity<br>Disagree=36%<br>Unsure=32%<br>Agree=32%	Half had pain caused by physical activity<br>Disagree=37%<br>Unsure=23%<br>Agree=41%	Half had pain caused by physical activity<br>Disagree=32%<br>Unsure=19%<br>Agree=48%	Few had pain caused by physical activity<br>Disagree=32%<br>Unsure=28%<br>Agree=39%	**Many had pain caused by physical activity**<br>Disagree=13%<br>Unsure=24%<br>**Agree=63%**	Half had pain caused by physical activity<br>Disagree=15%<br>Unsure=34%<br>Agree=51%	Few had pain caused by physical activity<br>Disagree=25%<br>Unsure=37%<br>Agree=38%	**Ordinal**[ƚ]<br>0=completely disagree<br>3=unsure<br>6=completely agree
**Physical activity makes worse (FABQ-2)**	Half get worse by physical activity<br>Disagree=44%<br>Unsure=150%<br>Agree=41%	Few get worse by physical activity<br>Disagree=46%<br>Unsure=15%<br>Agree=39%	**Many get worse by physical activity**<br>Disagree=20%<br>Unsure=20%<br>**Agree=60%**	Half get worse by physical activity<br>Disagree=35%<br>Unsure=25%<br>Agree=40%	Half get worse by physical activity<br>Disagree=23%<br>Unsure=18%<br>Agree=58%	**Many get worse by physical activity**<br>Disagree=9%<br>Unsure=17%<br>**Agree=74%**	Half get worse by physical activity<br>Disagree=36%<br>Unsure=15%<br>Agree=49%	**Ordinal**[ƚ]<br>0=completely disagree<br>3=unsure<br>6=completely agree
**Subgroup description**	**Recent LBP with high degree of back pain severity**	**Intermittent LBP with moderate degree of back pain severity**	**Recent LBP with very high degree of back pain severity, worsened by physical activity**	**Persistent LBP with high degree of back pain severity and shoulder/neck pain**	**Recent LBP with high degree of back pain severity and low degree of leg pain severity**	**Recent LBP with very high back pain severity and moderate degree of leg pain severity, worsened by physical activity**	**Dominating leg pain, high degree of leg pain severity, no paraspinal pain onset**	

Bold text indicates characteristic variables which mainly identify the respective subgroups

[ƚ] Trichotomised for the descriptive analysis:0=disagree (0-2), 1=unsure (3), 2=agree (4-6)

LBP = low back pain; IQR=inter quartile range; RMDQ = Roland-Morris Disability Questionnaire; FABQ = Fear-Avoidance Beliefs Questionnaire; SBT = STarT Back Tool

Symbols and light lines: Variables which have similar scoring pattern across subgroups representing an overall feature:

*LBP duration

**Back pain severity

***Leg pain severity

Table 3d. Descriptive presentation of the patient subgroups based on variables from the participation domain, single-stage LCA

Variable / Subgroup	SS 1	SS 2	SS 3	SS 4	SS 5	SS 6	SS 7	Scale
**Days with sick leave last month***	Most with no sick leave (91%)<br>Very few with 2-5 days of sick leave (9%)<br>None with 6-31 days of sick leave (0%)	Most with no sick leave (96%)<br>Very few with 2-5 days of sick leave (4%)<br>None with 6-31 days of sick leave (0%)	Half with no sick leave (59%)<br>**Half with 2-5 days of sick leave (40%)**<br>Very few with 6-31 days of sick leave (2%)	Many with no sick leave (76%)<br>Few with 2-5 days of sick leave (22%)<br>Very few with 6-31 days of sick leave (2%)	Most with no sick leave (82%)<br>Few with 2-5 days of sick leave (14%)<br>Very few with 6-31 days of sick leave (3%)	Half with no sick leave (46%)<br>**Half with 2-5 days of sick leave (42%)**<br>**Very few with 6-31 days of sick leave (11%)**	Most with no sick leave (80%)<br>Very few with 2-5 days of sick leave (17%)<br>Few with 6-31 days of sick leave (3%)	**Ordinal**<br>0=0 days<br>1=1-5 days (workweek)<br>2=6-31 days
**Decreased sexual activity (RMDQ-20)***	**Few with decreased sexual activity (36%)**	Very few with decreased sexual activity (2%)	**Many with decreased sexual activity (61%)**	Few with decreased sexual activity (39%)	Very few with decreased sexual activity (13%)	**Many with decreased sexual activity (73%)**	Few with decreased sexual activity (38%)	**Dichotomous**<br>0=no<br>1=yes
**Stay home most of the time (RMDQ-1)***	Very few stay home most of the time (6%)	None stay home most of the time (0%)	**Many stay home most of the time (66%)**	Very few stay home most of the time (12%)	Very few stay home most of the time (6%)	**Many stay home most of the time (61%)**	Very few stay home most of the time (12%)	**Dichotomous**<br>0=no<br>1=yes
**Work is too heavy (FABQ-9)****	None believe work is too heavy<br><br>Disagree=99%<br>Unsure=1%<br>Agree=0%	Very few believe work is too heavy<br><br>Disagree=96%<br>Unsure=2%<br>Agree=1%	Very few believe work is too heavy<br><br>Disagree=85%<br>Unsure=5%<br>Agree=10%	Very few believe work is too heavy<br><br>Disagree=80%<br>Unsure=7%<br>Agree=13%	Few believe work is too heavy<br><br>Disagree=44%<br>Unsure=18%<br>Agree=38%	**Half believe work is too heavy**<br><br>Disagree=33%<br>Unsure=20%<br>**Agree=46%**	Very few believe work is too heavy<br><br>Disagree=76%<br>Unsure=15%<br>Agree=10%	**Ordinal**<br>0=disagree<br>1=unsure<br>2=agree
**Work makes/would make pain worse (FABQ-10)****	Very few believe work makes pain worse<br><br>Disagree=88%<br>Unsure=7%<br>Agree=5%	Very few believe work makes pain worse<br><br>Disagree=79%<br>Unsure=12%<br>Agree=9%	Few believe work makes pain worse<br><br>Disagree=48%<br>Unsure=24%<br>Agree=28%	Few believe work makes pain worse<br><br>Disagree=54%<br>Unsure=21%<br>Agree=25%	**Many believe work makes pain worse**<br><br>Disagree=15%<br>Unsure=24%<br>**Agree=61%**	**Many believe work makes pain worse**<br><br>Disagree=16%<br>Unsure=24%<br>**Agree=60%**	Few believe work makes pain worse<br><br>Disagree=56%<br>Unsure=19%<br>Agree=24%	**Ordinal**<br>0=disagree<br>1=unsure<br>2=agree
**Work-aggravated pain (FABQ-7)****	Very few believe work-aggravated pain<br><br>Disagree=79%<br>Unsure=13%<br>Agree=7%	Very few believe work-aggravated pain<br><br>Disagree=77%<br>Unsure=12%<br>Agree=11%	Few believe work-aggravated pain<br><br>Disagree=52%<br>Unsure=23%<br>Agree=26%	Few believe work-aggravated pain<br><br>Disagree=34%<br>Unsure=33%<br>Agree=34%	**Half believe work-aggravated pain**<br><br>Disagree=17%<br>Unsure=26%<br>**Agree=57%**	**Many believe work-aggravated pain**<br><br>Disagree=21%<br>Unsure=19%<br>**Agree=60%**	Few believe work-aggravated pain<br><br>Disagree=43%<br>Unsure=21%<br>Agree=36%	**Ordinal**<br>0=disagree<br>1=unsure<br>2=agree
**Pain caused by work/accident at work (FABQ-6)****	Very few believe pain caused by work/accident at work<br><br>Disagree=88%<br>Unsure=6%<br>Agree=6%	Very few believe pain caused by work/accident at work<br><br>Disagree=80%<br>Unsure=7%<br>Agree=13%	Few believe pain caused by work/accident at work<br><br>Disagree=64%<br>Unsure=16%<br>Agree=20%	Few believe pain caused by work/accident at work<br><br>Disagree=57%<br>Unsure=19%<br>Agree=24%	Half believe pain caused by work/accident at work<br><br>Disagree=34%<br>Unsure=20%<br>Agree=46%	Half believe pain caused by work/accident at work<br><br>Disagree=38%<br>Unsure=15%<br>Agree=47%	Few believe pain caused by work/accident at work<br><br>Disagree=56%<br>Unsure=17%<br>Agree=27%	**Ordinal**<br>0=disagree<br>1=unsure<br>2=agree
**Physical workload**	Sitting and walking at work<br><br>Few only sit at work (31%)<br>Half sit and walk at work (48%)<br>Very few have light physical load (18%)<br>Very few have heavy physical load (3%)	Sitting and walking at work<br><br>Few only sit at work (33%)<br>Half sit and walk at work (42%)<br>Very few have light physical load (15%)<br>Very few have heavy physical load (10%)	Any physical workload<br><br>Few only sit at work (20%)<br>Few sit and walk at work (34%)<br>Few have light physical load (24%)<br>Few have heavy physical load (21%)	Sitting and walking at work<br><br>Few only sit at work (32%)<br>Few sit and walk at work (38%)<br>Very few have light physical load (18%)<br>Very few have heavy physical load (11%)	**Light-heavy physical workload**<br><br>Very few only sit at work (7%)<br>Very few sit and walk at work (12%)<br>**Few have light physical load (26%)**<br>**Half have heavy physical load (55%)**	**Light-heavy physical workload**<br><br>Very few only sit at work (7%)<br>Very few sit and walk at work (18%)<br>**Few have light physical load (30%)**<br>**Half have heavy physical load (45%)**	Sitting and walking at work<br><br>Few only sit at work (30%)<br>Half sit and walk at work (42%)<br>Very few have light physical load (12%)<br>Very few have heavy physical load (15%)	**Ordinal**<br>1=sitting<br>2=sitting and walking<br>3=light physical load<br>4=heavy physical load

Subgroup description	Moderate degree of social participation limitations	Low degree of participation limitations	High degree of social participation limitations, moderate degree of work issues	Moderate degree of work participation limitations	High degree of work issues and physical workload	High degree of participation limitations and high degree of physical workload	Moderate degree of participation limitations	

Bold text indicates characteristic variables which mainly identify the respective subgroups
IQR= interquartile range; FABQ-work = Fear-Avoidance Beliefs Questionnaire, work subscale
Symbols and light lines: Variables which have similar scoring pattern across subgroups representing an overall feature:
*Social participation limitations
** Work issues

Table 3e. Descriptive presentation of the patient subgroups based on variables from the physical impairment domain, single-stage LCA

Subgroup / Variable	SS 1	SS 2	SS 3	SS 4	SS 5	SS 6	SS 7	Scale
**Pain on flexion (ROM)***	Few with no pain (33%)<br>**Many with back pain (67%)**<br>Very few with leg pain w/wo back pain (1%)	**Half with no pain (55%)**<br>Half with back pain (44%)<br>Very few with leg pain w/wo back pain (1%)	Very few with no pain (14%)<br>**Most with back pain (86%)**<br>None with leg pain w/wo back pain (0%)	Few with no pain (39%)<br>**Many with back pain (60%)**<br>Very few with leg pain w/wo back pain (1%)	Few with no pain (30%)<br>**Many with back pain (67%)**<br>Very few with leg pain w/wo back pain (3%)	Few with no pain (20%)<br>**Many with back pain (61%)**<br>**Few with leg pain w/wo back pain (20%)**	Few with no pain (29%)<br>Very few with back pain (16%)<br>**Half with leg pain w/wo back pain (54%)**	**Multistate nominal**<br>0=no pain<br>1=back pain<br>2=leg pain w/wo back pain
**Pain on extension (ROM)***	Few with no pain (29%)<br>**Many with back pain (71%)**<br>None with leg pain w/wo back pain (0%)	**Half with no pain (50%)**<br>Half with back pain (48%)<br>Very few with leg pain w/wo back pain (2%)	Few with no pain (27%)<br>**Many with back pain (73%)**<br>Very few with leg pain w/wo back pain (1%)	Few with no pain (37%)<br>**Many with back pain (63%)**<br>None with leg pain w/wo back pain (0%)	Few with no pain (34%)<br>**Many with back pain (66%)**<br>Very few with leg pain w/wo back pain (1%)	Few with no pain (24%)<br>**Many with back pain (64%)**<br>**Very few with leg pain w/wo back pain (12%)**	Half with no pain (44%)<br>Few with back pain (32%)<br>**Few with leg pain w/wo back pain (24%)**	**Multistate nominal**<br>0=no pain<br>1=back pain<br>2=leg pain w/wo back pain
**Pain on right side glide (ROM)***	Half with no pain (52%)<br>**Half with back pain (48%)**<br>None with leg pain w/wo back pain (0%)	**Many with no pain (68%)**<br>Few with back pain (31%)<br>Very few with leg pain w/wo back pain (1%)	Half with no pain (47%)<br>**Half with back pain (53%)**<br>None with leg pain w/wo back pain (1%)	Half with no pain (52%)<br>**Half with back pain (48%)**<br>None with leg pain w/wo back pain (0%)	Half with no pain (51%)<br>**Half with back pain (49%)**<br>None with leg pain w/wo back pain (0%)	Half with no pain (46%)<br>Half with back pain (49%)<br>Very few with leg pain w/wo back pain (5%)	Half with no pain (57%)<br>Very few with back pain (18%)<br>**Few with leg pain w/wo back pain (25%)**	**Multistate nominal**<br>0=no pain<br>1=back pain<br>2=leg pain w/wo back pain
**Pain on left side glide (ROM)***	Half with no pain (58%)<br>**Half with back pain (42%)**<br>None with leg pain w/wo back pain (0%)	**Many with no pain (64%)**<br>Few with back pain (36%)<br>None with leg pain w/wo back pain (0%)	Half with no pain (46%)<br>**Half with back pain (54%)**<br>None with leg pain w/wo back pain (0%)	Half with no pain (58%)<br>**Half with back pain (41%)**<br>Very few with leg pain w/wo back pain (2%)	**Many with no pain (64%)**<br>**Few with back pain (36%)**<br>None with leg pain w/wo back pain (0%)	Few with no pain (39%)<br>Half with back pain (51%)<br>Very few with leg pain w/wo back pain (10%)	Half with no pain (44%)<br>Few with back pain (28%)<br>**Few with leg pain w/wo back pain (28%)**	**Multistate nominal**<br>0=no pain<br>1=back pain<br>2=leg pain w/wo back pain
**Pain on right rotation (ROM)***	Many with no pain (75%)<br>Few with back pain (25%)<br>None with leg pain w/wo back pain (0%)	**Most with no pain (87%)**<br>Very few with back pain (13%)<br>None with leg pain w/wo back pain (0%)	Many with no pain (64%)<br>**Few with back pain (36%)**<br>None with leg pain w/wo back pain (0%)	Many with no pain (76%)<br>Few with back pain (24%)<br>None with leg pain w/wo back pain (0%)	Many with no pain (75%)<br>Few with back pain (24%)<br>Very few with leg pain w/wo back pain (1%)	Many with no pain (69%)<br>Few with back pain (30%)<br>Very few with leg pain w/wo back pain (1%)	Many with no pain (76%)<br>Very few with back pain (9%)<br>**Very few with leg pain w/wo back pain (15%)**	**Multistate nominal**<br>0=no pain<br>1=back pain<br>2=leg pain w/wo back pain
**Pain on left rotation (ROM)***	Many with no pain (70%)<br>Few with back pain (30%)<br>None with leg pain w/wo back pain (0%)	**Most with no pain (90%)**<br>Very few with back pain (10%)<br>None with leg pain w/wo back pain (0%)	Many with no pain (64%)<br>**Few with back pain (36%)**<br>None with leg pain w/wo back pain (0%)	Many with no pain (71%)<br>Few with back pain (29%)<br>None with leg pain w/wo back pain (0%)	Most with no pain (81%)<br>Very few with back pain (19%)<br>None with leg pain w/wo back pain (0%)	Many with no pain (63%)<br>Few with back pain (34%)<br>Very few with leg pain w/wo back pain (3%)	Many with no pain (76%)<br>Very few with back pain (6%)<br>**Very few with leg pain w/wo back pain (18%)**	**Multistate nominal**<br>0=no pain<br>1=back pain<br>2=leg pain w/wo back pain
**Diagnosis based on 'McKenzie testing'**	Few with reducible disc (29%)<br>Few with partly reducible disc (22%)	Few with reducible disc (26%)<br>Very few with partly reducible disc (16%)	Few with reducible disc (23%)<br>Few with partly reducible disc (33%)	Very few with reducible disc (18%)<br>Few with partly reducible disc (26%)	Very few with reducible disc (18%)<br>Few with partly reducible disc (23%)	Few with reducible disc (22%)<br>Few with partly reducible disc (28%)	Few with reducible disc (23%)<br>Few with partly reducible disc (21%)	**Dichotomous (4 items)**<br>0=no<br>1=yes

	Very few with non-reducible disc (8%)<br>Very few with dysfunction (10%)	None with non-reducible disc (0%)<br>Very few with dysfunction (16%)	Very few with non-reducible disc (4%)<br>Very few with dysfunction (5%)	Very few with non-reducible disc (5%)<br>Very few with dysfunction (10%)	Very few with non-reducible disc (2%)<br>Very few with dysfunction (17%)	Very few with non-reducible disc (11%)<br>Very few with dysfunction (6%)	Very few with non-reducible disc (15%)<br>Very few with dysfunction (3%)	Reducible disc<br>Partly reducible disc<br>Non-reducible disc<br>Dysfunction
**Positive neurological examination****	Very few with indication of herniated disc (3% right, 1% left)<br>Very few with affected muscular strength (2%), sensibility (5%) or reflexes (7%)	Very few with indication of herniated disc (3% right, 3% left)<br>Very few with affected muscular strength (1%), sensibility (3%) or reflexes (5%)	Very few with indication of herniated disc (5% right, 6% left)<br>Very few with affected muscular strength (2%), sensibility (1%) or reflexes (7%)	Very few with indication of herniated disc (4% right, 2% left)<br>Very few with affected muscular strength (1%), sensibility (0%) or reflexes (3%)	Very few with indication of herniated disc (4% right, 3% left)<br>Very few with affected muscular strength (0%), sensibility (4%) or reflexes (2%)	Very few with indication of herniated disc (18% right, 17% left)<br>Very few with affected muscular strength (8%), sensibility (7%) or reflexes (11%)	**Few with indication of herniated disc (13% right, 22% left)**<br>**Few-half with affected muscular strength (20%), sensibility (31%) or reflexes (29%)**	**Dichotomous (5 items)**<br>0=no<br>1=yes<br>Indication of herniated disc, right side<br>Indication of herniated disc, left side<br>Affected muscular strength<br>Affected sensibility<br>Affected deep tendon reflexes
**Positive sacroiliac joint tests*****	Few with positive tests:<br>Separation (8%)<br>Thigh thrust (20%)<br>Gaenslen's (19%)<br>Compression (15%)<br>Sacral thrust (37%)	Few with positive tests:<br>Separation (11%)<br>Thigh thrust (25%)<br>Gaenslen's (18%)<br>Compression (12%)<br>Sacral thrust (28%)	Few with positive tests:<br>Separation (14%)<br>Thigh thrust (16%)<br>Gaenslen's (20%)<br>Compression (20%)<br>Sacral thrust (35%)	Few with positive tests:<br>Separation (9%)<br>Thigh thrust (29%)<br>Gaenslen's (21%)<br>Compression (14%)<br>Sacral thrust (33%)	Few with positive tests:<br>Separation (11%)<br>Thigh thrust (23%)<br>Gaenslen's (18%)<br>Compression (13%)<br>Sacral thrust (25%)	**Half with positive tests:**<br>**Separation (14%)**<br>**Thigh thrust (46%)**<br>**Gaenslen's (39%)**<br>**Compression (30%)**<br>**Sacral thrust (42%)**	Few with positive tests:<br>Separation (10%)<br>Thigh thrust (28%)<br>Gaenslen's (16%)<br>Compression (16%)<br>Sacral thrust (35%)	**Dichotomous (5 items)**<br>0=negative<br>1=positive<br>Separation test<br>Thigh thrust<br>Gaenslen's test,<br>Compression test,<br>Sacral thrust
**Pain on combined extension/rotation**	Half with pain on extension/rotation (43%)	Half with pain on extension/rotation (54%)	Few with pain on extension/rotation (39%)	Half with pain on extension/rotation (45%)	Half with pain on extension/rotation (42%)	Few with pain on extension/rotation (35%)	**Many with pain on extension/rotation (68%)**	**Dichotomous**<br>0=no<br>1=yes
**Trigger points**	Few with replication of pain by trigger point (28%)	Few with replication of pain by trigger point (22%)	Very few with replication of pain by trigger point (17%)	Few with replication of pain by trigger point (34%)	Very few with replication of pain by trigger point (16%)	Few with replication of pain by trigger point (21%)	**Half with replication of pain by trigger point (49%)**	**Dichotomous**<br>0=no<br>1=yes
**Pain on muscle palpation**	Many with replication of pain on muscle palpation (61%)	Half with replication of pain on muscle palpation (58%)	Half with replication of pain on muscle palpation (47%)	Many with replication of pain on muscle palpation (60%)	Half with replication of pain on muscle palpation (57%)	Half with replication of pain on muscle palpation (51%)	**Many with replication of pain on muscle palpation (77%)**	**Dichotomous**<br>0=no<br>1=yes
**Painful muscle groups**	**Half with painful back muscles (40%)**<br>Few with painful buttock and leg muscles (37%)<br>Few with both painful back and buttock/leg muscles (23%)	Few with painful back muscles (34%)<br>**Half with painful buttock and leg muscles (45%)**<br>Few with both painful back and buttock/leg muscles (21%)	Few with painful back muscles (37%)<br>Few with painful buttock and leg muscles (29%)<br>Few with both painful back and buttock/leg muscles (33%)	Few with painful back muscles (32%)<br>Few with painful buttock and leg muscles (28%)<br>**Half with both painful back and buttock/leg muscles (40%)**	**Half with painful back muscles (48%)**<br>Few with painful buttock and leg muscles (32%)<br>Few with both painful back and buttock/leg muscles (20%)	Few with painful back muscles (31%)<br>**Half with painful buttock and leg muscles (51%)**<br>Very few with both painful back and buttock/leg muscles (18%)	Very few with painful back muscles (13%)<br>**Half with painful buttock and leg muscles (58%)**<br>Few with both painful back and buttock/leg muscles (29%)	**Multistate nominal**<br>0=back muscles<br>1=buttock and leg muscles<br>2=back and buttock/leg muscles
**Acute lateral shift**	Very few with acute lateral shift (13%)	Very few with acute lateral shift (2%)	Few with acute lateral shift (27%)	Very few with acute lateral shift (3%)	Very few with acute lateral shift (4%)	Very few with acute lateral shift (16%)	Very few with acute lateral shift (6%)	**Dichotomous**<br>0=no<br>1=yes
**Acute flexion deformity**	Very few with acute flexion deformity (3%)	None with acute flexion deformity (0%)	Very few with acute flexion deformity (12%)	Very few with acute flexion deformity (5%)	Very few with acute flexion deformity (3%)	Very few with acute flexion deformity (9%)	Very few with acute flexion deformity (3%)	**Dichotomous**<br>0=no<br>1=yes
**Subgroup description**	**LBP on flexion, extension and side glide and painful back muscles**	**Low degree of pain on ROM, painful buttock and leg muscles**	**LBP on AROM**	**LBP on flexion, extension and side glide and painful back, buttock and leg muscles**	**LBP on flexion, extension and side glide and painful back muscles**	**LBP and leg pain on AROM, SI joint dysfunction, painful buttock and leg muscles**	**Leg pain on AROM, neurological signs, pain on extension/rotation, trigger points**	

Bold text indicates characteristic variables which mainly identify the respective subgroups
IQR=inter quartile range, ROM = range of motion, w/wo=with or without
Symbols and light lines: Variables which have similar scoring pattern across subgroups representing an overall feature:
*Active range of motion (AROM)
** Neurology
***SI-joint dysfunction

Table 3f. Descriptive presentation of the patient subgroups based on variables from the psychology domain, single-stage LCA

Subgroup / Variable	SS 1	SS 2	SS 3	SS 4	SS 5	SS 6	SS 7	Scale
**Treatment not essential**	Few believe treatment is not essential to reduce pain (IQR=0-5)	Few believe treatment is not essential to reduce pain (IQR=1-5)	Few believe treatment is not essential to reduce pain (IQR=0-5)	Few believe treatment is not essential to reduce pain (IQR=0-5)	Few believe treatment is not essential to reduce pain (IQR=0-7)	Few believe treatment is not essential to reduce pain (IQR=0-5)	Few believe treatment is not essential to reduce pain (IQR=0-5)	**Continuous** 0=completely disagree 10=completely agree
**Sleep less well (RMDQ-15)***	Half sleep less well (57%)	Few sleep less well (21%)	**Many sleep less well (71%)**	**Many sleep less well (77%)**	Half sleep less well (46%)	**Many sleep less well** (76%)	**Many sleep less well (75%)**	**Dichotomous** 0=no 1=yes
**Had trouble sleeping at night (MDI-9)***	Few had trouble sleeping (IQR=0-2)	Very few had trouble sleeping (IQR=0-1)	Few had trouble sleeping (IQR=0-1)	**Half had trouble sleeping (IQR=1-4)**	Few had trouble sleeping (IQR=0-1)	**Half had trouble sleeping (IQR=1-4)**	Few had trouble sleeping (IQR=1-2)	**Ordinal** 0=at no time 5=all the time
**Able to decrease pain****	Highly able to decrease pain (IQR=2-5)	Highly able to decrease pain (IQR=1-4)	Moderately able to decrease pain (IQR=2-6)	Moderately able to decrease pain (IQR=3-6)	Moderately able to decrease pain (IQR=2-6)	Moderately able to decrease pain (IQR=5-7)	Moderately able to decrease pain (IQR=2-5)	**Continuous** 0=Completely 10=Not at all
**Work might harm back (FABQ-11)****	Very few believe work will harm Disagree=85% Unsure=11% Agree=4%	Very few believe work will harm Disagree=74% Unsure=12% Agree=15%	Few believe work will harm Disagree=48% Unsure=21% Agree=31%	Few believe work will harm Disagree=50% Unsure=25% Agree=26%	**Many believe work will harm** Disagree=8% Unsure=21% Agree=72%	**Many believe work will harm** Disagree=12% Unsure=27% Agree=61%	Few believe work will harm Disagree=55% Unsure=21% Agree=24%	**Ordinal**ǂ 0=completely disagree 3=unsure 6=completely agree
**Should not do normal work with present pain (FABQ-12)****	Very few believe they should not do normal work Disagree=91% Unsure=6% Agree=3%	Very few believe they should not do normal work Disagree=96% Unsure=3% Agree=1%	**Half believe they should not do normal work** Disagree=27% Unsure=22% Agree=51%	Very few believe they should not do normal work Disagree=65% Unsure=21% Agree=14%	**Half believe they should not do normal work** Disagree=26% Unsure=33% Agree=42%	**Many believe they should not do normal work** Disagree=8% Unsure=14% Agree=77%	Very few believe they should not do normal work Disagree=63% Unsure=19% Agree=18%	**Ordinal**ǂ 0=completely disagree 3=unsure 6=completely agree
**Cannot work till pain is treated (FABQ-14)****	Very few cannot work Disagree=93% Unsure=2% Agree=5%	Very few cannot work Disagree=96% Unsure=2% Agree=1%	**Many cannot work** Disagree=21% Unsure=19% Agree=60%	Few cannot work Disagree=70% Unsure=9% Agree=21%	Few cannot work Disagree=61% Unsure=13% Agree=25%	**Many cannot work** Disagree=13% Unsure=10% Agree=77%	Very few cannot work Disagree=73% Unsure=10% Agree=18%	**Ordinal**ǂ 0=completely disagree 3=unsure 6=completely agree
**Feel socially isolated*****	Very few feel socially isolated (12%)	Few feel socially isolated (22%)	Very few feel socially isolated (13%)	**Half feel socially isolated (51%)**	Few feel socially isolated (24%)	**Half feel socially isolated (51%)**	Few feel socially isolated (28%)	**Dichotomous** 0=Not at all isolated 1=little to quite isolated
**Negative recovery belief*****	Very few with negative recovery belief (17%)	Few with negative recovery belief (24%)	Very few with negative recovery belief (10%)	**Half with negative recovery belief (42%)**	**Few with negative recovery belief (39%)**	Few with negative recovery belief (39%)	Few with negative recovery belief (35%)	**Dichotomous** 0=Likely to recover 1=Unsure-Not at all likely
**Not enjoyed things used to enjoy (SBT-8)** ¤	Few have not enjoyed usual things (33%)	Very few have not enjoyed usual things (12%)	Half have not enjoyed usual things (40%)	**Many have not enjoyed usual things (63%)**	Few have not enjoyed usual things (32%)	**Many have not enjoyed usual things (74%)**	Half have not enjoyed usual things (41%)	Dichotomous 0=disagree 1=agree
**Worrying thoughts a lot of**	Very few worried	Very few worried	Very few worried	**Half worried**	Very few worried	**Half worried**	Very few worried	Dichotomous

**the time (SBT-6) ¤**	(9%)	(8%)	(17%)	**(55%)**	(18%)	**(58%)**	(19%)	0=disagree<br>1=agree
**Lost interest in daily activities (MDI-2) ¤**	Very few have lost interest all the time, few some of the time<br><br>At no time=61%<br>Some of the time=33%<br>Slightly-all the time=5%	Very few have lost interest all the time, few some of the time<br><br>At no time=75%<br>Some of the time=23%<br>Slightly-all the time=3%	Very few have lost interest all the time, few some of the time<br><br>At no time=58%<br>Some of the time=38%<br>Slightly-all the time=5%	**Half have lost interest all the time, few some of the time**<br><br>At no time=8%<br>Some of the time=36%<br>Slightly-all the time=56%	Very few have lost interest all the time, half some of the time<br><br>At no time=45%<br>Some of the time=43%<br>Slightly-all the time=12%	**Half have lost interest all the time, half some of the time**<br><br>At no time=12%<br>Some of the time=41%<br>Slightly-all the time=47%	Very few have lost interest all the time, half some of the time<br><br>At no time=42%<br>Some of the time=41%<br>Slightly-all the time=18%	**Ordinal**<br>0=at no time<br>1=some of the time<br>2=slightly-all the time
**Felt very restless/subdued/slowed down (MDI-8) ¤**	Very few have felt restless all the time, half some of the time<br><br>At no time=41%<br>Some of the time=52%<br>Slightly-all the time=7%	Very few have felt restless all the time, few some of the time<br><br>At no time=57%<br>Some of the time=39%<br>Slightly-all the time=5%	Very few have felt restless all the time, half some of the time<br><br>At no time=37%<br>Some of the time=50%<br>Slightly-all the time=13%	**Many have felt restless all the time, few some of the time**<br><br>At no time=2%<br>Some of the time=29%<br>Slightly-all the time=70%	Few have felt restless all the time, half some of the time<br><br>At no time=26%<br>Some of the time=52%<br>Slightly-all the time=22%	**Half have felt restless all the time, few some of the time**<br><br>At no time=6%<br>Some of the time=39%<br>Slightly-all the time=55%	Few have felt restless all the time, half some of the time<br><br>At no time=26%<br>Some of the time=50%<br>Slightly-all the time=24%	**Ordinal**<br>0=at no time<br>1=some of the time<br>2=slightly-all the time
**Had a bad conscience (MDI-5) ¤**	Very few had bad conscience all the time, few some of the time<br><br>At no time=73%<br>Some of the time=26%<br>Slightly-all the time=2%	Very few had bad conscience all the time or some of the time<br><br>At no time=80%<br>Some of the time=15%<br>Slightly-all the time=5%	Very few had bad conscience all the time, few some of the time<br><br>At no time=65%<br>Some of the time=29%<br>Slightly-all the time=6%	**Half had bad conscience all the time, few some of the time**<br><br>At no time=20%<br>Some of the time=30%<br>Slightly-all the time=50%	Very few had bad conscience all the time, few some of the time<br><br>At no time=70%<br>Some of the time=24%<br>Slightly-all the time=6%	**Half had bad conscience all the time, few some of the time**<br><br>At no time=23%<br>Some of the time=30%<br>Slightly-all the time=47%	Very few had bad conscience all the time, few some of the time<br><br>At no time=61%<br>Some of the time=31%<br>Slightly-all the time=8%	**Ordinal**<br>0=at no time<br>1=some of the time<br>2=slightly-all the time
**Felt low in spirits/sad (MDI-1) ¤**	Very few had felt sad all the time, few some of the time<br><br>At no time=72%<br>Some of the time=26%<br>Slightly-all the time=2%	Very few had felt sad all the time, few some of the time<br><br>At no time=68%<br>Some of the time=31%<br>Slightly-all the time=1%	Very few had felt sad all the time, few some of the time<br><br>At no time=62%<br>Some of the time=37%<br>Slightly-all the time=1%	**Few had felt sad all the time, half some of the time**<br><br>At no time=6%<br>Some of the time=58%<br>Slightly-all the time=36%	Very few had felt sad all the time, half some of the time<br><br>At no time=53%<br>Some of the time=45%<br>Slightly-all the time=2%	**Few had felt sad all the time, half some of the time**<br><br>At no time=16%<br>Some of the time=48%<br>Slightly-all the time=36%	Very few had felt sad all the time, half some of the time<br><br>At no time=41%<br>Some of the time=55%<br>Slightly-all the time=4%	**Ordinal**<br>0=at no time<br>1=some of the time<br>2=slightly-all the time
**Felt lacking in energy and strength (MDI-3) ¤**	Very few felt lacking in energy some of or all the time<br>(IQR=0-1)	Very few felt lacking in energy some of or all the time<br>(IQR=0-1)	Very few felt lacking in energy some of or all the time<br>(IQR=0-1)	**Half felt lacking in energy some of or all the time**<br>**(IQR=2-4)**	Few felt lacking in energy some of or all the time<br>(IQR=1-2)	Half felt lacking in energy some of or all the time<br>(IQR=1-4)	Few felt lacking in energy some of or all the time<br>(IQR=1-2)	**Ordinal**<br>0=at no time<br>5=all the time
**Felt less self-confident (MDI-4) ¤**	Very few had felt less self-confident all the time or some of the time<br><br>At no time=87%<br>Some of the time=12%<br>Slightly-all the time=1%	Very few had felt less self-confident all the time or some of the time<br><br>At no time=85%<br>Some of the time=13%<br>Slightly-all the time=2%	Very few had felt less self-confident all the time, few some of the time<br><br>At no time=79%<br>Some of the time=20%<br>Slightly-all the time=1%	**Few had felt less self-confident all the time, half some of the time**<br><br>At no time=25%<br>Some of the time=41%<br>Slightly-all the time=35%	Very few had felt less self-confident all the time, few some of the time<br><br>At no time=73%<br>Some of the time=22%<br>Slightly-all the time=5%	**Few had felt less self-confident all the time or some of the time**<br><br>At no time=33%<br>Some of the time=33%<br>Slightly-all the time=33%	Very few had felt less self-confident all the time or some of the time<br><br>At no time=77%<br>Some of the time=18%<br>Slightly-all the time=5%	**Ordinal**<br>0=at no time<br>1=some of the time<br>2=slightly-all the time
**Had difficulty in concentrating (MDI-7) ¤**	Very few had difficulty in concentrating all the time or some of the time<br><br>At no time=87%<br>Some of the time=11%<br>Slightly-all the time=2%	Very few had difficulty in concentrating all the time or some of the time<br><br>At no time=86%<br>Some of the time=13%<br>Slightly-all the time=1%	Very few had difficulty in concentrating all the time, few some of the time<br><br>At no time=72%<br>Some of the time=24%<br>Slightly-all the time=4%	**Few had difficulty in concentrating all the time, half some of the time**<br><br>At no time=27%<br>Some of the time=47%<br>Slightly-all the time=27%	Very few had difficulty in concentrating all the time, few some of the time<br><br>At no time=73%<br>Some of the time=22%<br>Slightly-all the time=5%	**Few had difficulty in concentrating all the time or some of the time**<br><br>At no time=42%<br>Some of the time=29%<br>Slightly-all the time=30%	Very few had difficulty in concentrating all the time, few some of the time<br><br>At no time=58%<br>Some of the time=32%<br>Slightly-all the time=9%	**Ordinal**<br>0=at no time<br>1=some of the time<br>2=slightly-all the time
**Have suffered from reduced/increased appetite (MDI-10) ¤**	Very few had reduced/increased appetite all the time or some of the time	Very few had reduced/increased appetite all the time or some of the time	Very few had reduced/increased appetite all the time, few some of the time	**Few had reduced/increased appetite all the time or some of the time**	Very few had reduced/increased appetite all the time or some of the time	**Few had reduced/increased appetite all the time or some of the time**	Very few had reduced/increased appetite all the time and few some of the time	**Ordinal**<br>0=at no time<br>1=some of the time<br>2=slightly-all the time

	At no time=86%<br>Some of the time=10%<br>Slightly-all the time=3%	At no time=88%<br>Some of the time=9%<br>Slightly-all the time=3%	At no time=68%<br>Some of the time=24%<br>Slightly-all the time=8%	At no time=39%<br>Some of the time=34%<br>Slightly-all the time=27%	At no time=78%<br>Some of the time=12%<br>Slightly-all the time=11%	At no time=39%<br>Some of the time=31%<br>Slightly-all the time=31%	At no time=68%<br>Some of the time=22%<br>Slightly-all the time=11%	
**More irritable with people than usual (RMDQ-17)** [¤¤]	Few more irritable<br>(20%)	Very few more irritable<br>(9%)	Few more irritable<br>(33%)	**Many more irritable<br>(69%)**	Few more irritable<br>(25%)	**Many more irritable<br>(68%)**	Half more irritable<br>(54%)	**Dichotomous**<br>0=no<br>1=yes
**Rubbing or holding areas that hurt/are uncomfortable (RMDQ-21)** [¤¤]	Few rub/hold areas that hurt<br>(36%)	Few rub/hold areas that hurt<br>(30%)	Half rub/hold areas that hurt<br>(41%)	**Many rub/hold areas that hurt<br>(63%)**	Few rub/hold areas that hurt<br>(35%)	**Many rub/hold areas that hurt<br>(70%)**	Half rub/hold areas that hurt<br>(49%)	**Dichotomous**<br>0=no<br>1=yes
**Not safe to be physically active (SBT-5)** [¤¤]	Very few believe it is not safe to be physically active<br>(14%)	Very few believe it is not safe to be physically active<br>(3%)	Few believe it is not safe to be physically active<br>(25%)	Very few believe it is not safe to be physically active<br>(16%)	Very few believe it is not safe to be physically active<br>(10%)	**Half believe it is not safe to be physically active<br>(49%)**	Very few believe it is not safe to be physically active<br>(11%)	Dichotomous<br>0=disagree<br>1=agree
**Terrible back pain, will never get better (SBT-7)** [¤¤]	Very few with terrible back pain<br>(2%)	Very few with terrible back pain<br>(2%)	Very few with terrible back pain<br>(1%)	Very few with terrible back pain<br>(14%)	Very few with terrible back pain<br>(6%)	Very few with terrible back pain<br>(19%)	Very few with terrible back pain<br>(10%)	Dichotomous<br>0=disagree<br>1=agree
**Often express concern (RMDQ-23)** [¤¤]	Very few express concern<br>(6%)	Very few express concern<br>(6%)	Very few express concern<br>(10%)	**Few express concern<br>(25%)**	Very few express concern<br>(17%)	**Few express concern<br>(31%)**	Half express concern<br>(15%)	**Dichotomous**<br>0=no<br>1=yes
**Physical activity might harm back (FABQ-3)**	Very few believe physical activity might harm back<br><br>Disagree=55%<br>Unsure=26%<br>Agree=19%	Very few believe physical activity might harm back<br><br>Disagree=52%<br>Unsure=31%<br>Agree=17%	Very few believe physical activity might harm back<br><br>Disagree=41%<br>Unsure=41%<br>Agree=18%	Very few believe physical activity might harm back<br><br>Disagree=49%<br>Unsure=35%<br>Agree=17%	**Few believe physical activity might harm back**<br><br>Disagree=26%<br>Unsure=37%<br>**Agree=37%**	**Few believe physical activity might harm back**<br><br>Disagree=24%<br>Unsure=42%<br>**Agree=34%**	Very few believe physical activity might harm back<br><br>Disagree=59%<br>Unsure=25%<br>Agree=16%	**Ordinal**[ǂ]<br>0=completely disagree<br>3=unsure<br>6=completely agree
**Should not do physical activity which (might) make pain worse (FABQ-4)**	Half believe avoiding physical activity is better<br><br>Disagree=25%<br>Unsure=22%<br>Agree=53%	Half believe avoiding physical activity is better<br><br>Disagree=31%<br>Unsure=26%<br>Agree=43%	Many believe avoiding physical activity is better<br><br>Disagree=14%<br>Unsure=22%<br>Agree=64%	Half believe avoiding physical activity is better<br><br>Disagree=27%<br>Unsure=23%<br>Agree=50%	Many believe avoiding physical activity is better<br><br>Disagree=15%<br>Unsure=19%<br>Agree=67%	Many believe avoiding physical activity is better<br><br>Disagree=8%<br>Unsure=22%<br>Agree=70%	Half believe avoiding physical activity is better<br><br>Disagree=29%<br>Unsure=28%<br>Agree=43%	**Ordinal**[ǂ]<br>0=completely disagree<br>3=unsure<br>6=completely agree
**Subgroup description**	**Sleep issues**	**The uncomplicated psychological profile**	**Pain-related concerns and sleep issues**	**Psychologically affected without pain-related concerns**	**Pain-related concerns and negative recovery beliefs**	**The complicated psychological profile**	**Sleep issues and catastrophising**	

Bold text indicates characteristic variables which mainly identify the respective subgroups
[ǂ] Trichotomised for the descriptive analysis:0=disagree (0-2), 1=unsure (3), 2=agree (4-6)
IQR = interquartile range; RMDQ = Roland-Morris Disability Questionnaire; FABQ, Fear-Avoidance Beliefs Questionnaire; SBT = STarT Back Tool; MDI = Major Depression Inventory
Symbols and light lines: Variables which have similar scoring pattern across subgroups representing an overall feature:
*Sleep issues
** pain-related concerns
***social isolation / negative recovery belief
[¤] depressive mood
[¤¤] catastrophising
